# Novel Benzimidazole-Endowed Chalcones as α-Glucosidase and α-Amylase Inhibitors: An Insight into Structural and Computational Studies

**DOI:** 10.3390/molecules29235599

**Published:** 2024-11-27

**Authors:** Prashasthi V. Rai, Ramith Ramu, P. Akhileshwari, Sudharshan Prabhu, Nupura Manish Prabhune, P. V. Deepthi, P. T. Anjana, D. Ganavi, A. M. Vijesh, Khang Wen Goh, Mohammad Z. Ahmed, Vasantha Kumar

**Affiliations:** 1 Department of PG Studies and Research in Chemistry, Sri Dharmasthala Manjunatheshwara College (Autonomous), Ujire 574240, Karnataka, India; prashasthi1316@gmail.com; 2 Department of Biotechnology and Bioinformatics, School of Life Sciences, JSS Academy of Higher Education and Research, Mysuru 570015, Karnataka, India; ramith.gowda@gmail.com; 3 PG Department of Physics, JSS College of Arts, Commerce and Science, Ooty Road, Mysuru 570025, Karnataka, India; akhila@uomphysics.net; 4 Department of Cellular and Molecular Biology, Manipal School of Life Sciences, Manipal Academy of Higher Education, Manipal 576104, Karnataka, India; sudharshan.prabhu@manipal.edu (S.P.);; 5 Department of Chemistry, Nehru Arts and Science College (Affiliated to Kannur University), Kanhangad 671314, Kerala, India; deepthi@nasc.ac.in; 6 Department of Chemistry, Krishna Menon Memorial Government Women’s College (Affiliated to Kannur University), Pallikkunnu, Kannur 670004, Kerala, India; 7 Department of Chemistry, Sri Dharmasthala Manjunatheshwara College (Autonomous), Ujire 574240, Karnataka, India; ganavijain@gmail.com; 8 P.G. Department of Chemistry, Payyanur College, Payyanur, Kannur University, Kannur 670327, Kerala, India; vijeshnambisan82@gmail.com; 9 Faculty of Data Science and Information Technology, INTI International University, Nilai 71800, Malaysia; khangwen.goh@newinti.edu.my; 10 Department of Pharmacognosy, College of Pharmacy, King Saud University, Riyadh 11451, Saudi Arabia; mahmed4@ksu.edu.sa; 11 Department of Studies and Research in Chemistry, Mangalore University, Mangalagangotri 574199, Karnataka, India

**Keywords:** benzimidazoles, chalcones, crystal structure, α-glucosidase, α-amylase, docking studies, molecular dynamic simulation

## Abstract

In search of novel antidiabetic agents, we synthesized a new series of chalcones with benzimidazole scaffolds by an efficient ‘one-pot’ nitro reductive cyclization method and evaluated their α-glucosidase and α-amylase inhibition studies. The ‘one-pot’ nitro reductive cyclization method offered a simple route for the preparation of benzimidazoles with excellent yield and higher purity compared to the other conventional acid- or base-catalyzed cyclization methods. ^1^H, ^13^C NMR, IR, and mass spectrum data were used to characterize the compounds. Single-crystal XRD data confirmed the 3D structure of compound **7c,** which was crystalized in the P$\bar{1}$ space group of the triclinic crystal system. Hirshfeld surface analysis validates the presence of O-H..O, O-H…N, and C-H…O intermolecular hydrogen bonds. From the DFT calculations, the energy gap between the frontier molecular orbitals in **7c** was found to be 3.791 eV. From the series, compound **7l** emerged as a potent antidiabetic agent with IC_50_ = 22.45 ± 0.36 µg/mL and 20.47 ± 0.60 µg/mL against α-glucosidase and α-amylase enzymes, respectively. The in silico molecular docking studies revealed that compound **7l** has strong binding interactions with α-glucosidase and α-amylase proteins. Molecular dynamics studies also revealed the stability of compound **7l** with α-glucosidase and α-amylase proteins.

## 1. Introduction

Diabetes is one of the most serious illnesses in the world and arises because of inadequate insulin generation by the pancreas or ineffective usage of insulin by the body. The hormone insulin is essential for controlling blood sugar levels and ensuring cells’ uptake of glucose to be used as an energy source. Type 1 diabetes is a result of inadvertent targeting and destruction of the pancreatic beta cells, which generate insulin. Conversely, Type 2 diabetes is primarily seen in adults, though it can also affect children. Type 2 diabetes arises due to the inefficient use of insulin by the body, and over time, the pancreas may not produce enough insulin. Increased rates of obesity and overweight are among the reasons contributing to this surge [1,2]. Worldwide, there was a drastic increase in diabetes in adults with 9.3% global diabetes prevalence in 2019, and is expected to rise to 10.2% by 2030 and 10.9% by 2045, which is roughly around 700 million [3].

**Figure 1:**
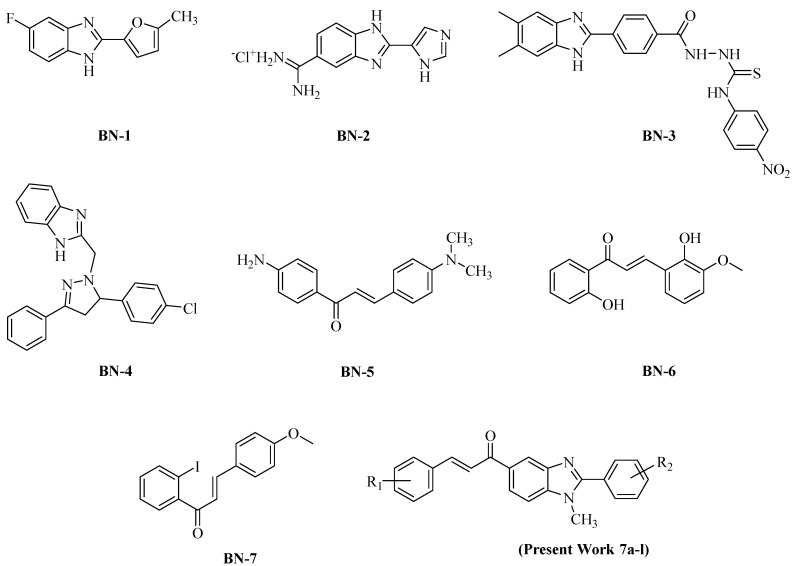
Reported antidiabetic agents containing benzimidazole (**BN-1–BN-4**), chalcone moiety (**BN-5–BN-7**), and molecules synthesized in present work (**7a–l**).

Benzimidazole-based derivatives were regarded as highly promising scaffolds in the field of medicinal chemistry with a vast number of bioactivities [4]. Benzimidazole, being one of the important classes of nitrogenous heterocycles bearing two nitrogen atoms in the imidazole ring, has exhibited a wide range of biological activities. These derivatives are found to be associated with antimicrobial [5], anti-inflammatory [6], anticancer [7], antiviral [8], anticonvulsant [9], antioxidant activities [10], and antidiabetic properties [11]. Benzimidazole nucleus is also found to be the most promising entity in anti-diabetic research. Studies have identified benzimidazoles with different antidiabetic activity; namely, compounds **BN-1** as the α-amylase inhibitor [12], **BN-2** as the DPP III inhibitor [13], and **BN-3** and **BN-4** as an effective inhibitor of α-glucosidase [14,15] (Figure 1). Moreover, benzimidazole and its derivatives have been investigated for their ability to act as an antidiabetic agent by targeting various mechanisms involved in diabetes pathophysiology and, hence, they remain a promising class of fused heterocycles in the identification of newer lead compounds in diabetic research.

On the other hand, chalcones are the important pharmacophores possessing almost all types of biological activities. Chalcones have gained much attention due to their presence in many natural products [16]. Due to their diverse applications, these molecules have also gained substantial consideration in medicinal chemistry. Over the past couple of decades, a substantial amount of work has been carried out in identifying newer simple chalcones or heterocyclic conjugated chalcones for various pharmacological properties. Various heterocycle-endowed chalcones are found to possess antimicrobial [17], antitubercular [18], anti-inflammatory [19], anticancer [20], antioxidant [21], antiviral [22], and anticonvulsant [23] activities. Moreover, chalcones are also explored extensively in antidiabetic research. Numerous studies have identified the heterocycles with chalcone units as excellent anti-diabetic agents, such as **BN-5** as an aldose reductase inhibitor **and BN-6** and **BN-7** with in vivo antidiabetic activity [24,25,26].

Based on the medicinal benefits of benzimidazoles and chalcones, and in view of our ongoing research in the benzimidazole class of compounds [27,28,29] and in continuation of research on heterocyclic antidiabetic compounds [30,31], a new series of benzimidazole-containing chalcone units at the 5th position were synthesized. Single-crystal X-ray diffraction (SC-XRD), Hirshfeld surface analysis, density functional theory (DFT) calculations, and molecular electrostatic potential (MEP) analysis were carried out. All the final compounds (**7a–l**) were screened for α-glucosidase and α-amylase inhibition studies. Further, cytotoxicity studies of the most potent compounds were carried out on normal HEK293 cell lines. Molecular docking and molecular dynamic simulation studies were carried out for the target compounds to understand the plausible binding interaction and stability with the α-glucosidase and α-amylase proteins.

## 2. Results and Discussion

### 2.1. Chemistry

**Scheme 1:**
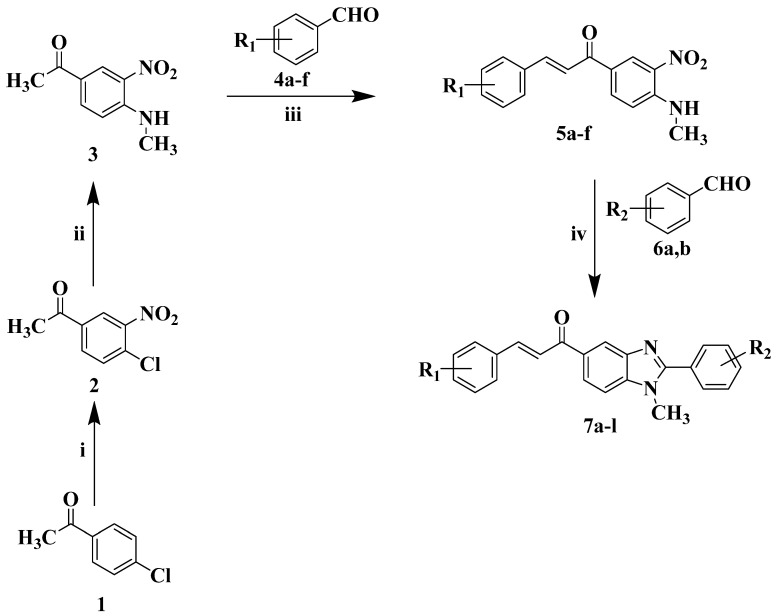
Synthesis of benzimidazole-endowed chalcone derivatives (**7a–l**). Reagents and conditions: (i) Conc. HNO_3_, Conc.H_2_SO_4_; (ii) methylamine, triethylamine, DMF, RT, 12 h; (iii) 30% NaOH, ethanol, RT, 6–8 h; (iv) sodium dithionite, DMSO, 90 °C, 3–4 h.

Novel benzimidazole-endowed chalcones (**7a–l**) were synthesized according to Scheme 1. The 4-chloro-3-nitroacetophenone (**2**) was prepared by the nitration of 4-chloroacetophenone (**1**) with a slight modification of the literature report [32]. Compound **2,** upon reaction with methylamine in the presence of triethylamine in DMF solution, afforded 1-(4-(methylamino)-3-nitrophenyl) ethan-1-one (**3**). In the next step, various chalcones (**5a–f)** were prepared by reacting compound **3** with different substituted benzaldehydes (**4a–f**) using 30% NaOH in ethanol solution. Finally, target benzimidazole derivatives (**7a–l**) were prepared by the ‘one-pot’ nitro reductive cyclization of compounds (**5a–f)** with two aromatic aldehydes (**6a,b**) using sodium dithionite as a reducing agent in DMSO solution. All the final products (**7a–l**) were obtained in very good yield and high purity. The nitro reductive cyclization reaction was completed within 3–4 h, indicating its efficiency over other conventional acids of base-catalyzed cyclization methods.

### 2.2. XRD Data and Molecular Geometry of Compound ***7c***

**Figure 2:**
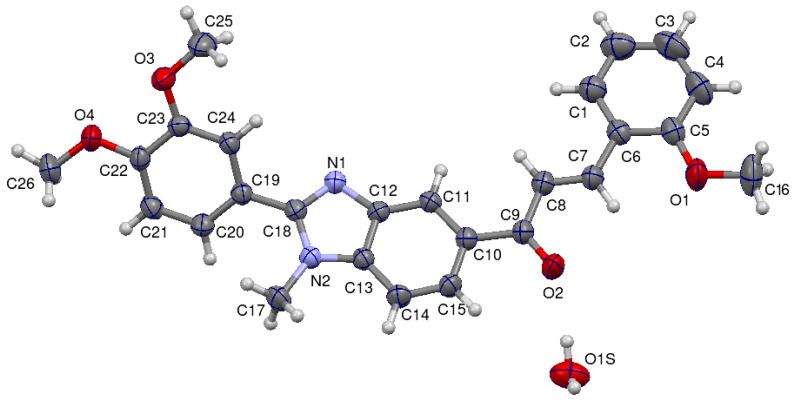
Thermal displacement ellipsoids of compound **7c** are drawn at the 50% probability.

Single-crystal X-ray diffraction data revealed the crystallization of compound **7c** in a triclinic crystal system with the space group P$\bar{1}$ and with the unit cell parameters *a* = 9.7376(15) Å, *b* = 11.374(2) Å, *c* = 11.481(2) Å, α = 79.140(9)°, β = 85.345(9)°, and γ = 65.010(8)°. The crystal parameters of compound **7c** are given in Table S1. The thermal displacement ellipsoids drawn at 50% probability are shown in Figure 2. The selected bond lengths, bond angles, and torsion angles are tabulated in Tables S2, S3, and S4, respectively.

**Figure 3:**
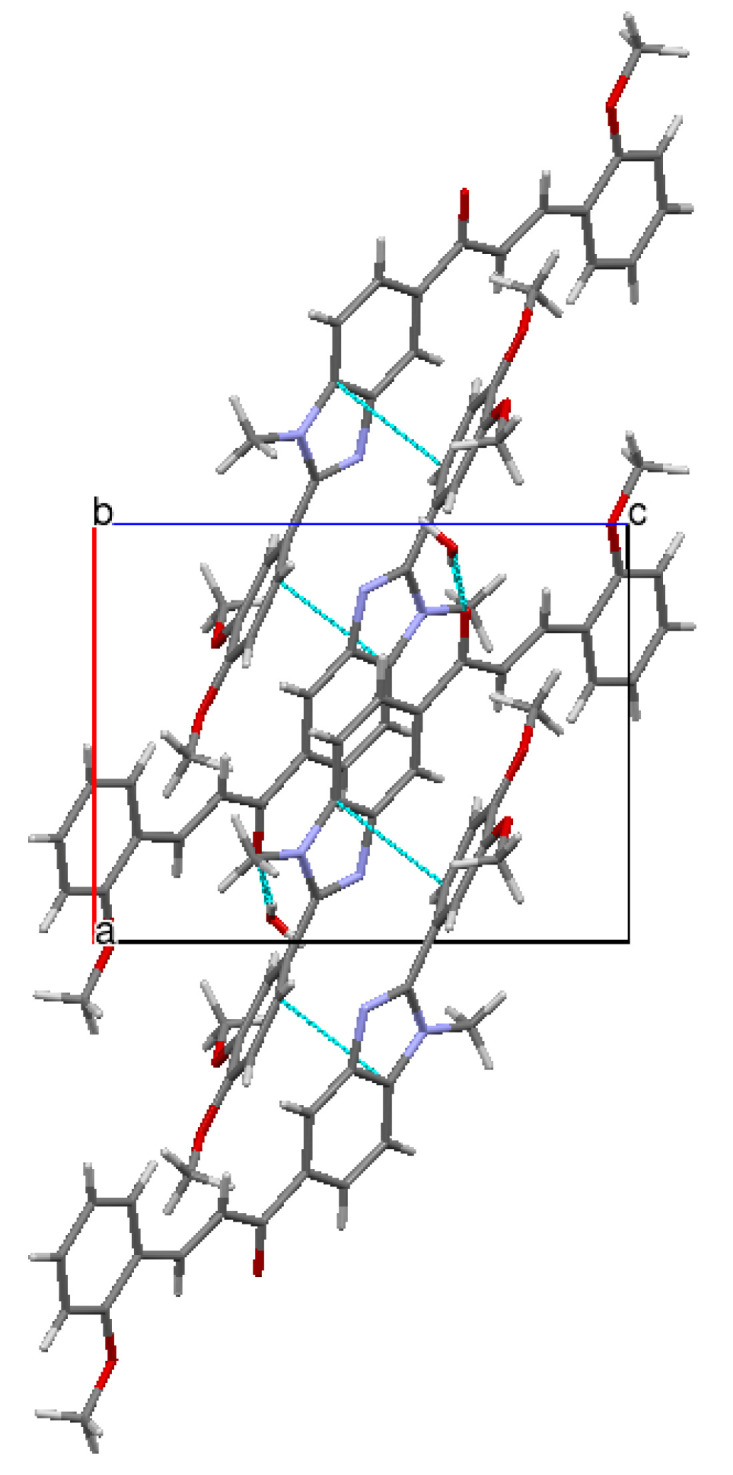
Packing of compound **7c** when viewed down along *b*-axis involved in hydrogen bond interactions.

The volume of compound **7c** is found to be 1131.9(3) Å^3^. A total of 310 unique parameters are found against a total of 5548 independent reflections. After several cycles, the final residual value is converged to 0.052. The goodness of fit on F^2^ was 0.949. The bond length of O1-C5 atoms is 1.358(3)Å, which indicates a typical single bond, while for O2-C9 atoms is 1.220 (3)Å, which indicates a double bond. The molecule adopts a stable trans configuration at the chalcone C=C, as indicated by the torsion angle of −176.8(2)° for C7-C8-C9-C10. This trans configuration is characterized by the double bonds between the C7–C8 (1.309(3) Å) and C9–O2 (1.220(3) Å). This trans arrangement provides stability, maintains a low-energy configuration, and promotes tighter packing within the crystal. Compound **7c** exhibits both intermolecular and intramolecular interactions. The compounds **7c** are connected by hydrogen bond interactions viz., O-H..O, O-H…N, and C-H…O intermolecular hydrogen bonds. The details of hydrogen bond interactions are listed in Table 1. The crystal structure is further stabilized by π-π and C-H…π interactions. The packing of compound **7c** viewed along the *b*-axis is shown in Figure 3.

### 2.3. Hirshfeld Surface, Shape Index Map, Curvedness Plot, and Fingerprint Plots of Compound ***7c***

**Figure 4:**
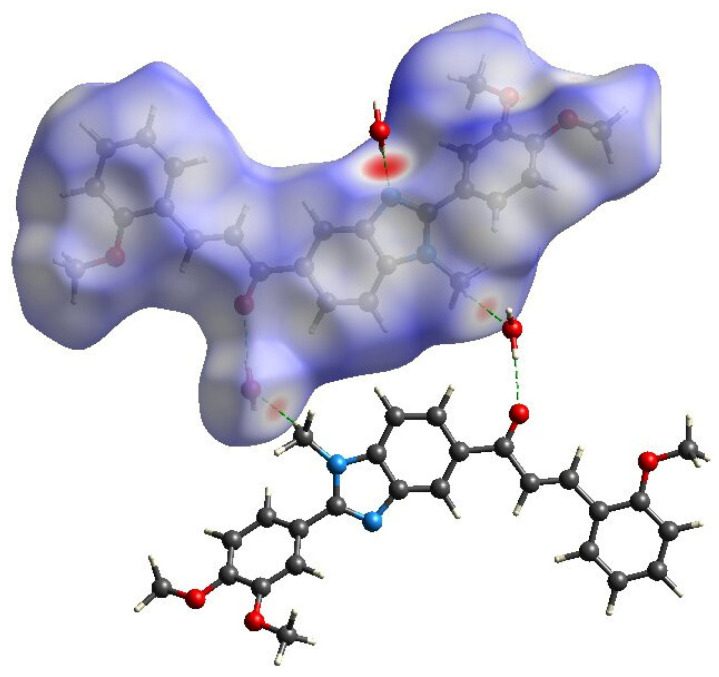
Hirshfeld surface of compound **7c** mapped with normalized distance contact.

Intermolecular interactions in the crystal were visualized by the Hirshfeld surface analysis tool [33]. It also was used to analyze the molecular packing and hydrogen bond interactions. CrystalExplorer17.5 software [34] was used to investigate and generate the Hirshfeld surfaces mapped over the *d*_norm_ surface. The intermolecular hydrogen bonds appear as red regions on the Hirshfeld surface. Hirshfeld surface mapped on *d*_norm_ is shown in Figure 4. The O-H…N interactions in the crystals are represented by bright regions on the Hirshfeld surface. The faint red color in Figure 4 indicates C-H…O interactions. Table 1 displays interactions consistent with the result obtained from XRD analysis.

**Figure 5:**
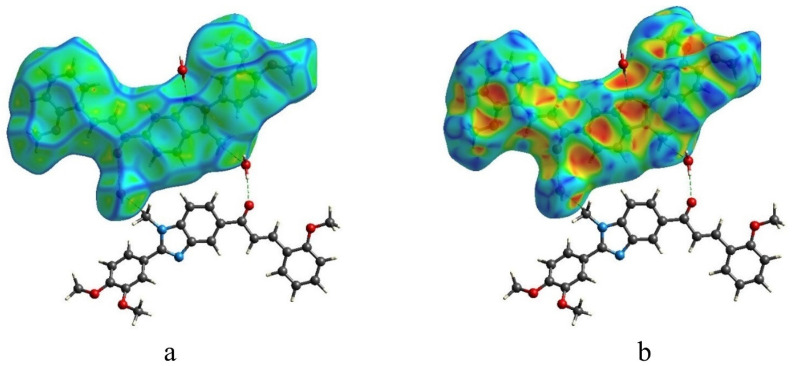
Hirshfeld surface (**a**): mapped with curvedness and (**b**): shape index map of compound **7c**.

The shape index plot and curvedness plot are used to determine the surface properties. The red and blue bumps on the shape index map indicate the π-π interactions involved in compound **7c**. The flat green surface separated by dark blue lines is an indication of planar stackings. Figure 5 depicts the π-π interactions on the shape index map (Figure 5b) and planar stackings on the curvedness map (Figure 5a) of compound **7c**.

**Figure 6:**
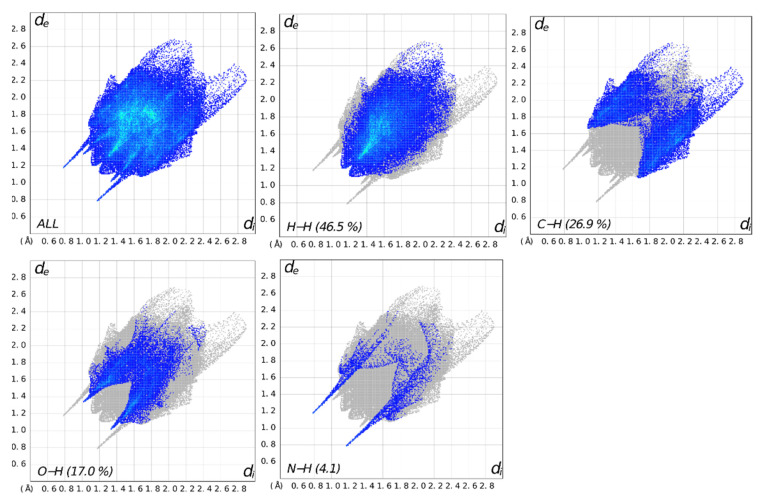
Fingerprint plots of individual hydrogen contacts of compound **7c**.

The fingerprint plots of compound **7c** are shown in Figure 6, which gives the summary of contributions from individual contacts to the Hirshfeld surface. The major contribution is from H-H (46.5%) contacts and the lowest contribution is from N-H/H-N (4.1%) contacts.

### 2.4. Frontier Molecular Orbital Studies of Compounds ***7c*** and ***7l***

**Figure 7:**
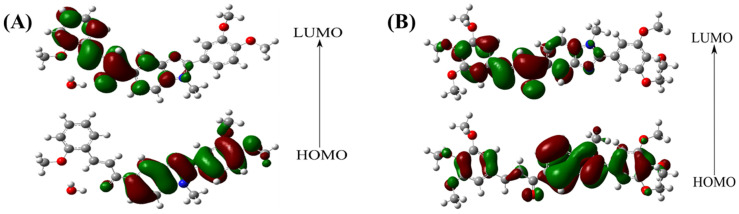
(**A**) HOMO/LUMO of compound **7c**; (**B**) HOMO/LUMO of compound **7l**.

Frontier molecular orbital (FMO) studies were carried out for crystal-structured compound **7c** and for the highly potent antidiabetic compound in the series **7l**. The energy difference between the HOMO and LUMO for compound **7c** is found to be 3.791 eV, indicating the soft nature of compound **7c**. The HOMO spreads all over the 3,4-dimethoxyphenyl ring and benzimidazole ring; LUMO is seen on 2-methoxyphenyl, chalcone, and partially on the benzene ring of compound **7c** (Figure 7A). Similarly, the energy difference between HOMO and LUMO for compound **7l** is found to be 4.1 eV. In compound **7l**, HOMO is localized over benzimidazole moiety and partially on the 3,4,5-trimethoxyphenyl ring, whereas the LUMO is observed on the 3,4,5-trimethoxyphenyl ring and chalcone moiety (Figure 7B). The molecular descriptors for compound **7c** and **7l** are computed and listed in Table 2. It is observed that compounds **7c** and **7l** are softer compared to similar molecules reported previously [35]. The electrophilic indexes (ω) of both molecules indicate that they are strong electrophiles, as their values are greater than 1.50 eV. In addition, the energy gap of the molecules suggests potential biological activity, as a narrower gap often correlates with a higher likelihood of interaction with biological targets [35].

### 2.5. Molecular Electrostatic Potential of Compound ***7c***

**Figure 8:**
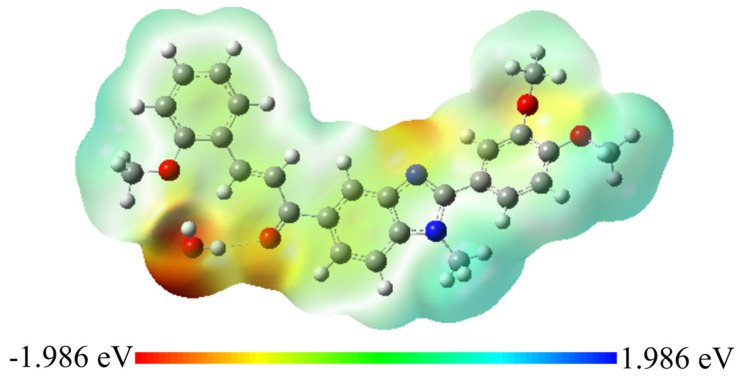
Molecular electrostatic potential map of compound **7c**.

Molecular electrostatic potential (MEP) represents the potential energy experienced by a charge around the molecule due to its electron density. This potential distribution helps to identify chemically reactive sites within the molecule, where regions of high or low potential are visualized with different colors. In Figure 8, areas of maximum negative electrostatic potential are shown in red, while areas of maximum positive electrostatic potential are shown in blue. The MEP values range from −1.986 eV to +1.986 eV, indicating the regions favorable for interactions. For this molecule, regions of high electron density, corresponding to areas of negative potential, are observed around the oxygen atom in the chalcone moiety, the water molecule, and, to a lesser extent, the nitrogen atom. These regions are likely sites for electrophilic attacks. The regions of positive potential are seen around hydrogen atoms. MEP result suggests regions of negative potential that could facilitate interactions with biological receptors, indicating potential sites of biological activity. These chemical reactive sites are likely favorable for hydrogen bond formation. The MEP results align with the XRD findings, indicating the presence of a C-H…O hydrogen bond. In this interaction, the carbon atom acts as the hydrogen bond donor, while the oxygen atom serves as the acceptor (see Table 1).

### 2.6. Biological Activity

#### 2.6.1. α-Glucosidase and α-Amylase Inhibition Studies

In vitro α- glucosidase and α- amylase inhibition studies were carried out for all the synthesized target compounds **7a–l**. Screened compounds **7a–l** exhibited different inhibitory activity with IC_50_ values from 20.47 ± 0.60 to 41.25 ± 0.37 µg/mL, and corresponding data are depicted in Table 3 along with standard drug acarbose.

Among the two series of compounds **7a–f** and **7g–l**, it is found that compound **7l** from the second series having 3,4,5-trimethoxyphenyl substitution at the second position and at the chalcone moiety demonstrated the highest inhibitory effects against both α-glucosidase and α-amylase enzymes with IC_50_ = 22.45 ± 0.36 µg/mL and 20.47 ± 0.60 µg/mL, respectively, which is much higher than the standard drug acarbose with IC_50_ = 35.05 ± 0.25 µg/mL and 38.58 ± 0.32 µg/mL, respectively. Similarly, compound **7e** from the first series with 3,4-dimethoxyphenyl substitution at the second position and at the chalcone unit exhibited excellent inhibitory activity with IC_50_ = 23.88 ± 0.55 µg/mL for α-glucosidase enzyme.

Further, from the first series, compounds **7c** with 2-methoxyphenyl substitution and **7d** with 4-methoxyphenyl substitution in the chalcone unit emerged as good inhibitors of α-glucosidase enzyme with IC_50_ values 25.21 ± 0.13 and 26.32 ± 0.52 µg/mL, respectively. However, from the first series, none of the compounds exhibited promising activity against the α-amylase enzyme. From the second series, compound **7j** with 4-methoxyphenyl substitution in the chalcone unit emerged as a good inhibitor of the α-glucosidase enzyme with IC_50_ values 27.21 ± 0.36 µg/mL. The other four compounds in the series, **7g–i** and **7k,** were less active towards the α-glucosidase enzyme. From the second series, compound **7i** with 2-methoxyphenyl and **7g** with 2-chlorophenyl substitution at the chalcone unit exhibited good α-amylase inhibition with IC_50_ values 24.21 ± 0.31 and 28.58 ± 0.37 µg/mL, respectively.

#### 2.6.2. Cytotoxicity on Normal HEK293 Cell Line

**Figure 9:**
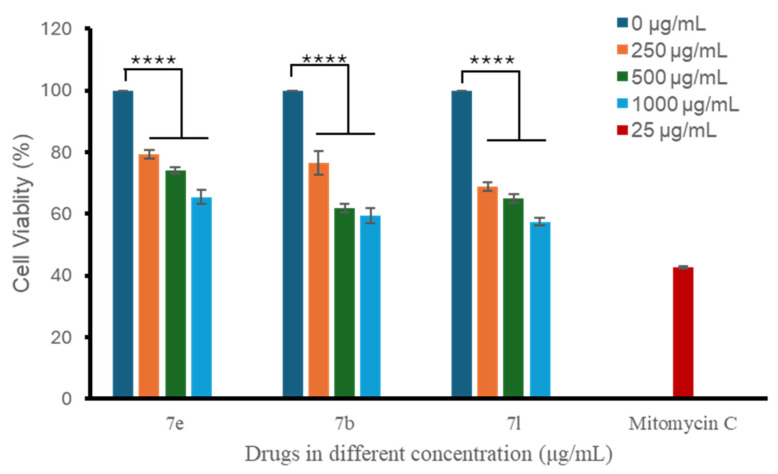
Cytotoxicity analysis of compounds **7b**, **7e,** and **7l** against HEK 293 cell lines. (The data are the mean (*n* = 3) ± SD. One-way ANOVA followed by Dunnett’s post hoc test. The significant levels **** *p* < 0.0001 when compared with control group (0 μg/mL). * Indicates statistically significant difference).

To evaluate the cytotoxicity of the potent compounds, **7b**, **7e**, and **7l** were screened for their cell viability on normal human kidney HEK293 cell lines by MTT assay [36]. All three compounds were screened at concentrations of 250, 500, and 1000 µg/mL. These results (Figure 9) indicate negligible cytotoxicity of the three compounds against HEK293 cell lines even at the highest concentration (1000 µg/mL). The results of the study indicated that these compounds are safer when compared to the standard drug Mitomycin, which showed around 60% cell death at 25 µg/mL. Hence, these compounds could be screened for further developmental studies. 

### 2.7. Molecular Docking on α-Glucosidase and α-Amylase Proteins

**Figure 10:**
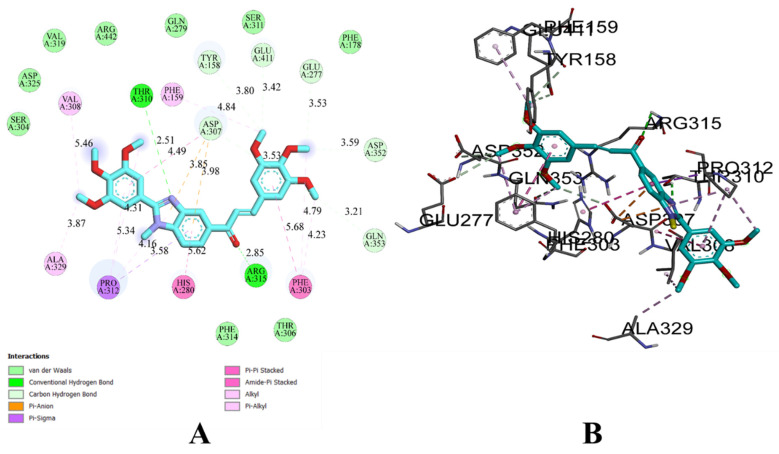
Two-dimensional (**A**) and three-dimensional (**B**) representation of compound **7l** interacting with the amino acids of α-glucosidase protein.

**Figure 11:**
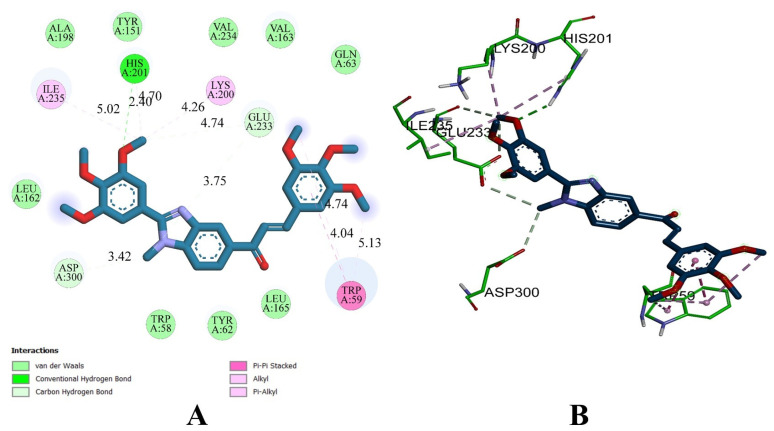
Two-dimensional (**A**) and three-dimensional (**B**) representation of compound **7l** interacting with the amino acids of α-amylase protein.

Docking studies were conducted to investigate the diverse binding interactions of the synthesized compounds **7a–l** with α-glucosidase and α-amylase proteins. Table 4 illustrates the various binding interactions observed between the synthesized compounds (**7a–l**) and α-glucosidase and α-amylase proteins. Figure 10 and Figure 11 illustrate the two-dimensional (2D) and three-dimensional (3D) representations of the binding interactions between compound **7l** with α- glucosidase and α-amylase, respectively.

Compound **7l** has shown a strong interaction with α-glucosidase protein with the highest binding affinity of −10 kcal/mol (Figure 10) and due to the presence of 23 interactions, which is a confirmation of the observed higher activity compared to other compounds in the series. It has exhibited two conventional hydrogen bonds and two π-π interactions with the α-glucosidase protein. The nitrogen atom of the benzimidazole ring forms a hydrogen bond with THR310 at a distance of 2.51 Å, and the carbonyl group of the chalcone unit forms a hydrogen bond with ARG315 at a distance of 2.85 Å. Further, this molecule also displayed two π-π interactions, where the benzene ring of benzimidazole binds to HIS280 and the 3,4,5-trimethoxyphenyl ring of chalcone unit with PHE303 at a distance of 5.62 and 5.68 Å, respectively. The 3,4,5-trimethoxyphenyl ring at the 2nd position is involved in a π–amide interaction with ASP307.

The docking study results of compound **7l** on α-amylase protein indicate that it has the highest binding affinity of −9.6 kcal/mol with a total of 13 interactions (Figure 11). Compound **7l** has strong hydrogen bonding between methoxy oxygen of 3,4,5-trimethoxyphenyl moiety at the 2nd position of benzimidazole and HIS201 at a distance of 2.40 Å. Other 3,4,5-trimethoxyphenyl rings exhibited strong π-π interactions with TRP59 at a distance of 4.04 Å. In addition to this, it has also bonded with α-amylase protein through van der Waals, carbon–hydrogen bonds, π–alkyl, and alkyl–alkyl interactions.

DFT results of compound **7l** are in agreement with the observed docking results on α-glucosidase and α-amylase proteins. The lower ionization potential (2.020 eV) value of compound **7l** indicates that it has better electron-donating properties, which in turn implies the better hydrogen bonding property of the molecule. These results are consistent with the earlier reported condensed heterocyclic system comprising benzimidazole and thiadiazole moieties [35].

### 2.8. Molecular Dynamic Simulation Studies

Docking simulations were validated through molecular dynamic (MD) simulations, which elucidated the dynamic behavior of protein–ligand complexes over time in a solvated environment. The MD study provided analyses of the protein–ligand complex’s RMSD, Rg, SASA, ligand RMSD, the total number of hydrogen bonds maintained by the ligand throughout the simulation, and the variation in secondary structure patterns between the protein and its complexes.

#### 2.8.1. MD Studies of Compound **7l** and Acarbose with α-Glucosidase Protein

**Figure 12:**
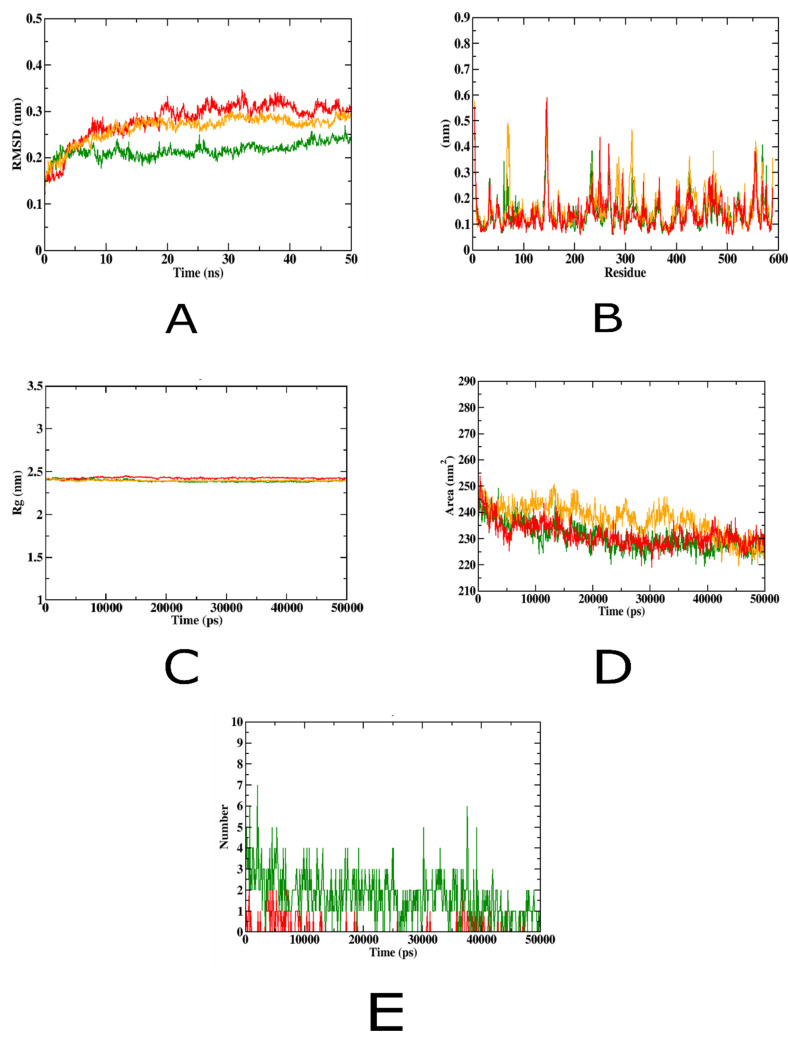
Visualization of MD trajectories of compound **7l** and acarbose complexed with α-glucosidase protein run for 50 ns. (**A**) Protein–ligand complex RMSD, (**B**) protein–ligand complex RMSF, (**C**) protein–ligand complex Rg, (**D**) protein–ligand complex SASA, (**E**) ligand hydrogen bonds (red: apoprotein; green: protein–compound **7l** complex; orange: protein–acarbose complex).

For α-glucosidase, both compound **7l** and acarbose remained within the inhibitor binding site throughout the simulation. Compound **7l** started to stabilize within 10 ns, while acarbose did not stabilize until 30 ns, according to the RMSD plot. Compound **7l** appeared to be stable throughout the simulation, as indicated by the RMSF analysis, which showed no anomalous variations. There were not many variations seen in the loop and terminal portions (600 residues) of the RMSF patterns for the apoprotein and the protein–compound **7l** complex. The protein–acarbose complex, on the other hand, showed more variations. The protein–compound **7l** complex’s SASA value significantly decreased as a result of compound **7l** being more compact, binding to the protein, as evidenced by the Rg plot when compared to acarbose. This drop in SASA was brought about by the effective binding of compound **7l** with the target protein α-glucosidase. Based on MD trajectory analysis, both compound **7l** and acarbose were stable within the inhibitor binding site of α-glucosidase. However, the trajectory analysis indicated that the acarbose was comparatively unstable despite occupying the same binding site as compound **7l**. Table 5 presents the MD trajectory values for α-glucosidase apoprotein and the protein complexed with compound **7l** and acarbose, while Figure 12 displays the trajectory plots from the simulation.

#### 2.8.2. MD Studies of Compound **7l** and Acarbose with α-Amylase Protein

Compound **7l** and acarbose both stayed inside the inhibitor binding site for α-amylase during the simulation. Compound **7l** showed signs of increasing stability after 10 ns, but acarbose remained unstable until 30 ns, according to the RMSD plot. For compound **7l**, the RMSF analysis showed no anomalous variations, suggesting that it remained stable over the simulation. There were not many variations seen in the loop and terminal portions (residues) of the RMSF patterns for the apoprotein and the protein–compound **7l** complex. On the other hand, there were greater oscillations in the protein–acarbose complex. Compound **7l** was more compactly attached to the protein than acarbose, as evidenced by the Rg plot, which resulted in a considerable drop in the SASA value of the protein–compound **7l** combination. This decline in SASA was brought about by the productive occupation.

**Figure 13:**
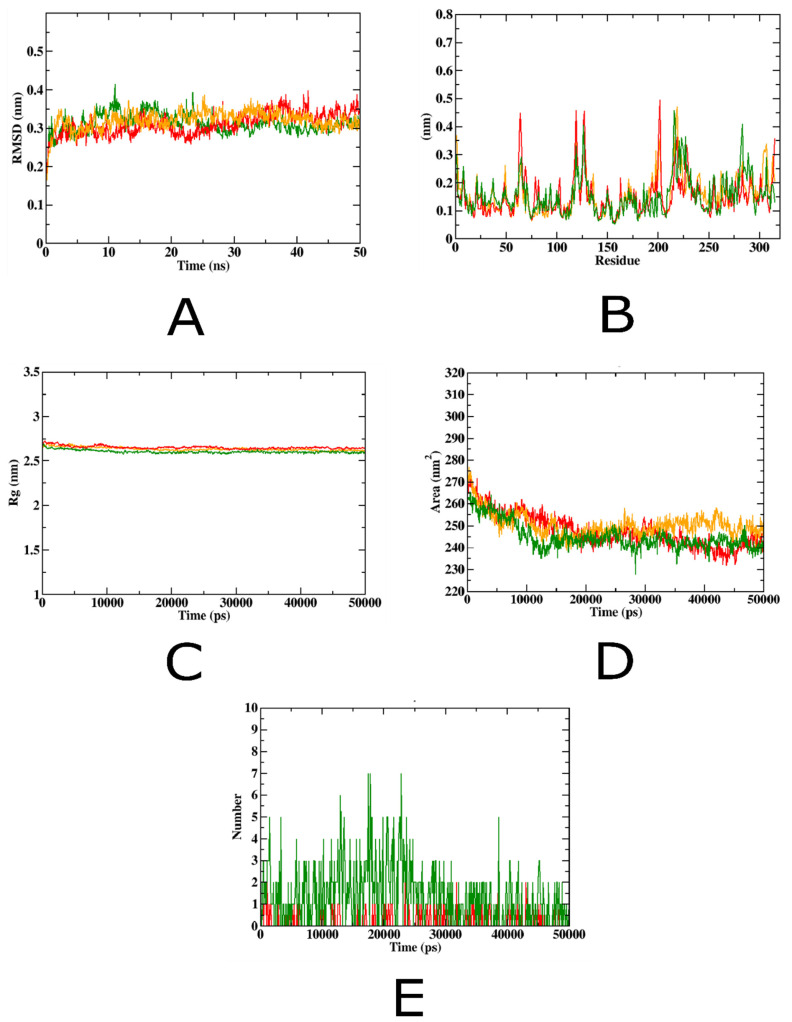
Visualization of MD trajectories of compound **7l** and acarbose complexed with α-amylase run for 50 ns. (**A**) Protein–ligand complex RMSD, (**B**) protein–ligand complex RMSF, (**C**) protein–ligand complex Rg, (**D**) protein–ligand complex SASA, and (**E**) ligand hydrogen bonds (red: apoprotein; green: protein–compound **7l** complex; orange: protein–acarbose complex).

Based on the MD trajectory analysis, both compound **7l** and acarbose were stable within the inhibitor binding site of α-amylase. However, the trajectory analysis indicated that acarbose was comparatively unstable despite occupying the same binding site as compound **7l**. Table 6 presents the MD trajectory values for α-amylase apoprotein and the protein complexed with compound **7l** and acarbose, while Figure 13 displays the trajectory plots from the simulation.

The MD simulation results of compound **7l** with α-glucosidase and α-amylase align with our previously reported compounds [37], and hence compound **7l** is considered a potential dual inhibitor of α-glucosidase and α-amylase.

## 3. Experimental

### 3.1. Materials and Measurements

All the chemicals and reagents used for this study were obtained from TCI (Tokyo, Japan), Sigma-Aldrich (St. Louis, MO, USA), NICE (Ra’anana, Israel), Spectrochem (Mumbai, India), and Loba Chemie company (Mumbai, India). The chemicals purchased were of analytical grade and used directly without further purification. The starting material 4-chloroacetophenone was purchased from TCI (98% purity), conc. nitric acid from NICE (70%), and conc. sulphuric acid from Loba Chemie (98%). The open capillary tube method was used to record the melting point of novel synthesized compounds, which were uncorrected. Reactions were monitored by thin-layer chromatography (mobile phase-ethyl acetate: hexane; 3:2) using the Merck 60F254 aluminum sheet embedded in the silica gel (Rahway, NJ, USA). Tensor 27, JEOL 400 MHz NMR (Tokyo, Japan), a 100 MHz spectrometer, and an Agilent Technology LC-mass spectrometer (Santa Clara, CA, USA) were used to record IR, ^1^H NMR, ^13^C NMR, and mass spectra of the compounds, respectively. Chemical shift values (δ) are expressed in parts per million (ppm) in relation to an internal standard tetramethylsilane. Signal multiplicities are expressed as singlet (s), doublet (d), triplet (t), and multiplet (m), while coupling constants (J) are expressed in Hertz (Hz). In CHNS Elemental Vario EL III, elemental analysis was performed.

### 3.2. Procedure for the Synthesis of 1-(4-(Methylamino)-3-Nitrophenyl)Ethan-1-One *(**3**)*

4-Chloro-3-nitroacetophenone (**2**) (0.010 moles), was prepared by a slight modification of the previously reported procedure from 4-chloroacetophenone [32]. The compound (**2**) was dissolved in DMF (20 mL). Triethylamine (0.015 moles) and methylamine (0.07 moles) were added and the reaction mixture was stirred at room temperature (RT) for 12 h. Upon completion of the reaction, it was quenched with ice, and a precipitated solid was filtered, washed with cold water, and dried overnight to obtain 1-(4-(methylamino)-3-nitrophenyl)ethan-1-one (**3**).

### 3.3. General Procedure for the Preparation of Chalcones *(**5a–f**)*

To a well-stirred mixture of compound **3** (1 eq.) and different substituted benzaldehydes (**4a–f**) (1 eq.) in ethanol (10 mL), 30% NaOH (10 mL) was added and stirred at RT for 6–8 h. Upon reaction completion, it was quenched with ice-cold water, and the solid was filtered and washed with cold water. The resulting chalcones (**5a–f**) were used as such for the next step. The spectral details for the selected compound **5a** are given below; the remaining compounds **5b–f** are detailed in the Supplementary Materials.

(E)-3-(3,4-dimethoxyphenyl)-1-(4-(methylamino)-3-nitrophenyl)prop-2-en-1-one (**5a**). Yield: 82–84%; M.p.: 157–159 °C; ^1^H NMR (400 MHz, CDCl_3_, δ in ppm): 8.90 (d, 1H, *J* = 2 Hz, Ar-H_2_), 8.44 (d, 1H, *J* = 4.4 Hz, Ar-H), 8.22 (dd, 1H, *J* = 9.2 and 2 Hz, Ar-H_6_), 7.79 (d, 1H, *J* = 15.6 Hz, chalcone =CH), 7.39 (d, 1H, *J* = 15.2 Hz, chalcone =CH), 7.26 (d, 1H, *J* = 1.6 Hz, Ar-H_2_ of 3,4-(OMe)_2_-Ph), 7.16 (d, 1H, *J* = 1.6 Hz, Ar-H_6_ of 3,4-(OMe)2-Ph), 6.94–6.88 (m, 2H, Ar-H_5_ of 3,4-(OMe)_2_-Ph and Ar-H_5_), 3.96 (s, 3H, OMe), 3.92 (s, 3H, OMe), 3.11 (d, 3H, *J* = 5.2 Hz, N-Me).

### 3.4. General Procedure for the Synthesis of Benzimidazole Derivatives *(**7a–l**)*

Different substituted chalcones **5a–f** (1 eq.) and different substituted benzaldehydes **6a,b** (1 eq.) were dissolved in DMSO. After adding sodium dithionite (3 eq.), the reaction mixture was stirred at 90–92 °C for 3–4 h. Upon reaction completion, it was poured into crushed ice, resulting in a solid that was filtered and washed with water. Crude products were recrystallized using DMF. The spectral details for the selected compound **7l** are given below; the remaining compounds **7a–k** are detailed in the Supplementary Materials.

(E)-1-(1-methyl-2-(3,4,5-trimethoxyphenyl)-1H-benzo[*d*]imidazol-5-yl)-3-(3,4,5-trimethoxyphenyl)prop-2-en-1-one (**7l**). Yield: 76–78%; M.p.: 218–220 °C; FTIR (KBr, cm^−1^): 3165 (=C-H), 3126 (=C-H), 2998 (=C-H), 2924 (=C-H), 1652 (C=O), 1609 (C=C and C=N), 1242 (O-CH_3_);^1^H NMR (400 MHz, DMSO, δ in ppm): 8.72 (s, 1H, benzimidazole-H_4_), 8.08–8.03 (m, 2H, benzimidazole-H_6_ and chalcone =CH), 7.74 (d, 1H, *J* = 8.8 Hz, benzimidazole-H_7_), 7.67 (d, 1H, *J* = 15.6 Hz, chalcone =CH), 7.24 (s, 2H, Ar-H_2,6_ of 4-(OMe)_3_-Ph), 7.12 (s, 2H, Ar-H_2,6_-3,4,5-(OMe)_3_-Ph), 3.94 (s, 3H, OMe), 3.85 (s, 6H, OMe), 3.84 (s, 6H, OMe), 3.72 (s, 3H, OMe), 3.67 (s, 3H, N-CH_3_); ^13^C NMR (100 MHz, CDCl_3_, δ in ppm): 188.3 (C=O), 155.0, 153.1 (Ar-C-O), 152.9 (Ar-C-O), 143.7 (=C), 142.0, 139.9 (Ar-C-O), 139.5, 138.9, 132.2, 130.4, 125.8, 124.9, 122.9, 121.4, 120.8, 110.8, 106.9, 106.4, 61.0 (O-CH_3_), 60.1 (O-CH_3_), 60.1 (O-CH_3_), 56.1 (O-CH_3_), 32.1 (N-CH_3_); LCMS (m/z):519.1 [M+1]; Anal. Calcd. for C_29_H_30_N_2_O_7_: C, 67.17; H, 5.83; N, 5.40; O, 21.60; found C, 67.12; H, 5.79; N, 5.45; O, 21.56.

### 3.5. X-Ray Crystallographic Study

A crystal of compound **7c** with a suitable dimension (0.20 × 0.25 × 0.30 mm^3^) was selected for the single-crystal X-ray diffraction experiment. X-ray intensity measurements for the compound **7c** were obtained at room temperature with a Bruker axs kappa apex2 CCD diffractometer (λ = 0.7107 Å). Raw data for absorption corrections were processed using Bruker Apex 2 software. Fourier techniques were used to solve the structure, followed by refinement with the SHELXL program [38]. For every non-hydrogen atom, anisotropic displacement parameters were included. The hydrogen atoms were placed geometrically, riding on their parent carbon atoms for other hydrogen atoms. The geometric calculations, such as bond lengths, bond angles, and torsion angles, were calculated using PLATON software (Version 130921, WEB: 11 November 2024) [39]. The thermal displacement ellipsoids and molecular packing diagrams were generated by Mercury 4.2 software [40].

### 3.6. Density Functional Theory

DFT studies for compounds **7c** and **7l** were carried out using Gaussian09 software [41] to determine the chemical reactivity and kinetic stability of the molecule by analyzing frontier molecular orbitals (HOMO and LUMO). The calculations were performed at the B3LYP level of theory with the 6-31+G(d,p) basis set [41]. A small energy gap between HOMO and LUMO implies high reactivity, while a large energy gap indicates lower reactivity [42].

### 3.7. Biological Studies

#### 3.7.1. In Vitro Carbohydrate Digesting Enzymes Inhibition Assays

The in vitro enzyme inhibition assays for the two enzymes, i.e., α-glucosidase and α-amylase, were performed according to the previous studies [43].

The yeast α-glucosidase inhibition assay was performed using pNPG as substrate. An amount of 100 µL of enzyme was then added to the mixture, and the reaction was incubated at 37 °C for 10 min. After adding 0.5 mM pNPG solution, the reaction continued at 37 °C for 20 min. The enzyme activity was determined by measuring the absorbance of the liberated p-nitrophenol at 405 nm using a microplate spectrophotometer. The results were expressed as a percentage of α-glucosidase inhibition, calculated using the formula “Inhibition (%) = (A control − A sample)/A control × 100”. The final results of the enzyme inhibition assay are represented as IC_50_ values which refers to the concentration required to block 50% of α-glucosidase activity under the test conditions.

The α-amylase inhibition activity was performed using type-VI B porcine pancreatic α-amylase, with starch as a substrate. The enzyme was dissolved in sodium phosphate buffer and the test compound was added at different concentrations. The activity was determined by adding 1% starch solution and incubating at 25 °C for 15 min. The reaction was terminated by adding a DNS reagent and kept in a boiling water bath for 5 min. The resulting mixture was diluted and absorbance was measured at 540 nm using a microplate spectrophotometer. The results were compared with a control, acarbose, and the IC_50_ values were determined by plotting the % inhibition of each compound relative to its concentration.

#### 3.7.2. In Vitro Cytotoxicity Assay on Normal HEK-293 Cell Line

The Human Embryonic Kidney (HEK-293) cell lines were cultured in Dulbecco’s Modified Eagle Medium (DMEM) supplemented with 10% fetal bovine serum (FBS). The cells were maintained at 37 °C in a humidified atmosphere of 5% CO_2_. The adherent cells were grown to 70–80% confluency and then harvested by trypsinization (0.25%). The test compounds were dissolved in DMSO and used for all the experiments. DMSO (0.1%) was used as vehicle control and cells cultured in a complete medium without compound served as control.

The most potent antidiabetic compounds, **7b**, **7e,** and **7l,** were then screened for their in vitro cytotoxic potential using 3-(4,5-Dimethylthiazol-2-yl)-2,5-Diphenyltetrazolium Bromide (MTT) assay [36]. Briefly, 5 × 10^3^ cells (HEK293 cells) were seeded and allowed to adhere overnight. The cells were treated with different concentrations of the compounds (250, 500, and 1000 µg/mL) for 48 h at 37 °C. Post 48 h, the cells were exposed to MTT (5 mg/mL in phosphate-buffered saline). The formazan crystals were dissolved in DMSO and the absorption was measured at 570 nm using a microplate reader (Tecan Spark, Switzerland). The percentage viability of the cells was determined.

### 3.8. Molecular Docking Studies

The crystal structures of α-glucosidase (PDB ID: 3A4A) and α-amylase (PDB ID: 1DHK) were obtained from the RCSB PDB database. The ligand preparation, preparation of protein targets, prediction of binding site, and molecular docking studies were performed according to the previously reported studies [44]. The molecular docking studies were performed using Autodock tools 1.5.7 through Autodock Vina.

### 3.9. Molecular Dynamics Simulation and Binding Free Energy Calculations

Molecular dynamics simulation was carried out as per the previous studies [45]. In brief, the molecular dynamics simulation was performed using GROMACS-2018.1 to determine the stability of the best-docked pose of a compound. Protein parameters were generated using the SwissParam server, and ligand parameters were generated using the TIP3P water model. The system was equilibrated at 1 bar pressure and 310 K temperature using NTP and NVT ensemble classes. Analysis of trajectories included root-mean-square deviation, RMSD, RMSF, Rg, SASA, and hydrogen bonds, with data visualized using XMGRACE software version 5.1.25.

## 4. Conclusions

A new series of benzimidazole derivative-containing chalcone units were synthesized in our present work using steps involving the key step of preparing benzimidazoles by an efficient ‘one-pot’ nitro reductive cyclization with good yield and purity. A single-crystal XRD study of compound **7c** revealed the crystallization in the triclinic crystal system with the space group P$\bar{1},$ and C-H..O and C-H…π are the major interactions responsible for its stability. The presence of C-H…O intermolecular hydrogen bonds in compound **7c** is confirmed by Hirshfeld surface analysis. FMO analysis shows an energy gap of 3.791 eV and 4.01 eV between HOMO and LUMO for compounds **7c** and **7l,** respectively, indicating that they are softer compounds. MEP analysis highlights the reactive sites and also supports structural details observed in XRD and the molecule’s reactivity. Out of twelve compounds, compound **7l** with a 3,4,5-trimethoxyphenyl ring on either side of the benzimidazole core demonstrated excellent α-glucosidase and α-amylase activity with IC_50_ values of 22.45 ± 0.36 µg/mL and 20.47 ± 0.60 µg/mL, respectively. Moreover, compound **7l** was found to be a safer candidate without exerting toxicity on the normal HEK293 cell line in comparison to standard Mitomycin. Additionally, molecular docking analysis was conducted on these compounds, and it revealed compound **7l** has the highest binding affinity towards both proteins through hydrogen bond and π-π interactions. DFT study of compound **7l** is in agreement with the observed docking results. Also, MD simulation studies of the compound **7l** revealed its stability within the inhibitor binding site of α-glucosidase and α-amylase proteins.

## Figures and Tables

**Table 1:** Hydrogen bond interactions of compound **7c**.

D-H…A	D-H (Å)	H…A (Å)	D-A (Å)	D-H…A (°)
O1S-H201…N1 ^1^	0.87 (4)	2.05	2.898 (3)	164 (4)
C7-H7…O1 *	0.93	2.31	2.689 (3)	104
C7-H7…O2 *	0.93	2.39	2.743 (3)	102
C15-H15…O2 *	0.93	2.43	2.749 (3)	100
C17-H17A…O1S ^2^	0.96	2.46	3.337 (3)	151

^1^ 1+x,y,z, ^2^ 1−x,−y,1−z, * intramolecular interaction.

**Table 2:** Molecular reactivity descriptors of compounds **7c** and **7l**.

Parameter	Energy	
	7c	7l
E_HOMO_ (eV)	−1.91	−2.02
E_LUMO_ (eV)	−5.70	−6.10
Energy gap (ΔE in eV)	3.79	4.08
Ionization potential (I in eV)	1.91	2.02
Electron affinity (E in eV)	5.70	6.10
Chemical potential (μ in eV)	−3.80	−4.06
Electronegativity (χ in eV)	3.80	4.06
Chemical hardness (η in eV)	1.89	2.04
Global softness (σ in eV^−1^)	0.528	0.487
Electrophilicity (ω in eV)	3.825	4.036

**Table 3:** α-glucosidase and α-amylase inhibitory activities of benzimidazole-endowed chalcone derivatives (**7a–l**).

Comp.	Structures	IC_50_ (µg/mL) (mean ± SD) ^x,y^	
		α-Glucosidase	α-Amylase
**7a**	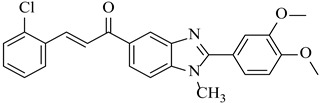	30.23 ± 0.14 h	40.15 ± 0.31 j
**7b**	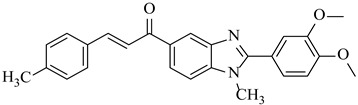	32.10 ± 0.96 i	33.42 ±0.23 e
**7c**	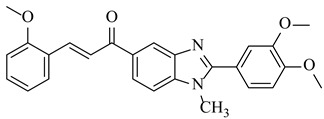	25.21 ± 0.13 c	39.38 ± 0.34 i
**7d**	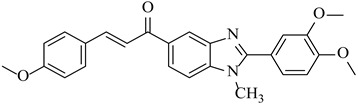	26.32 ± 0.52 d	36.41 ± 0.30 g
**7e**	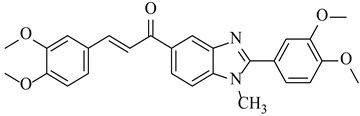	23.88 ± 0.55 b	34.42 ± 0.37 f
**7f**	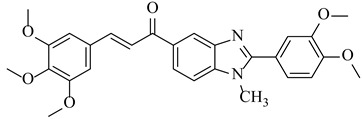	28.75 ± 0.12 f	37.34 ±0.37 h
**7g**	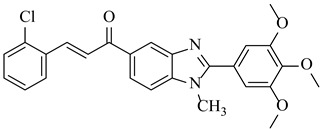	29.28 ± 0.35 g	28.58 ± 0.37 c
**7h**	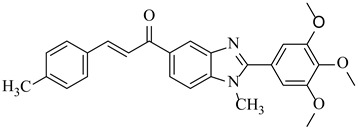	36.22 ± 0.58 l	30.48 ± 0.49 d
**7i**	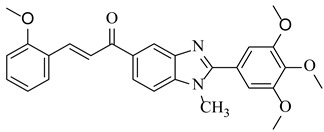	33.20 ± 0.60 j	24.21 ± 0.31 b
**7j**	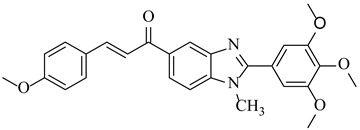	27.21 ± 0.36 e	39.27 ± 0.34 i
**7k**	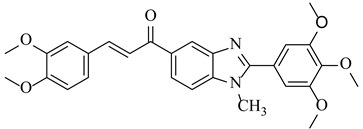	29.30 ± 0.21 g	41.25 ± 0.37 k
**7l**	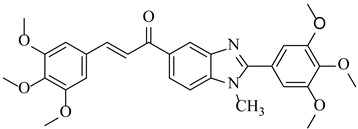	22.45 ± 0.36 a	20.47 ± 0.60 a
* Acarbose		35.05 ± 0.25 k	38.58 ± 0.32 j

^x^ Values are expressed as mean ± SE. Means in the same column with distinct superscripts are significantly different (*p* ≤ 0.05) as separated by Duncan multiple range test. ^y^ The IC_50_ value is defined as the inhibitor concentration to inhibit 50% of enzyme activity under assay conditions. * Standard: acarbose (positive control) for α-glucosidase and α-amylase.

**Table 4:** Binding affinity and interactions of compounds **7a–l** with α-glucosidase and α-amylase proteins.

Comp.	α-Glucosidase			α-Amylase		
	BA (kcal/mol)	TII	THB	BA (kcal/mol)	TII	THB
**7a**	−8.9	12	3	−8.9	12	2
**7b**	−8.7	11	2	−8.9	8	2
**7c**	−9.0	10	1	−8.7	13	1
**7d**	−9.4	9	2	−8.7	10	1
**7e**	−9.1	12	2	−8.5	12	1
**7f**	−8.7	14	7	−8.1	12	4
**7g**	−9.6	9	3	−8.7	13	2
**7h**	−9.6	12	3	−8.4	11	2
**7i**	−9.3	9	2	−8.2	8	2
**7j**	−9.2	9	3	−8.3	12	2
**7k**	−8.9	13	3	−8.1	12	2
**7l**	−10.0	23	8	−9.6	13	5
Acarbose	−7.8	8	8	−6.6	5	5

Note: TIM—total intermolecular interactions, THB—total hydrogen bonds, BA—binding affinity (kcal/mol).

**Table 5:** MD trajectory values of α-glucosidase complexed with compound **7l** and acarbose.

MD Trajectories	Apoprotein (α-Glucosidase)	α-Glucosidase–Compound 7l Complex	α-Glucosidase–Acarbose Complex
RMSD (nm)	0.30	0.25	0.30
RMSF (nm)	0.50	0.40	0.60
Rg (nm)	2.40	2.40	2.40
SASA (nm^2^)	225	220	230
Ligand H-bonds (max.)	-	7	2

**Table 6:** MD trajectory values of α-amylase complexed with compound **7l** and acarbose.

MD Trajectories	Apoprotein (α-Amylase)	α-Amylase–Compound 7l Complex	α-Amylase–Acarbose Complex
RMSD (nm)	0.30	0.30	0.35
RMSF (nm)	0.45	0.35	0.50
Rg (nm)	2.6	2.6	2.6
SASA (nm^2^)	245	240	245
Ligand H-bonds (max.)	-	7	2

## Data Availability

Data are contained within the article and Supplementary Materials.

## Supplementary Materials

The following supporting information can be downloaded at: https://www.mdpi.com/article/10.3390/molecules29235599/s1, Supplementary Materials contains spectral data of compounds **5b–f**, **7a–k**; the crystallographic data of compound **7c**; Figures S1–S24: ^1^H and ^13^C NMR spectra of **7a**, **7b**, **7c**, **7d**, **7e**, **7f**, **7g**, **7h**, **7i**, **7j**, **7k**, **7l**; Figures S25–S28: IR Spectra of **7d**, **7f**, **7h**, **7i**; Figures S29–S33: Mass Spectra of **7d**, **7f**, **7g**, **7h**, **7i**. Tables S1–S4: The crystallographic data of compound **7c**.
