# Supplementary material for: Novel Benzimidazole-Endowed Chalcones as α-Glucosidase and α-Amylase Inhibitors: An Insight into Structural and Computational Studies
Source: Molecules. 2024 Nov 27;29(23):5599. doi: 10.3390/molecules29235599 (PMC11643225; doi:10.3390/molecules29235599)
Supplement: Supplementary file 1 [file molecules-29-05599-s001.zip › molecules-3244995-supplementary.pdf]

Novel Benzimidazole Endowed Chalcones as α-Glucosidase and α-Amylase Inhibitors: An Insight into Structural and Computational Studies

Prashasthi V. Rai[1,†], Ramith Ramu[2,†], Akhileshwari P.[3], Sudharshan Prabhu[4], Nupura Manish Prabhune[4], Deepthi P.V.[5], Anjana P. T.[6], Ganavi D.[7], Vijesh A. M.[8], Khang Wen Goh[9], Mohammad Z. Ahmed[10], Vasantha Kumar[1,*]

Supplementary Information

1. Spectral data of compound **5b-f, 7a-k**

2. Table S1-S4: the crystallographic data of compound **7c**

3. Figure S1-S24: ^{1}H and ^{13}C NMR spectra of **7a, 7b, 7c, 7d, 7e, 7f, 7g, 7h, 7i, 7j,7k, 7l**; Figure S25-S28: IR Spectra of **7d, 7f, 7h, 7i**; Figure S29-S33: Mass Spectra of **7d, 7f, 7g, 7h, 7i**.

Spectral data for 5b-f:

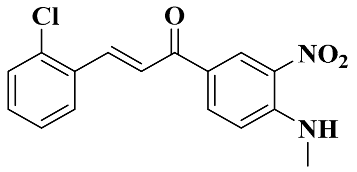

5b

(E)-3-(2-chlorophenyl)-1-(4-(methylamino)-3-nitrophenyl)prop-2-en-1-one ***(5b)***. Yield: 81-83%; M.p.: 170-172 °C; ^{1}H NMR (400 MHz, $CDCl_3$, δin ppm): 8.92 (d, 1H, J = 2Hz, Ar-H_2), 8.49 (d, 1H, J = 4.8 Hz, Ar-H), 8.24-8.20 (m, 2H, chalcone =CH, Ar-H), 7.81-7.78 (m, 1H, Ar-H), 7.53-7.44 (m, 2H, Ar-H and chalcone =CH), 7.36-7.33 (m, 2H, Ar-H), 3.14 (d, 3H, J = 5.2Hz, N-Me).

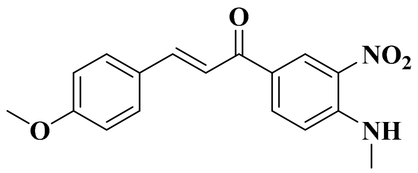

5c

(E)-3-(4-methoxyphenyl)-1-(4-(methylamino)-3-nitrophenyl)prop-2-en-1-one ***(5c)***. Yield: 84-86%; M.p.: 143-145 °C; ^{1}H NMR (400 MHz, $CDCl_3$, δin ppm): 8.91 (d, 1H, J = 2Hz, Ar-H), 8.45 (d, 1H, J = 4.8 Hz, Ar-H), 8.22 (dd, 1H, J = 9Hz and 1.8Hz, Ar-H), 7.82 (d, 1H, J = 15.2Hz, chalcone =CH), 7.63 (d, 2H, J= 8.4 Hz, Ar-H), 7.44 (d, 1H, J = 15.6Hz, chalcone =CH), 6.95 (d, 2H, J = 8.8Hz, Ar-H), 3.86 (s, 3H, -OMe), 3.12 (d, 3H, J = 5.2Hz, N-Me).

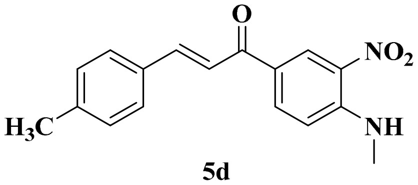

5d

2.3.4. (E)-1-(4-(methylamino)-3-nitrophenyl)-3-(p-tolyl)prop-2-en-1-one ***(5d)***. Yield: 83-86%; M.p.: 142-144 °C;^{1}H NMR (400 MHz, $CDCl_3$, δin ppm): 8.92 (d, 1H, J = 2Hz, Ar-H), 8.45 (d, 1H, J = 4.8 Hz, Ar-H), 8.23 (dd, 1H, J = 9.2Hz and 1.6Hz, Ar-H), 7.84 (d, 1H, J = 15.6Hz, chalcone =CH), 7.58-7.50 (m, 4H, Ar-H), 7.24 (d, 1H, Ar-H), 6.95 (d, 1H, J = 8.8Hz, Ar-H), 3.12 (s, 3H, N-Me), 2.40 (s, 3H, Me).

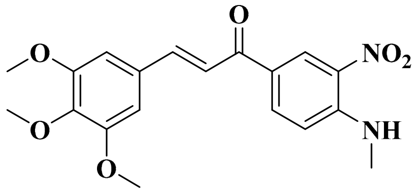

5e

(E)-1-(4-(methylamino)-3-nitrophenyl)-3-(3,4,5-trimethoxyphenyl)prop-2-en-1-one ***(5e)***. Yield: 87-89%; M.p.: 120-122 °C; ^{1}H NMR (400 MHz, $CDCl_3$, δin ppm): 8.92 (d, 1H, J = 2Hz, Ar-H), 8.44 (d, 1H, J = 4.8 Hz, Ar-H), 8.23 (dd, 1H, J = 9Hz and 1.8Hz, Ar-H), 7.83 (d, 1H, J = 15.2Hz, chalcone =CH), 7.45 (d, 1H, J = 15.6 Hz, chalcone =CH), 6.90 (s, 2H, J = 8.8Hz, Ar-H), 3.94 (s, 6H, OMe), 3.91 (s, 3H, OMe), 3.12 (d, 3H, J = 5.2Hz, N-Me).

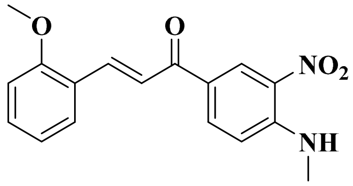

5f

(E)-3-(2-methoxyphenyl)-1-(4-(methylamino)-3-nitrophenyl)prop-2-en-1-one ***(5f)***. Yield: 88-90%; M.p.: 158-160 °C; ^{1}H NMR (400 MHz, $CDCl_3$, δin ppm): 8.91 (d, 1H, J = 2Hz, Ar-H), 8.43 (d, 1H, J = 4.8 Hz, Ar-H), 8.22 (dd, 1H, J = 9Hz and 1.8Hz, Ar-H), 7.82 (d, 1H, J = 6 Hz chalcone =CH), 7.59 (dd, 1H, J = 7.6 and 1.6 Hz, Ar-H), 7.45 (d, 1H, J = 6 Hz chalcone =CH), 7.35 (t, 1H, J = 8 Hz, Ar-H), 6.96 (d, 1H, J = 7.6 Hz, Ar-H), 6.90 (d, 1H, J = 8 Hz, Ar-H), 3.88 (s, 6H, OMe), 3.87 (s, 6H, OMe), 3.86 (s, 3H, N-Me).

Spectral data for 7a-k:

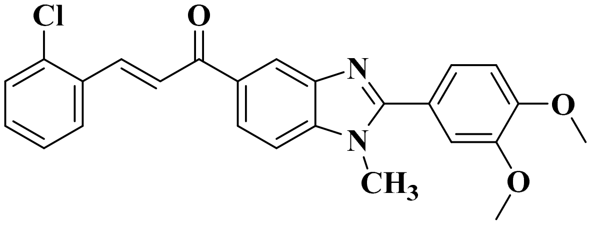

7a

(E)-3-(2-chlorophenyl)-1-(2-(3,4-dimethoxyphenyl)-1-methyl-1H-benzo[d]imidazol-5-yl)prop-2-en-1-one ***(7a)***. Yield: 73-75%; M.p.: 148-150 °C; FTIR (KBr, cm^{-1}): 3175 (=C-H), 2996 (-C=H), 2942 (asym C-H), 2836 (sym C-H), 1651 (C=O), 1587 (C=C and C=N), 1326 (C-O), 808 (C-Cl); ^{1}H NMR (400 MHz, DMSO, δin ppm): 8.66 (d, 1H, J = 1.2Hz, benzimidazole-H_4), 8.33-8.30 (m, 1H, benzimidazole-H_6), 8.18 (d, 1H, J = 15.6 Hz, chalcone =C-H), 8.11-8.04 (m, 2H, Ar-H_3 of 2-Cl-Ph and chalcone =C-H), 7.76 (d, 1H, J =8.4 Hz,

benzimidazole-H_7), 7.57 (d, 1H, J =8 Hz, Ar-H_6 of 2-Cl-Ph), 7.47-7.43 (m, 4H, Ar-$H_{2,6}$ of 3,4-$(OMe)_2$-Ph and Ar-$H_{4,5}$ of 2-Cl-Ph), 7.15 (d, 1H, J = 8.8 Hz, Ar-H_5 of 3,4-$(OMe)_2$-Ph), 3.94 (s, 3H, OMe), 3.85 (s, 3H, OMe), 3.84(s, 3H, N-CH_3); ^{13}C NMR (100 MHz, $CDCl_3$, δ in ppm): 196.5 (C=O), 155.3 (Ar-C-O), 150.3 (Ar-C-O), 148.7 (Ar-C-O), 142.3, 137.7, 134.3, 134.0, 132.9 132.4, 131.8, 131.7, 129.8, 128.8, 127.8, 125.1, 122.7, 122.3, 120.6, 112.5, 111.4, 110.7, 55.6 (O-CH_3), 55.46 (O-CH_3), 32.3 (N-CH_3); LCMS (m/z): 433.5 [M+1]; Anal. Calcd. for $C_{25}H_{21}ClN_2O_3$: C, 69.36; H, 4.89; Cl, 8.19; N, 6.47; O, 11.09; Found: C, 69.32; H, 4.87; Cl, 8.24; N, 6.53; O, 11.15.

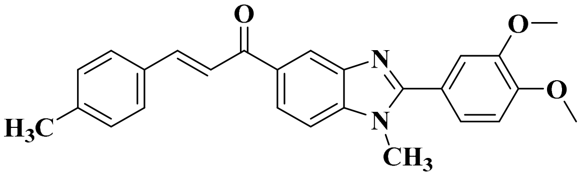

7b

(E)-1-(2-(3,4-dimethoxyphenyl)-1-methyl-1H-benzo[d]imidazol-5-yl)-3-(p-tolyl)prop-2-en-1-one ***(7b)***. Yield: 76-78%; M.p.: 160-162 ºC; FTIR (KBr, cm^{-1}): 3177 (=C-H), 2919 and 2844 (-C=H), 1656 (C=O), 1607 (C=N), 1523 (C=C);^{1}H NMR (400 MHz, DMSO, δin ppm): 8.62 (d, 1H, J = 0.8 Hz, benzimidazole-H_4), 8.10-8.05 (m, 2H, chalcone =CH and benzimidazole-H_6), 7.82 (d, 2H, J = 8 Hz, Ar-$H_{2,6}$-4-Me-Ph), 7.75-7.70 (m, 2H, benzimidazole-H_7 and chalcone =CH), 7.45-7.43 (m, 2H, Ar-$H_{2,6}$ of 3,4-$(OMe)_2$-Phenyl), 7.27 (d, 2H, J = 8 Hz, Ar-$H_{3,5}$-4-Me -Ph), 7.15 (d, 1H, J = 9.2 Hz, Ar-H_5 of 3,4-$(OMe)_2$-Phenyl)), 3.94 (s, 3H, OMe), 3.86 (s, 3H, OMe), 3.85 (s, 3H, OMe), 2.35 (s, 3H, N-CH_3); ^{13}C NMR (100 MHz, $CDCl_3$, δ in ppm): 188.4 (C=O), 169.6, 150.3 (Ar-C-O), 148.7 (Ar-C-O), 143.3 (=C), 142.1, 140.8, 140.4, 132.8, 132.1, 129.5, 128.8, 122.8, 122.1, 121.3, 120.4, 118.6, 112.6, 111.5, 110.6, 55.6 (O-CH_3), 55.38 (O-CH_3), 32.1 (N-CH_3), 21.1 (-CH_3); LCMS (m/z): 443.2[M+1]; Anal. Calcd. for $C_{26}H_{24}N_2O_3$: C, 75.71; H, 5.86; N, 6.79; O, 11.64; Found: C, 75.75; H, 5.83; N, 6.81; O, 11.60.

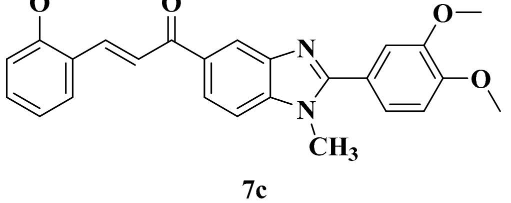

7c

(E)-1-(2-(3,4-dimethoxyphenyl)-1-methyl-1H-benzo[d]imidazol-5-yl)-3-(2-methoxyphenyl)prop-2-en-1-one ***(7c)***. Yield: 71-73%; M.p.: 159-161 °C; FTIR (KBr, cm^{-1}): 3363 (N-H), 2944 (asym C-H), 2838 (sym C-H), 1650 (C=O), 1581 (C=C); ^{1}H NMR (400 MHz, DMSO, δin ppm): 8.57 (d, 1H, J = 1.2 Hz, benzimidazole-H_4), 8.08-8.02 (m, 4H, chalcone =CH, Ar-$H_{4,6}$ of 2-OMe-Ph and benzimidazole-H_6), 7.74 (d, 1H, J = 8.8 Hz, benzimidazole-H_7), 7.46-7.422 (m, 3H, chalcone =CH and Ar-$H_{2,6}$ of 3,4-$(OMe)_2$-Phenyl), 7.16-7.10 (m, 2H, Ar-H_3 of 2-OMe-Ph and Ar-H_5 of 3,4-$(OMe)_2$-Phenyl), 7.03 (t, 1H, J = 7.4 Hz, Ar-H_5 of 2-OMe-Ph), 3.94 (s, 3H, OMe), 3.90 (s, 3H, OMe), 3.85 (s, 6H, OMe & N-CH_3); ^{13}C NMR (100 MHz, $CDCl_3$, δin ppm): 188.7 (C=O), 158.1 (Ar-C-O), 155.1, 150.3 (Ar-C-O), 148.7 (Ar-C-O), 142.1, 140.0, 137.6 (Ar-C-O), 132.2, 132.1, 129.1, 128.4, 123.1, 122.8, 122.1, 121.9, 120.7, 120.2, 112.7, 111.7, 111.5, 110.7, 55.7 (O-CH_3), 55.7 (O-CH_3), 55.6 (O-CH_3), 32.1 (N-CH_3); LCMS (m/z):429.2 [M+1]; Anal. Calcd. for $C_{26}H_{24}N_2O_4$: C, 72.88; H, 5.65; N, 6.54; O, 14.94; Found: C, 72.84; H, 5.64; N, 6.59; O, 14.90.

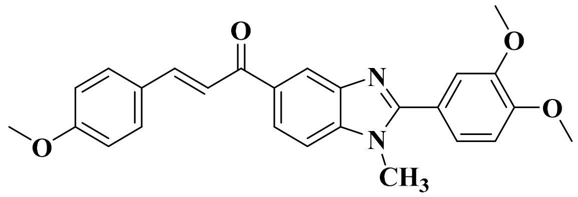

7d

(E)-1-(2-(3,4-dimethoxyphenyl)-1-methyl-1H-benzo[d]imidazol-5-yl)-3-(4-methoxyphenyl)prop-2-en-1-one ***(7d)***. Yield: 79-81%; M.p.: 98-100 °C; FTIR (KBr, cm^{-1}): 3176 (=C-H), 2943 (asym C-H), 2835 (sym C-H), 1649 (C=O); ^{1}H NMR (400 MHz, $CDCl_3$, δ in ppm): 8.50 (d, 1H, J = 1.2Hz, benzimidazole-H_4), 8.08 (dd, 1H, J = 8.4 & 1.6Hz, benzimidazole-H_6), 7.82 (d, 1H, J = 15.6 Hz, chalcone =CH), 7.62 (d, 2H, J = 8.8 Hz, Ar-$H_{3,5}$-4-OMe-Ph), 7.58 (d, 1H, J = 15.6 Hz, chalcone =CH), 7.46 (d, 1H, J = 8.4 Hz, benzimidazole-H_7), 7.40 (d, 1H, J = 2 Hz, Ar-H_2 of 3,4-$(OMe)_2$-Phenyl), 7.29 (dd, 1H, J = 8.4 Hz and 2 Hz, Ar-H_6 of 3,4-$(OMe)_2$-Phenyl), 7.01 (d, 1H, J = 8.4 Hz, Ar-H_5 of 3,4-$(OMe)_2$-Phenyl), 6.95 (d, 2H, J = 8.8 Hz, Ar-H-$_{2,6}$-4-OMe-Ph), 3.98 (s, 3H, OMe), 3.97 (s, 3H, OMe), 3.93 (s, 3H, OMe), 3.86 (s, 3H, *N*-CH_3); ^{13}C NMR (100 MHz, $CDCl_3$, δ in ppm): 190.0 (C=O), 161.5 (Ar-C-O), 155.5, 150.7 (Ar-C-O), 149.3 (Ar-C-O), 144.0(=C), 142.4, 139.7, 133.4, 130.1, 127.8, 123.5, 122.1, 122.0, 120.8, 120.0, 114.4, 112.6, 110.8, 109.7, 56.1 (O-CH3), 56.0 (O-CH_3), 55.4 (O-

CH_3), 32.1 (N-CH_3); LCMS (m/z): 429.0 [M+1]; Anal. Calcd. for $C_{26}H_{24}N_2O_4$: C, 72.88; H, 5.65; N, 6.54; O, 14.94; Found: C, 72.94; H, 5.69; N, 6.51; O, 14.98.

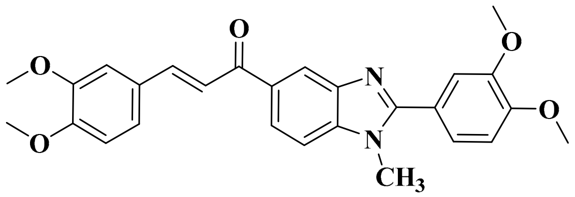

7e

(E)-3-(3,4-dimethoxyphenyl)-1-(2-(3,4-dimethoxyphenyl)-1-methyl-1H-benzo[d]imidazol-5-yl)prop-2-en-1-one ***(7e)***. Yield: 74-76%; M.p.: 142-144 °C; FTIR (KBr, cm^{-1}): 3176 (=C-H), 2993 and 2945 (asym C-H), 2835 (sym C-H), 1649 (C=O), 1583 (C=C and C=N), 1323 (O-CH_3); ^{1}H NMR (400 MHz, $CDCl_3$, δin ppm): 8.46 (d, 1H, J = 1.2Hz, benzimidazole-H_4), 8.03 (dd, 1H, J = 8.6 and 1.4 Hz, benzimidazole-H_6), 7.74 (d, 1H, J = 15.6 Hz, chalcone =CH), 7.51 (d, 1H, J = 15.6 Hz, chalcone =CH), 7.40 (d, 1H, J = 8.4 Hz, benzimidazole-H_7), 7.34 (d, 1H, J = 2 Hz, Ar-H_2 of 3,4-$(OMe)_2$-Ph), 7.22 (dd, 1H, J = 8.4 and 2 Hz, Ar-H_6 of 3,4-$(OMe)_2$-Ph), 7.18-7.14 (m, 2H, Ar-H_2 and Ar-H_6 of 3,4-$(OMe)_2$-Ph),), 6.95 (d, 1H, J = 8.4 Hz, Ar-H_5 of 3,4-$(OMe)_2$-Ph), 6.84 (d, 1H, J = 8.4 Hz, Ar-H_5 of 3,4-$(OMe)_2$-Ph), 3.92 (s, 3H, OMe), 3.91 (s, 6H, OMe), 3.87 (s, OCH_3), 3.86 (d, N-CH_3); ^{13}C NMR (100 MHz, $CDCl_3$, δ in ppm): 189.9 (C=O), 155.4 (Ar-C-O), 151.3 (Ar-C-O), 150.7 (Ar-C-O), 149.3 (Ar-C-O), 149.2 (Ar-C-O), 144.24 (=C), 142.4, 139.7, 133.2, 128.1, 123.5, 123.2, 122.1, 122.0, 120.8, 120.1, 112.6, 111.1, 110.8, 109.8, 109.7, 56.1 (O-CH_3), 56.0 (O-CH_3), 56.0 (O-CH_3), 55.9 (O-CH_3), 32.1 (N-CH_3); LCMS (m/z): 459.26 [M+1]; Anal. Calcd. for $C_{27}H_{26}N_2O_5$: C, 70.73; H, 5.72; N, 6.11; O, 17.45; Found: C, 70.69; H, 5.76; N, 6.08; O, 17.49.

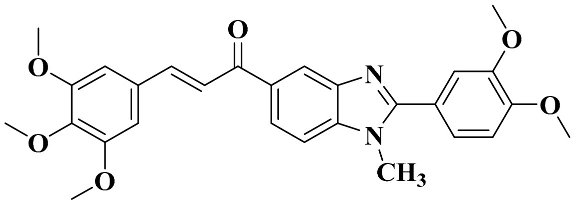

7f

(E)-1-(2-(3,4-dimethoxyphenyl)-1-methyl-1H-benzo[d]imidazol-5-yl)-3-(3,4,5-trimethoxyphenyl)prop-2-en-1-one ***(7f)****:* Yield: 77-79%; M.p.: 127-129 °C; FTIR (KBr, cm^{-1}): 3163 (=C-H), 3128 (=C-H), 2997 (=C-H), 2924 (=C-H), 1653 (C=O), 1608 (C=C and C=N),

1242 (O-CH_3); ^{1}H NMR (400 MHz, $CDCl_3$, δin ppm): 8.52 (s, 1H, benzimidazole-H_4), 8.09 (d, 1H, J = 8.4 Hz, benzimidazole-H_6), 7.76 (d, 1H, J = 15.2 Hz, chalcone =CH) 7.58 (d, 1H, J = 15.6 Hz, chalcone =CH), 7.46 (d, 1H, J = 8.4 Hz, benzimidazole-H_7), 7.39 (s, 1H, Ar-H_2 of 3,4-$(OMe)_2$-Ph), 7.29 (d, 1H, J = 8.8 Hz, Ar-H_6 of 3,4-$(OMe)_2$-Ph), 7.01 (d, 1H, J = 8.4 Hz, Ar-H_5 of 3,4-$(OMe)_2$-Ph), 6.88 (s, 2H, Ar-H-$_{2,6}$-3,4,5-$(OMe)_3$-Ph), 3.97 (s, 3H, OMe), 3.96 (s, 3H, OMe), 3.92 (s, 9H, OMe), 3.89 (s, 3H, N-CH_3); ^{13}C NMR (100 MHz, $CDCl_3$, δ in ppm): 189.7 (C=O), 155.6, 153.4 (Ar-C-O), 151.9, 150.8 (Ar-C-O), 149.3 (Ar-C-O), 144.2 (=C), 142.3, 140.2 (Ar-C-O), 139.8, 133.1, 130.6, 123.5, 122.0, 121.5, 120.9, 112.6, 110.8, 109.8, 105.4, 61.0, 56.2, 56.1, 56.0, 32.1; LCMS (m/z):489.2 [M+1]; Anal. Calcd. for $C_{28}H_{28}N_2O_6$: C, 68.84; H, 5.78; N, 5.73; O, 19.65; Found: C, 68.89; H, 5.73; N, 5.78; O, 19.60.

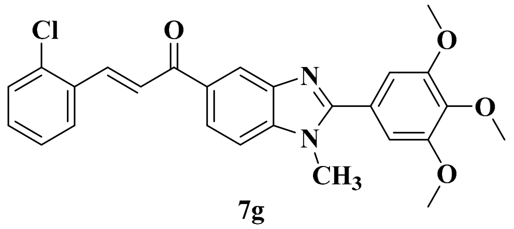

7g

(E)-3-(2-chlorophenyl)-1-(1-methyl-2-(3,4,5-trimethoxyphenyl)-1H-benzo[d]imidazol-5-yl)prop-2-en-1-one ***(7g)***. Yield: 75-77%; M.p.: 183-184 °C; FTIR (KBr, cm^{-1}): 3176 (=C-H), 2995 (-C=H), 2943 (asym C-H), 2835 (sym C-H), 1653 (C=O), 1585 (C=C and C=N), 1325 (C-O), 8086 (C-Cl); ^{1}H NMR (400 MHz, $CDCl_3$, δin ppm): 8.52 (d, 1H, J = 1.2Hz, benzimidazole-H_4), 8.23 (d, 1H, J = 16 Hz, chalcone =C-H), 8.11 (dd, 1H, J = 8.4 and 1.6 Hz, benzimidazole-H_6), 7.78 (d, 1H, J = 8 Hz, Ar-H_3 of 2-Cl-Ph), 7.66 (d, 1H, J =15.6 Hz, chalcone =C-H), 7.50-7.43 (m, 2H, benzimidazole-H_7 & Ar-H_6 of 2-Cl-Ph), 7.35-7.31 (m, 2H Ar-$H_{4,5}$ of 2-Cl-Ph), 6.99 (s, 2H, Ar-$H_{2,6}$ of 3,4,5-$(OMe)_3$-Phenyl), 3.95 (s, 9H, OMe), 3.94 (s, 1H, N-CH_3); ^{13}C NMR (100 MHz, $CDCl_3$, δ in ppm): 189.7 (C=O), 155.6, 153.4 (Ar-C-O), 142.3, 140.0, 139.8 (Ar-C-O), 136.4, 135.4, 133.4 132.9, 131.0, 130.2, 127.7, 127.0, 124.9, 124.7, 123.8, 121.2, 109.9, 106.8, 61.0 (O-CH_3), 56.4 (O-CH_3), 32.0 (N-CH_3); LCMS (m/z): 462.9[M+1]; Anal. Calcd. for $C_{26}H_{23}ClN_2O_4$: C, 67.46; H, 5.01; Cl, 7.66; N, 6.05; O, 13.82; Found: C, 67.42; H, 5.06; Cl, 7.61; N, 6.04; O, 13.88.

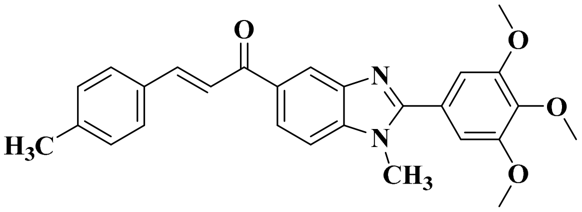

7h

(E)-1-(1-methyl-2-(3,4,5-trimethoxyphenyl)-1H-benzo[d]imidazol-5-yl)-3-(p-tolyl)prop-2-en-1-one ***(7h)***. Yield: 78-80%; M.p.: 168-170 °C; FTIR (KBr, cm^{-1}): 3176 (=C-H), 2920 and 2845 (-C=H), 1656 (C=O), 1608 (C=N), 1521 (C=C);

^{1}H NMR (400 MHz, $CDCl_3$, δin ppm): 8.52 (s, 1H, benzimidazole-H_4), 8.10 (d, 1H, J = 8.8 Hz, benzimidazole-H_6), 7.83 (d, 1H, J = 15.6 Hz, chalcone =CH), 7.65 (d, 1H, J = 15.6 Hz, chalcone =CH), 7.55 (d, 2H, J = 8 Hz, Ar-$H_{2,6}$-4-Me-Ph), 7.47 (d, 1H, J = 8.8 Hz, benzimidazole-H_7), 7.23 (d, 2H, J = 7.23 Hz, Ar-H-$_{3,5}$-4-Me-Ph), 6.99 (s, 2H, Ar-$H_{2,6}$-3,4,5-$(OMe)_3$-Ph), 3.95 (s, 6H, OMe), 3.94 (s, 6H, OMe & N-CH_3), 2.39 (s, 3H, Me); ^{13}C NMR (100 MHz, $CDCl_3$, δ in ppm): 190.0 (C=O), 155.5, 153.50 (Ar-C-O), 144.3, 142.3, 140.8 (Ar-C-O), 139.9, 139.6, 133.4, 132.3, 129.7, 128.3, 124.8, 123.7, 121.2, 121.1, 109.8, 106.9, 61.01 (O-CH_3), 56.4 (O-CH_3), 32.1 (N-CH_3), 21.51 (-CH_3); LCMS (m/z):443.0 [M+1]; Anal. Calcd. for $C_{27}H_{26}N_2O_4$: C, 73.28; H, 5.92; N, 6.33; O, 14.46; Found: C, 73.23; H, 5.98; N, 6.29; O, 14.51.

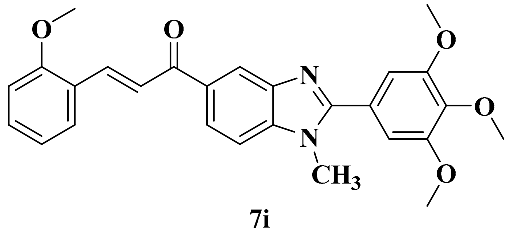

7i

(E)-3-(2-methoxyphenyl)-1-(1-methyl-2-(3,4,5-trimethoxyphenyl)-1H-benzo[d]imidazol-5-yl)prop-2-en-1-one ***(7i)***. Yield: 73-75%; M.p.: 176-178 °C; FTIR (KBr, cm^{-1}): 3364 (N-H), 2943 (asym C-H), 2837 (sym C-H), 1649 (C=O), 1583 (C=C); ^{1}H NMR (400 MHz, $CDCl_3$, δin ppm): 8.46 (d, 1H, benzimidazole-H_4), 8.09-8.03 (d, 1H, J = 14.4 Hz, chalcone =CH & benzimidazole-H_6), 7.75 (d, 1H, J = 16 Hz, chalcone =CH), 7.57 (dd, 1H, Ar-H_6 of 2-OMe-Ph), 7.40 (d, 1H, J = 8.4 Hz, benzimidazole-H_7), 7.33-7.29 (m, 1H, Ar-H_4 of 2-OMe-Ph), 6.94-6.88 (m, 4H, Ar-H_3-2-OMe-Ph, Ar-H_5 of 2-OMe-Ph and Ar-$H_{2,6}$ of 3,4,5-$(OMe)_3$-Phenyl),3.88 (s, 6H, OMe), 3.87 (s, 3H, OMe), 3.86 (s, 6H, OMe & N-CH_3); ^{13}C NMR (100 MHz, $CDCl_3$, δ in ppm): 190.5 (C=O), 158.8 (Ar-C-O), 155.4, 153.5 (Ar-C-O), 142.3, 140.4, 139.9 (Ar-C-

O), 139.69 (Ar-C-O), 133.6, 131.5, 129.5, 124.9, 124.1, 123.8, 123.1, 121.1, 120.7, 111.2, 109.7, 106.9, 61.0 (O-CH_3), 56.4 (O-CH_3), 55.5 (O-CH_3), 32.0 (N-CH_3);LCMS (m/z):459.0 [M+1]; Anal. Calcd. for $C_{27}H_{26}N_2O_5$: C, 70.73; H, 5.72; N, 6.11; O, 17.45; Found: C, 70.79; H, 5.69; N, 6.16; O, 17.41.

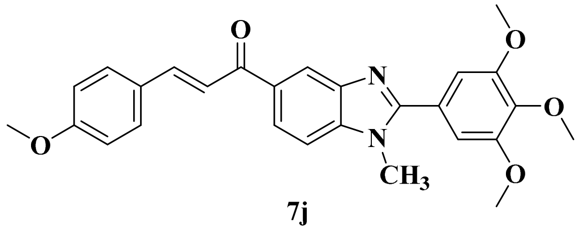

7j

*(E)-3-(4-methoxyphenyl)-1-(1-methyl-2-(3,4,5-trimethoxyphenyl)-1H-benzo[d]imidazol-5-yl)prop-2-en-1-one **(7j)***. Yield: 76-78%; M.p.: 142-144 °C; FTIR (KBr, cm^{-1}): 3175 (=C-H), 2942 (asym C-H), 2836 (sym C-H), 1648 (C=O);

^{1}H NMR (400 MHz, DMSO, δ in ppm): 8.57 (s, 1H, benzimidazole-H_4), 8.17 (d, 1H, J = 8.4 Hz, benzimidazole-H_6), 7.94 (d, 1H, J = 15.6 Hz, chalcone =CH), 7.89-7.85 (m, 2H, J = 8 Hz, Ar-$H_{2,6}$-4-OMe-Ph and benzimidazole-H_7), 7.71 (d, 1H, J = 15.6 Hz, chalcone =CH), 7.16 (s, 2H, Ar-$H_{2,6}$ of 3,4,5-$(OMe)_3$-Ph)), 6.98 (d, 2H, J = 8.8 Hz Ar-$H_{3,5}$-4-OMe-Ph), 3.97 (s, 3H, OMe), 3.84 (s, 6H, OMe), 3.78 (s, 3H, OMe), 3.73 (s, 3H, N-CH_3); ^{13}C NMR (100 MHz, DMSO, δ in ppm): 188.1 (C=O), 161.4 (Ar-C-O), 153.7, 153.1 (Ar-C-O), 144.0, 139.9, 138.1, 133.9, 130.9, 127.4, 124.3, 119.6, 118.0, 120.0, 114.4, 111.9 107.4, 60.2 (O-CH_3), 56.3 (O-CH_3), 55.4 (O-CH_3), 32.6 (N-CH_3); LCMS (m/z): 459.3[M+1]; Anal. Calcd. for $C_{27}H_{26}N_2O_5$: C, 70.73; H, 5.72; N, 6.11; O, 17.45; Found: C, 72.90; C, 70.77; H, 5.76; N, 6.08; O, 17.49.

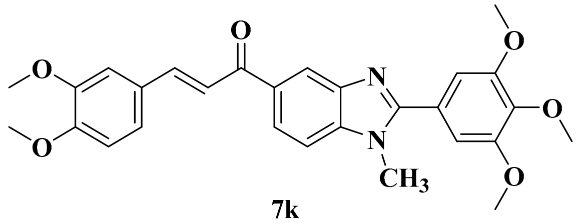

7k

*(E)-3-(3,4-dimethoxyphenyl)-1-(1-methyl-2-(3,4,5-trimethoxyphenyl)-1H-benzo[d]imidazol-5-yl)prop-2-en-1-one **(7k)***. Yield: 76-79%; M.p.: 137-139 °C; FTIR (KBr, cm^{-1}): 3175 (=C-H), 2995 and 2946 (asym C-H), 2836 (sym C-H), 1648 (C=O), 1582 (C=C and C=N), 1325 (O-CH_3);^{1}H NMR (400 MHz, DMSO, δin ppm): 8.61 (s, 1H, benzimidazole-H_4), 8.22 (d, 1H, J = 8.4 Hz, benzimidazole-H_6), 7.98-7.94 (m, 2H, benzimidazole-H_7 and chalcone =CH), 7.72 (d,

1H, *J* = 15.6 Hz, chalcone =CH), 7.56 (d, 1H, *J* = 2 Hz, Ar-H_2 of 3,4-(OMe)2-Ph), 7.38-7.36 (dd, 1H, *J* = 8.4 Hz and 2 Hz, Ar-H_6 of 3,4-$(OMe)_2$-Ph), 7.19 (s, 2H, Ar-$H_{2,6}$-3,4,5-$(OMe)_3$-Ph), 6.99 (d, 1H, *J* = 8.4 Hz, Ar-H_5-3,4-$(OMe)_2$-Ph), 4.00 (s, 3H, OMe), 3.85 (s, 6H, OMe), 3.84 (s, 3H, OMe), 3.78 (s, 3H, OMe), 3.74 (s, 3H, N-CH_3); ^{13}C NMR (100 MHz, $CDCl_3$, δ in ppm): 188.0 (C=O), 153.1 (Ar-C-O), 151.4 (Ar-C-O), 149.0 (Ar-C-O), 144.7, 140.3, 137.5, 135.3 (Ar-C-O), 127.5, 124.2, 120.3, 119.5, 117.3, 112.4, 111.5, 110.6, 107.6, 60.3 (O-CH_3), 56.3 (O-CH_3), 55.8 (O-CH_3), 55.6 (O-CH_3), 32.7 (N-CH_3); LCMS (m/z): 489.4[M+1]; Anal. Calcd. for $C_{28}H_{28}N_2O_6$: C, 68.84; H, 5.78; N, 5.73; O, 19.65; Found: C, 68.80; H, 5.74; N, 5.77; O, 19.61.

Table S1. Crystal data and refinement details of compound **7c**.

Empirical formula	$C_{26}H_{26}N_2O_5$
Formula weight	446.49
Temperature	296 K
Wavelength	0.71073 Å
Reflns. for cell determination	3780
θ range for above	4.69° to 49.75°
Crystal system	Triclinic
Space group	$P\bar{1}$
Cell dimensions	*a* = 9.7376(15) Å, *b* = 11.374(2) Å, *c* = 11.481(2) Å, α = 79.140(9)° β = 85.345(9)°, γ = 65.010(8)°
Volume	1131.9(3) Å^3
Z	2

Density(calculated)	1.310 Mg m^{-3}
Absorption coefficient	0.091 mm^{-1}
F_{000}	472
Crystal size	0.200 mm × 0.250 mm × 0.300 mm
θ range for data collection	1.81° to 28.65°
Index ranges	$-11 \leq h \leq 12$
	$-15 \leq k \leq 14$
	$-15 \leq l \leq 15$
Reflections collected	13981
Independent reflections	5548 [R_{int} = 0.034]
Refinement method	Full matrix least-squares on F^2
Data / restraints / parameters	5548 / 3 / 310
Goodness-of-fit on F^2	0.949
Final R indices [$I > 2\sigma(I)$]	$R1$ = 0.0525, $wR2$ = 0.1420
R indices (all data)	$R1$ = 0.1006, $wR2$ = 0.1855
Largest diff. peak and hole	0.223; -0.300 e $Å^{-3}$

Table S2. Bond lengths of non-hydrogen atoms of compound **7c**.

Atoms	Length (Å)	Atoms	Length (Å)
O1-C5	1.358(3)	C6-C7	1.445(3)
O1-C16	1.416(4)	C7-C8	1.309(3)
O2-C9	1.220(3)	C8-C9	1.467(3)
O3-C23	1.359(3)	C9-C10	1.486(3)
O3-C25	1.421(3)	C10-C11	1.385(3)
O4-C22	1.361(3)	C10-C15	1.404(3)
O4-C26	1.414(4)	C11-C12	1.379(3)
N1-C12	1.383(3)	C12-C13	1.395(3)
N1-C18	1.312(3)	C13-C14	1.388(3)
N2-C13	1.363(3)	C14-C15	1.366(3)
N2-C17	1.451(3)	C18-C19	1.459(3)
N2-C18	1.375(3)	C19-C20	1.379(3)
C1-C2	1.363(4)	C19-C24	1.398(3)
C1-C6	1.387(4)	C20-C21	1.380(3)
C2-C3	1.371(5)	C21-C22	1.374(3)
C3-C4	1.375(5)	C22-C23	1.399(3)
C4-C5	1.372(4)	C23-C24	1.368(3)
C5-C6	1.401(3)		

Table S3. Bond angles of non-hydrogen atoms compound **7c**.

Atoms	Angle (°)	Atoms	Angle (°)
C5-O1-C16	119.0(2)	C10-C11-C12	118.8(2)
C23-O3-C25	116.8(2)	N1-C12-C11	130.5(2)
C22-O4-C26	118.1(2)	N1-C12-C13	109.5(2)
C12-N1-C18	105.3(2)	C11-C12-C13	120.0(2)
C13-N2-C17	124.4(2)	N2-C13-C12	106.1(2)
C13-N2-C18	106.6(2)	N2-C13-C14	132.0(2)
C17-N2-C18	129.0(2)	C12-C13-C14	122.0(2)
C2-C1-C6	122.0(3)	C13-C14-C15	117.2(2)
C1-C2-C3	118.9(3)	C10-C15-C14	121.9(2)
C2-C3-C4	121.2(3)	N1-C18-N2	112.5(2)
C3-C4-C5	119.7(3)	N1-C18-C19	124.2(2)
O1-C5-C4	124.1(2)	N2-C18-C19	123.2(2)
O1-C5-C6	115.5(2)	C18-C19-C20	122.5(2)
C4-C5-C6	120.4(2)	C18-C19-C24	118.5(2)
C1-C6-C5	117.9(2)	C20-C19-C24	119.0(2)
C1-C6-C7	123.1(2)	C19-C20-C21	120.4(2)
C5-C6-C7	119.1(2)	C20-C21-C22	120.5(2)
C6-C7-C8	128.2(2)	O4-C22-C21	124.9(2)
C7-C8-C9	121.1(2)	O4-C22-C23	115.5(2)
O2-C9-C8	119.4(2)	C21-C22-C23	119.6(2)
O2-C9-C10	120.4(2)	O3-C23-C22	115.1(2)
C8-C9-C10	120.2(2)	O3-C23-C24	125.2(2)

**Atoms**	**Angle (°)**	**Atoms**	**Angle (°)**
C9-C10-C11	122.8(2)	C22-C23-C24	119.6(2)
C9-C10-C15	117.1(2)	C19-C24-C23	120.8(2)
C11-C10-C15	120.1(2)		

Table S4. Torsion angles of non-hydrogen atoms compound **7c**.

Atoms	Angle (°)	Atoms	Angle (°)
C16-O1-C5-C4	4.7(4)	O2-C9-C10-C15	2.4(3)
C16-O1-C5-C6	-174.9(2)	C8-C9-C10-C15	-177.2(2)
C25-O3-C23-C24	1.9(3)	C8-C9-C10-C11	3.6(3)
C25-O3-C23-C22	-179.5(2)	O2-C9-C10-C11	-176.8(2)
C26-O4-C22-C23	-179.4(2)	C11-C10-C15-C14	0.9(3)
C26-O4-C22-C21	0.6(3)	C9-C10-C15-C14	-178.4(2)
C12-N1-C18-C19	178.3(2)	C15-C10-C11-C12	-0.2(3)
C12-N1-C18-N2	-0.1(2)	C9-C10-C11-C12	178.9(2)
C18-N1-C12-C13	-0.3(2)	C10-C11-C12-C13	-0.5(3)
C18-N1-C12-C11	179.3(2)	C10-C11-C12-N1	179.9(2)
C17-N2-C13-C12	-178.5(2)	C11-C12-C13-C14	0.7(3)
C18-N2-C13-C12	-0.7(2)	N1-C12-C13-C14	-179.7(2)
C13-N2-C18-C19	-177.9(2)	C11-C12-C13-N2	-179.0(2)
C18-N2-C13-C14	179.6(2)	N1-C12-C13-N2	0.6(2)
C17-N2-C18-C19	-0.3(3)	C12-C13-C14-C15	-0.1(3)
C17-N2-C13-C14	1.8(3)	N2-C13-C14-C15	179.5(2)
C13-N2-C18-N1	0.5(2)	C13-C14-C15-C10	-0.7(3)
C17-N2-C18-N1	178.2(2)	N1-C18-C19-C20	135.9(2)
C2-C1-C6-C7	178.3(2)	N1-C18-C19-C24	-43.8(3)
C6-C1-C2-C3	0.4(4)	N2-C18-C19-C20	-45.8(3)
C2-C1-C6-C5	-0.9(3)	N2-C18-C19-C24	134.5(2)
C1-C2-C3-C4	-0.3(5)	C18-C19-C24-C23	-178.2(2)

Atoms	Angle (°)	Atoms	Angle (°)
C2-C3-C4-C5	0.9(5)	C24-C19-C20-C21	0.0(3)
C3-C4-C5-C6	-1.4(4)	C20-C19-C24-C23	2.1(3)
C3-C4-C5-O1	179.1(2)	C18-C19-C20-C21	-179.7(2)
O1-C5-C6-C1	-179.1(2)	C19-C20-C21-C22	-2.1(3)
O1-C5-C6-C7	1.7(3)	C20-C21-C22-C23	2.2(3)
C4-C5-C6-C7	-177.8(2)	C20-C21-C22-O4	-177.8(2)
C4-C5-C6-C1	1.4(3)	O4-C22-C23-O3	1.2(3)
C1-C6-C7-C8	-8.8(4)	C21-C22-C23-C24	-0.1(3)
C5-C6-C7-C8	170.4(2)	O4-C22-C23-C24	179.9(2)
C6-C7-C8-C9	-178.9(2)	C21-C22-C23-O3	-178.7(2)
C7-C8-C9-O2	3.7(3)	O3-C23-C24-C19	176.4(2)
C7-C8-C9-C10	-176.8(2)	C22-C23-C24-C19	-2.0(3)

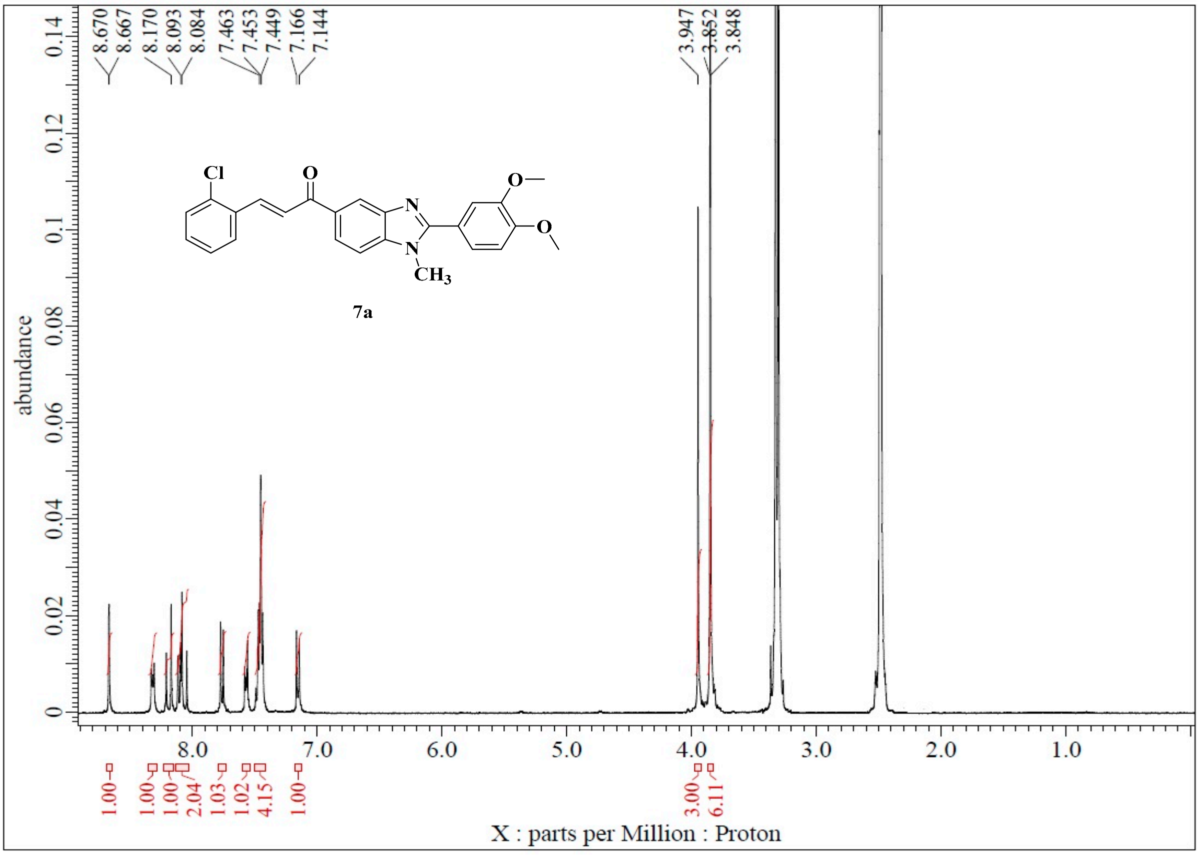

Figure S1. ^{1}H NMR spectrum of (E)-3-(2-chlorophenyl)-1-(2-(3,4-dimethoxyphenyl)-1-methyl-1H-benzo[d]imidazol-5-yl)prop-2-en-1-one **(7a)**

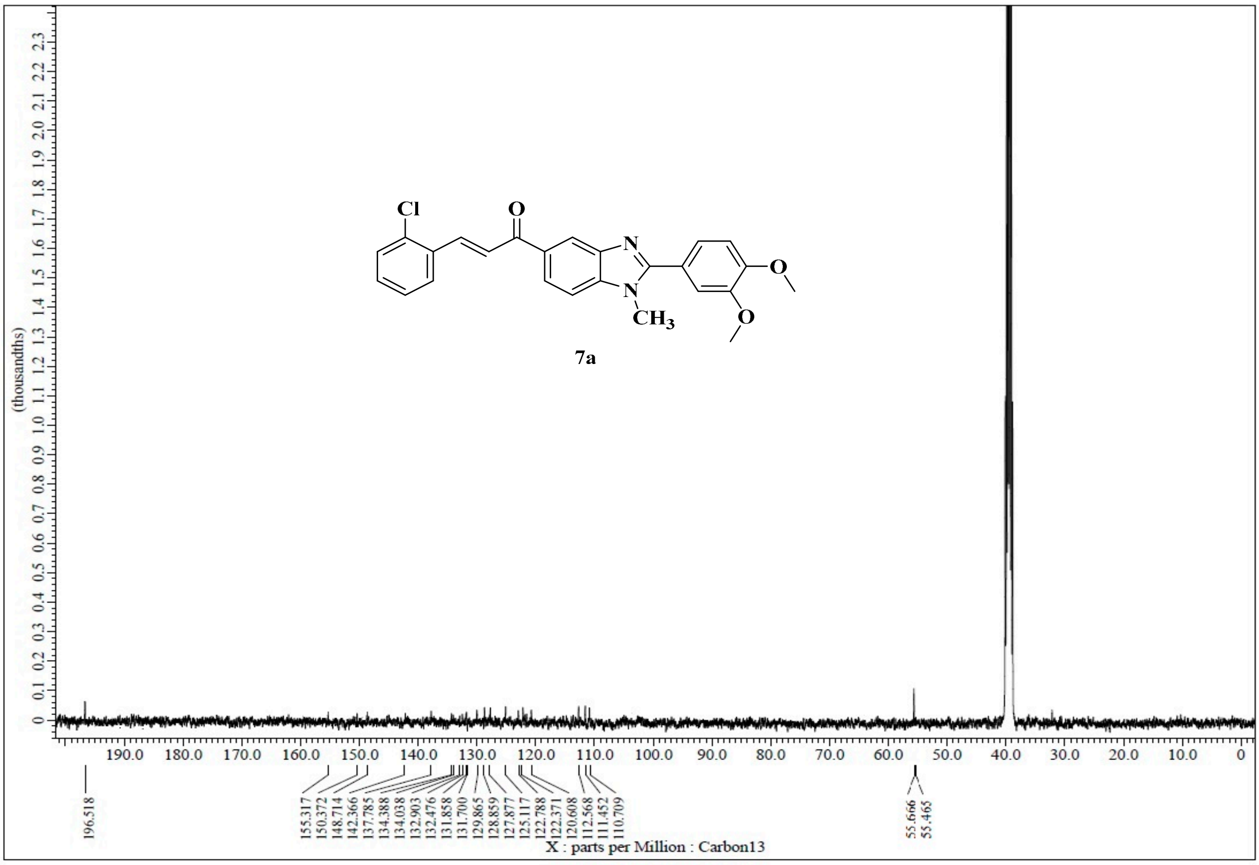

Figure S2. ^{13}C NMR spectrum of (E)-3-(2-chlorophenyl)-1-(2-(3,4-dimethoxyphenyl)-1-methyl-1H-benzo[d]imidazol-5-yl)prop-2-en-1-one **(7a)**

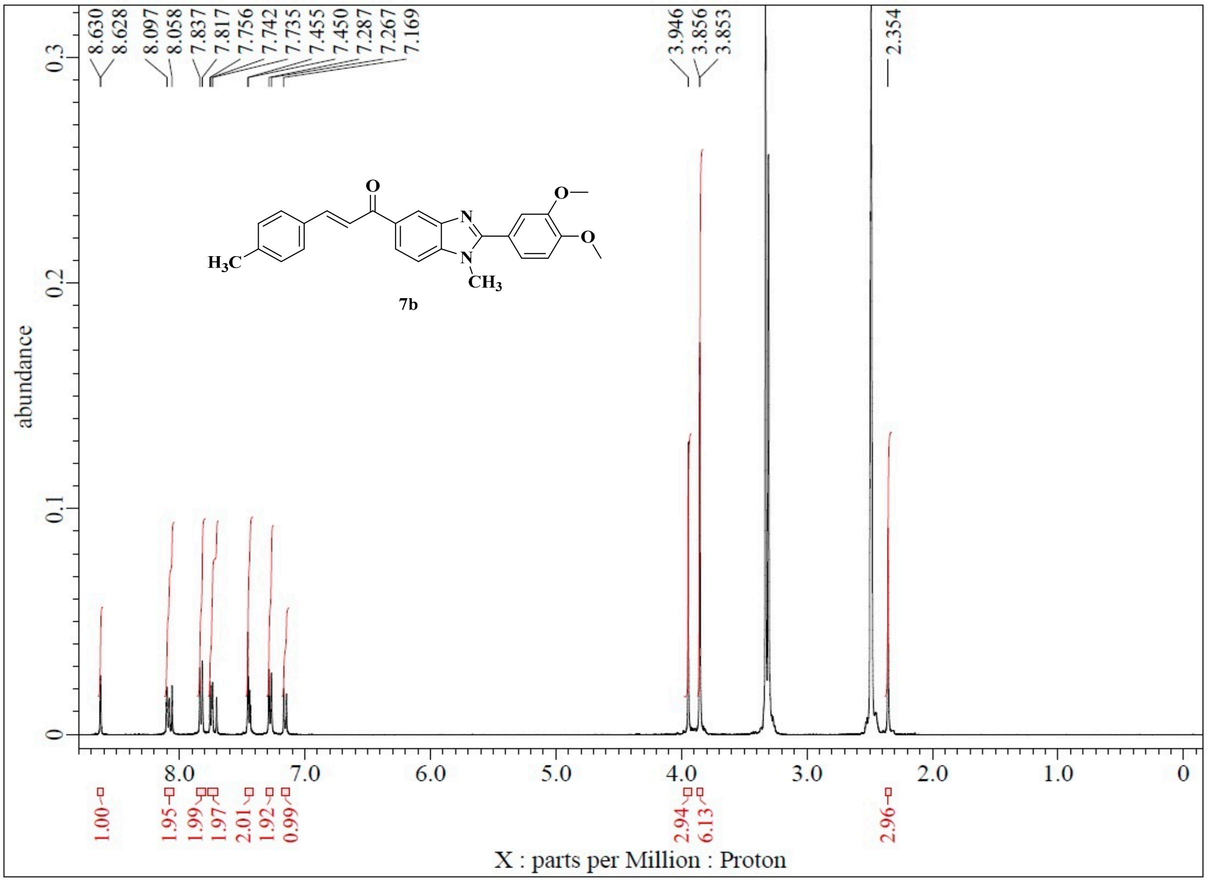

Figure S3. ^{1}H NMR spectrum of (E)-1-(2-(3,4-dimethoxyphenyl)-1-methyl-1H-benzo[d]imidazol-5-yl)-3-(p-tolyl)prop-2-en-1-one **(7b)**

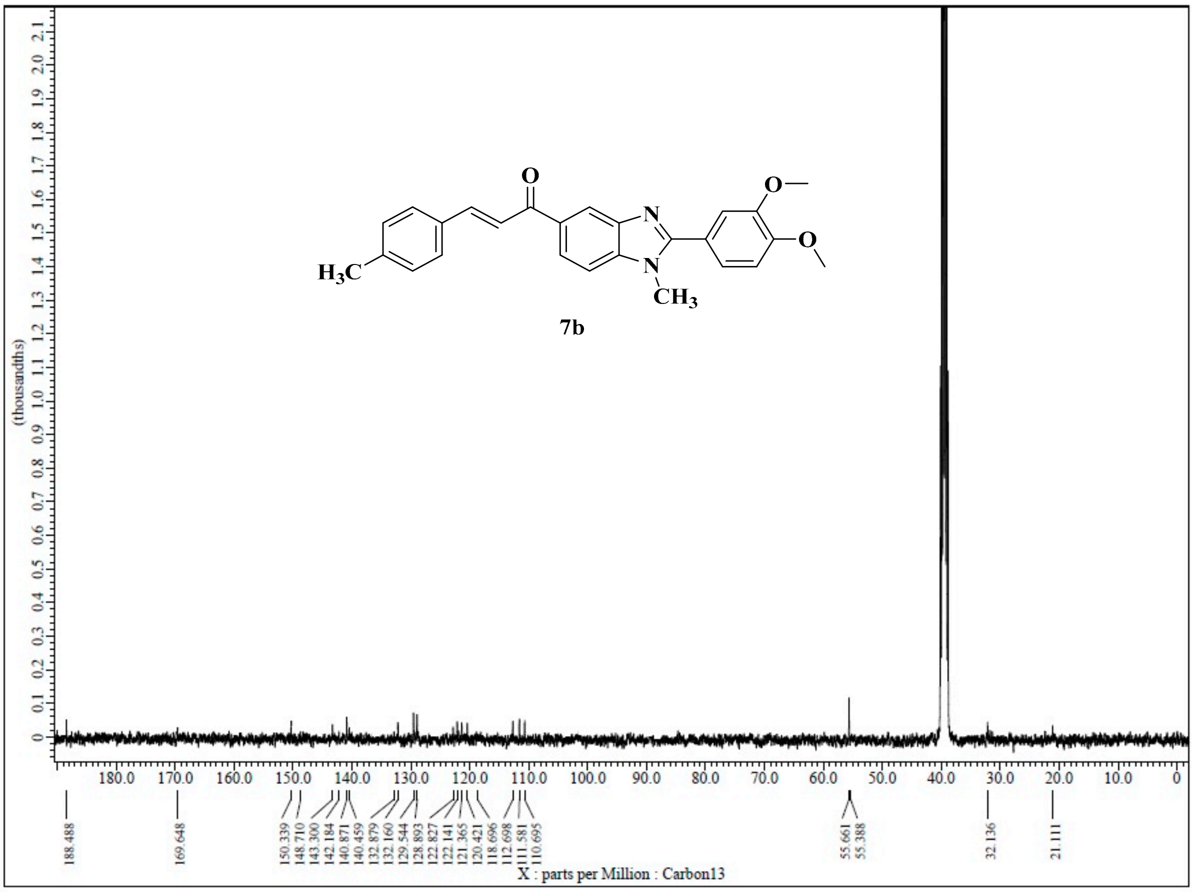

Figure S4. ^{13}C NMR spectrum of (E)-1-(2-(3,4-dimethoxyphenyl)-1-methyl-1H-benzo[d]imidazol-5-yl)-3-(p-tolyl)prop-2-en-1-one **(7b)**

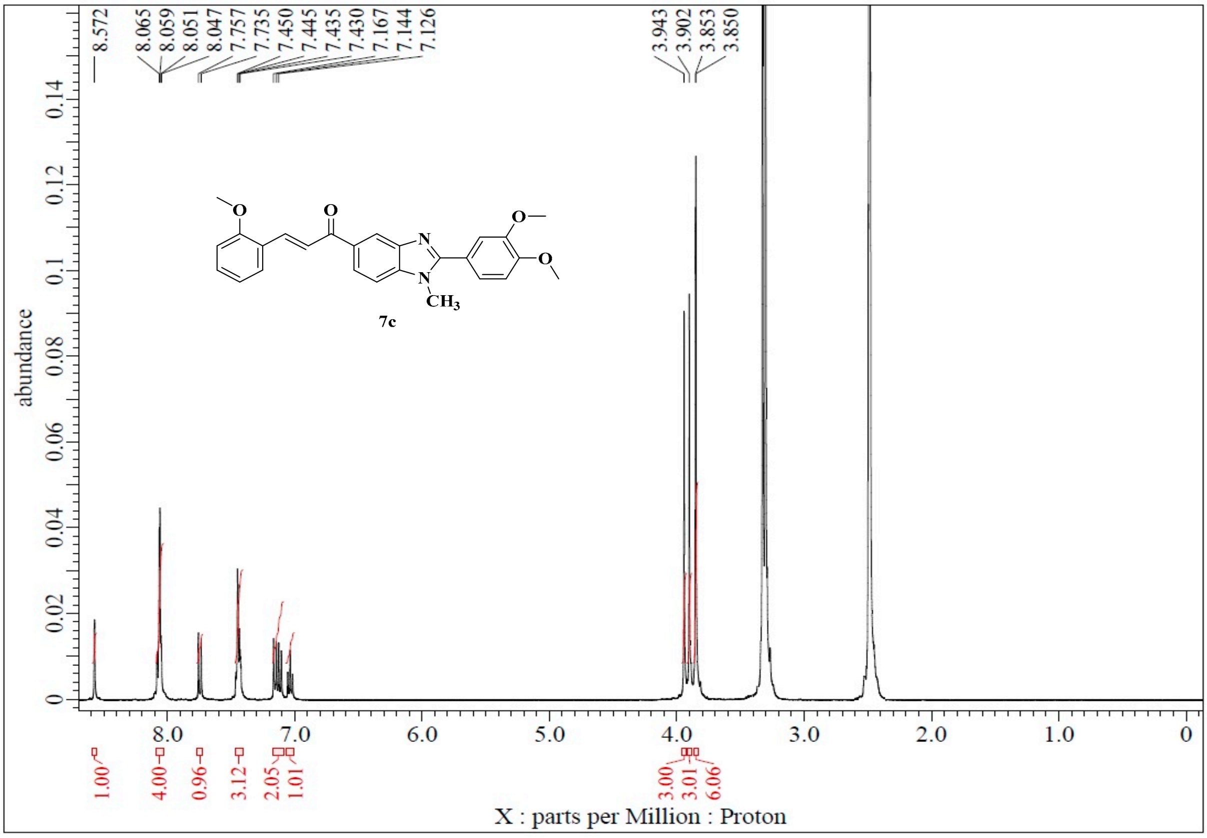

Figure S5. ^{1}H NMR spectrum of (E)-1-(2-(3,4-dimethoxyphenyl)-1-methyl-1Hbenzo[d]imidazol-5-yl)-3-(2-methoxyphenyl)prop-2-en-1-one **(7c)**

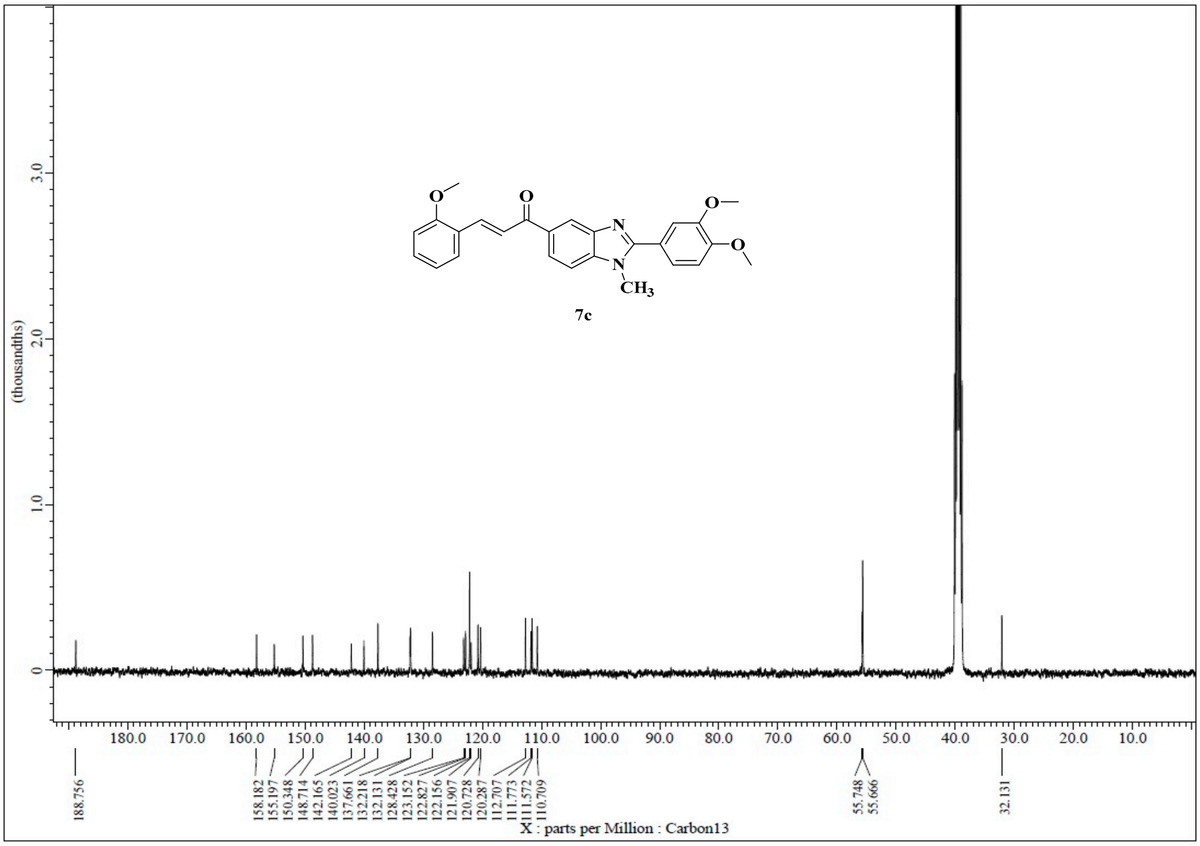

Figure S6. ^{13}C NMR spectrum of (E)-1-(2-(3,4-dimethoxyphenyl)-1-methyl-1H-benzo[d]imidazol-5-yl)-3-(2-methoxyphenyl)prop-2-en-1-one **(7c)**

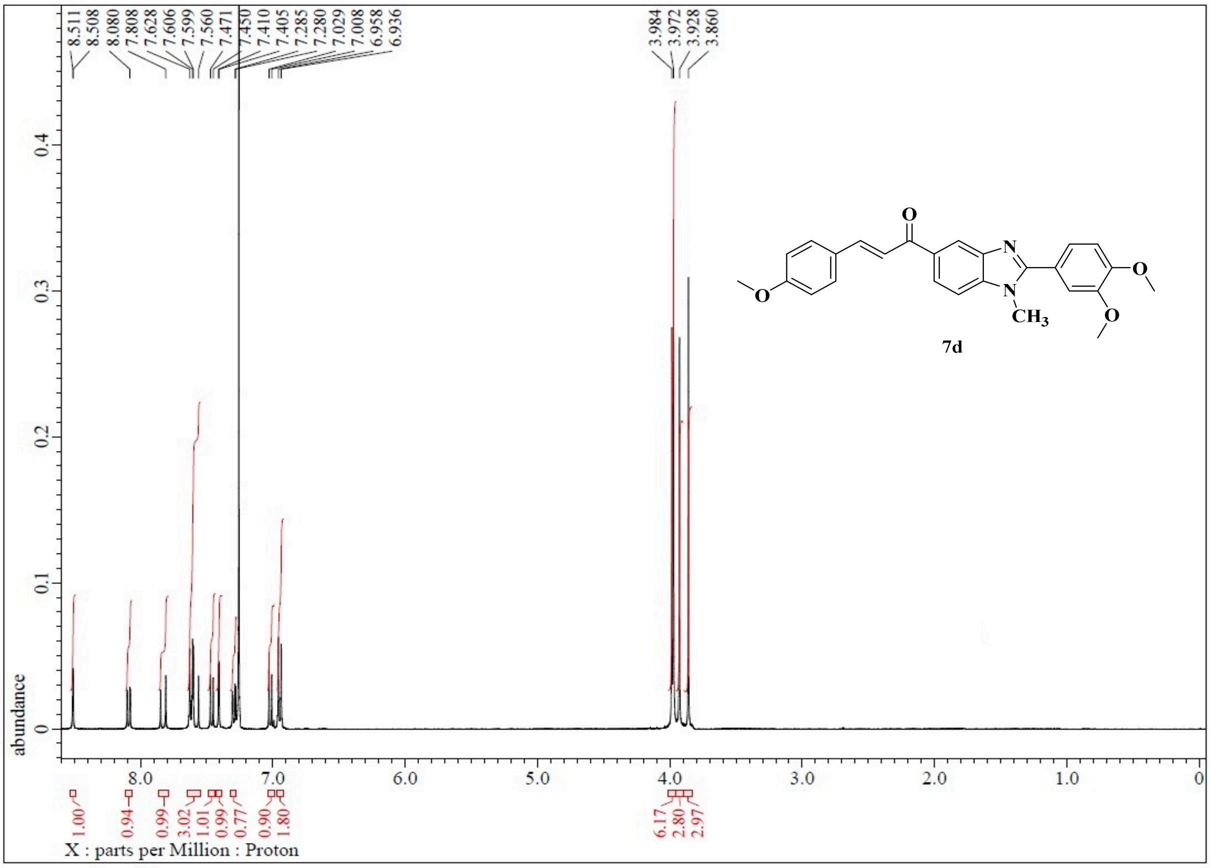

Figure S7. ^{1}H NMR spectrum of *(E)*-1-(2-(3,4-dimethoxyphenyl)-1-methyl-*1H*-benzo[*d*]imidazol-5-*yl*)-3-(4-methoxyphenyl)prop-2-en-1-one **(7d)**

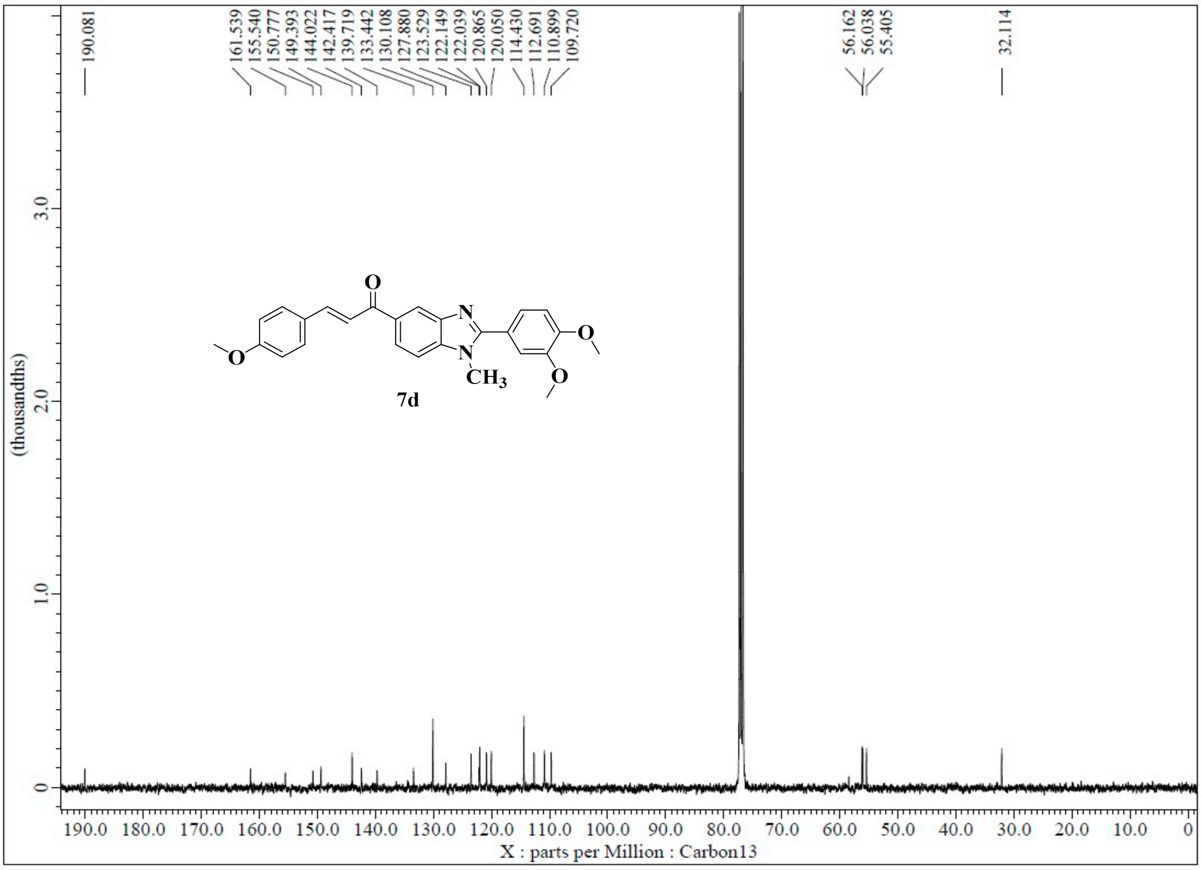

Figure S8. ^{13}C NMR spectrum of *(E)*-1-(2-(3,4-dimethoxyphenyl)-1-methyl-*1H*-benzo[*d*]imidazol-5-*yl*)-3-(4-methoxyphenyl)prop-2-en-1-one **(7d)**

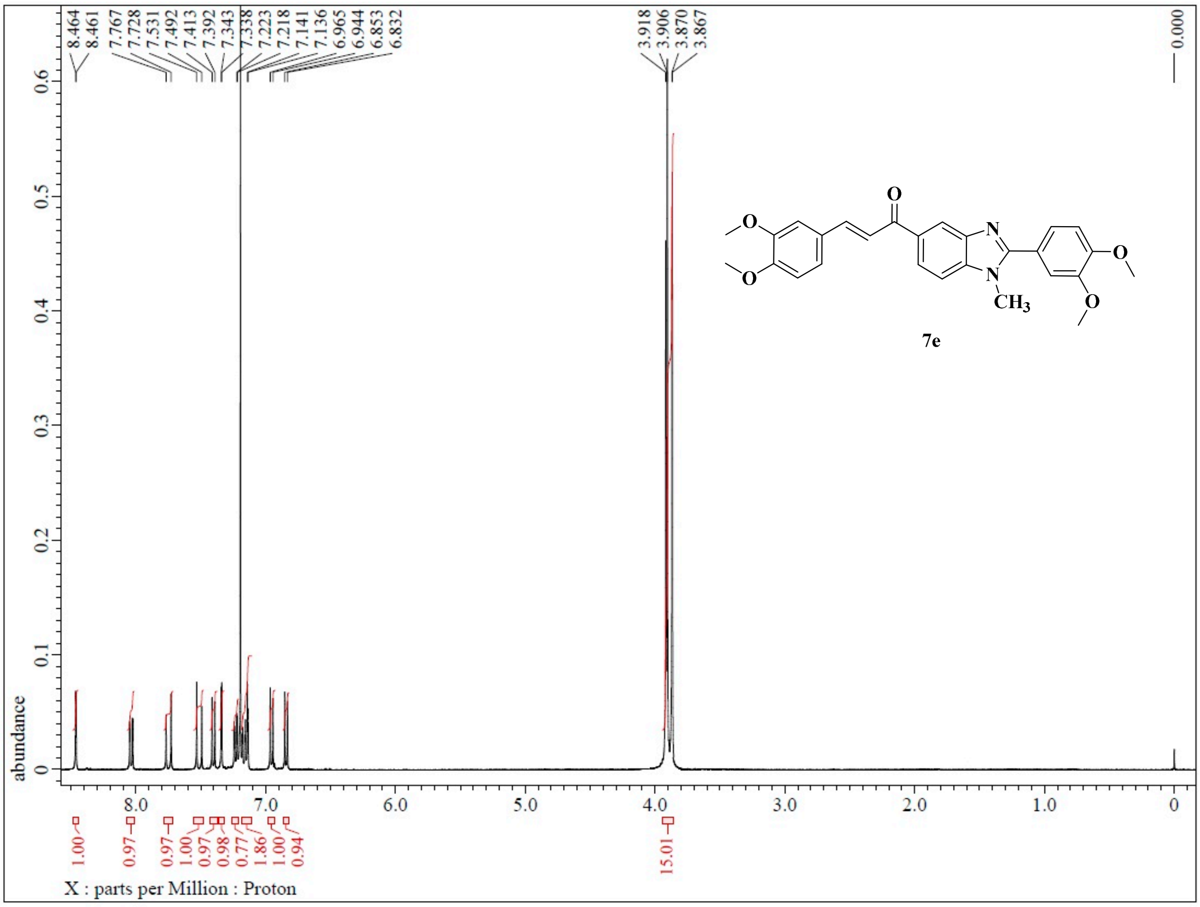

Figure S9. ^{1}H NMR spectrum of *(E)*-3-(3,4-dimethoxyphenyl)-1-(2-(3,4-dimethoxyphenyl)-1-methyl-*1H*-benzo[*d*]imidazol-5-*yl*)prop-2-en-1-one **(7e)**

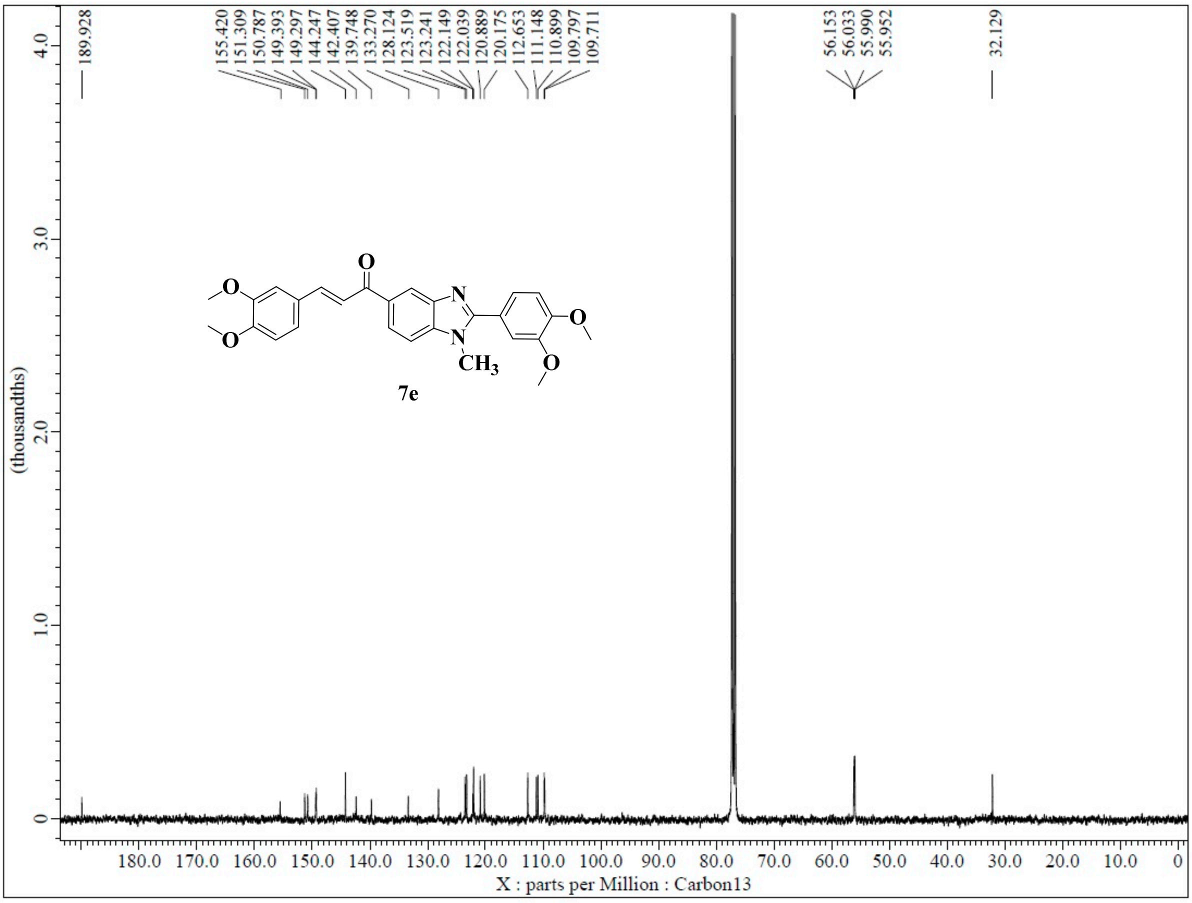

Figure S10. ^{13}C NMR spectrum of *(E)*-3-(3,4-dimethoxyphenyl)-1-(2-(3,4-dimethoxyphenyl)-1-methyl-*1H*-benzo[*d*]imidazol-5-*yl*)prop-2-en-1-one **(7e)**

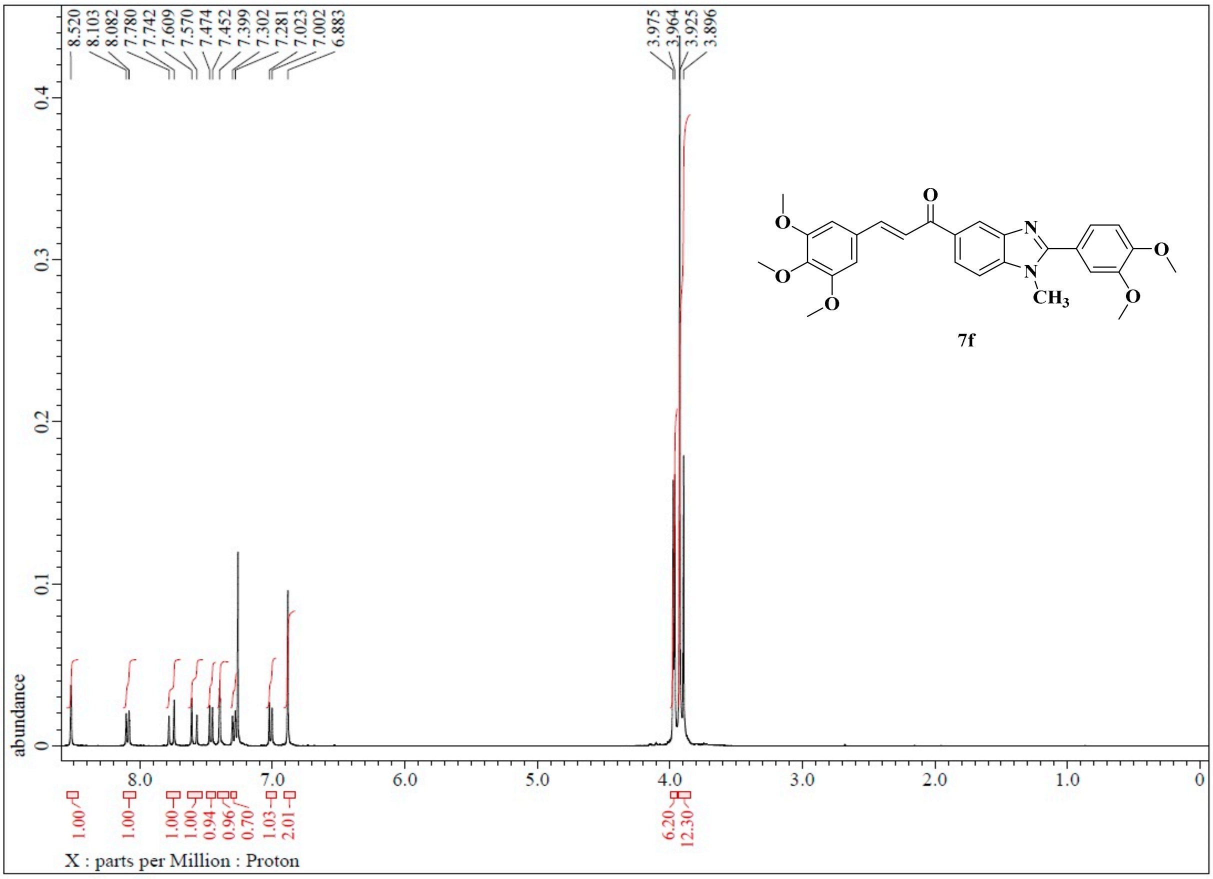

Figure S11. ^{1}H NMR spectrum of *(E)*-1-(2-(3,4-dimethoxyphenyl)-1-methyl-*1H*-benzo[*d*]imidazol-5-*yl*)-3-(3,4,5-trimethoxyphenyl)prop-2-en-1-one **(7f)**

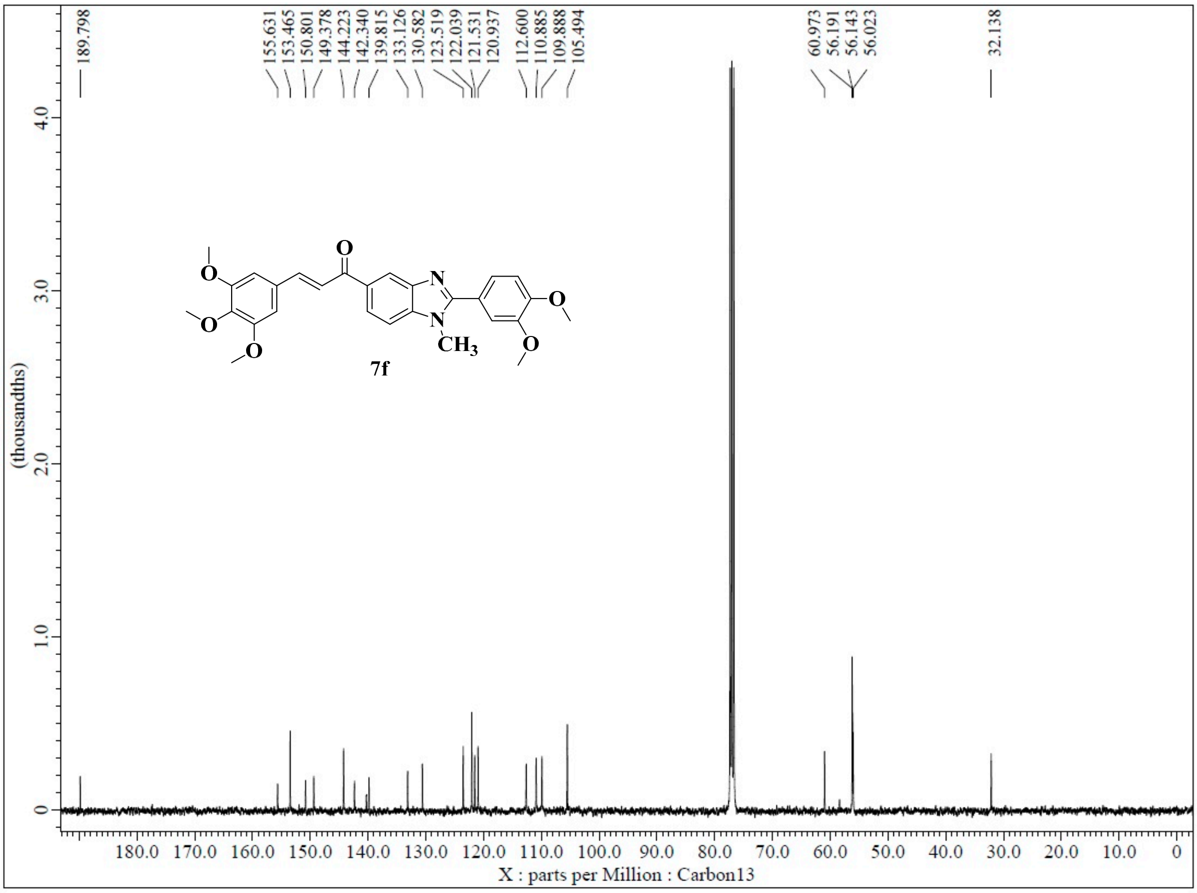

Figure S12. ^{13}C NMR spectrum of *(E)*-1-(2-(3,4-dimethoxyphenyl)-1-methyl-*1H*-benzo[*d*]imidazol-5-*yl*)-3-(3,4,5-trimethoxyphenyl)prop-2-en-1-one **(7f)**

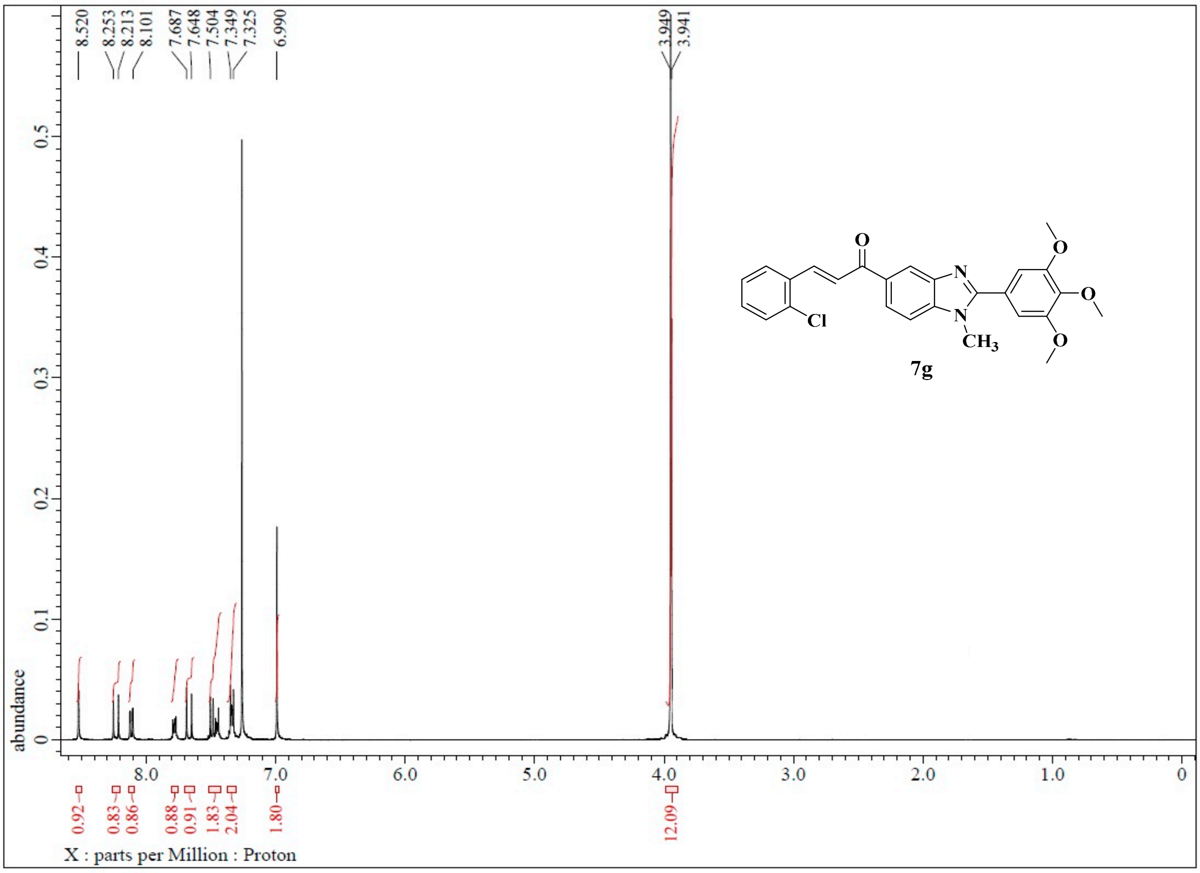

Figure S13. ¹H NMR spectrum of *(E)*-3-(2-chlorophenyl)-1-(1-methyl-2-(3,4,5-trimethoxyphenyl)-*1H*-benzo[*d*]imidazol-5-*yl*)prop-2-en-1-one **(7g)**

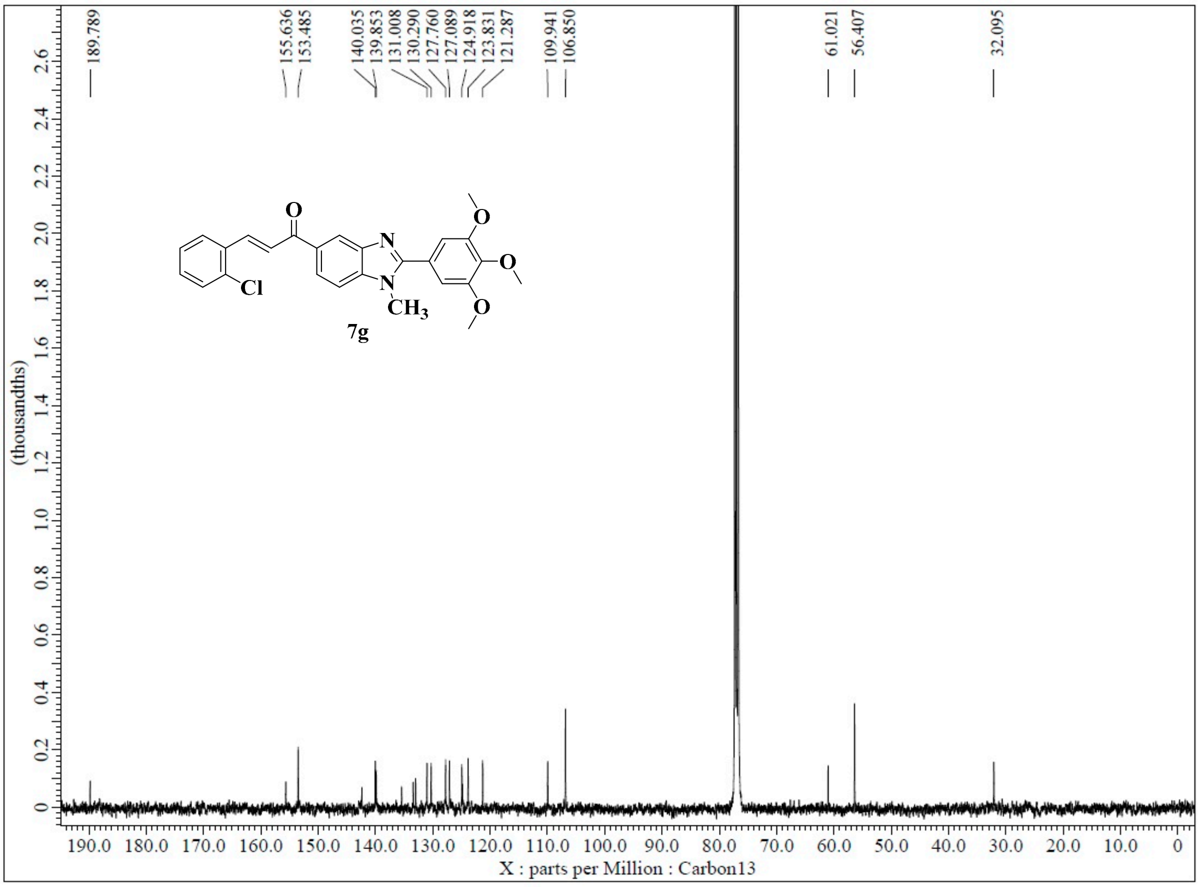

Figure S14. ^{13}C NMR spectrum of *(E)*-3-(2-chlorophenyl)-1-(1-methyl-2-(3,4,5-trimethoxyphenyl)-*1H*-benzo[*d*]imidazol-5-*yl*)prop-2-en-1-one **(7g)**

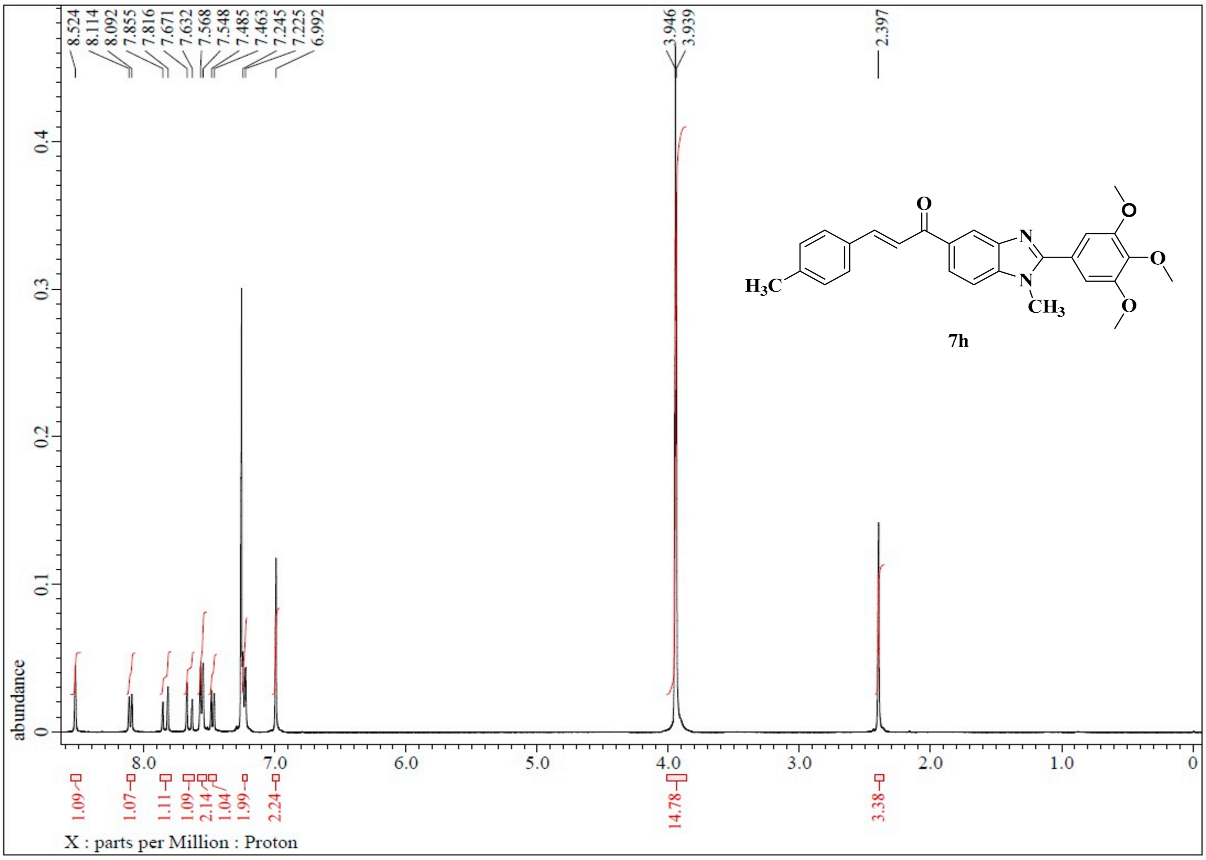

Figure S15. ^{1}H NMR spectrum of *(E)*-1-(1-methyl-2-(3,4,5-trimethoxyphenyl)-*1H*-benzo[*d*]imidazol-5-*yl*)-3-(p-tolyl)prop-2-en-1-one **(7h)**

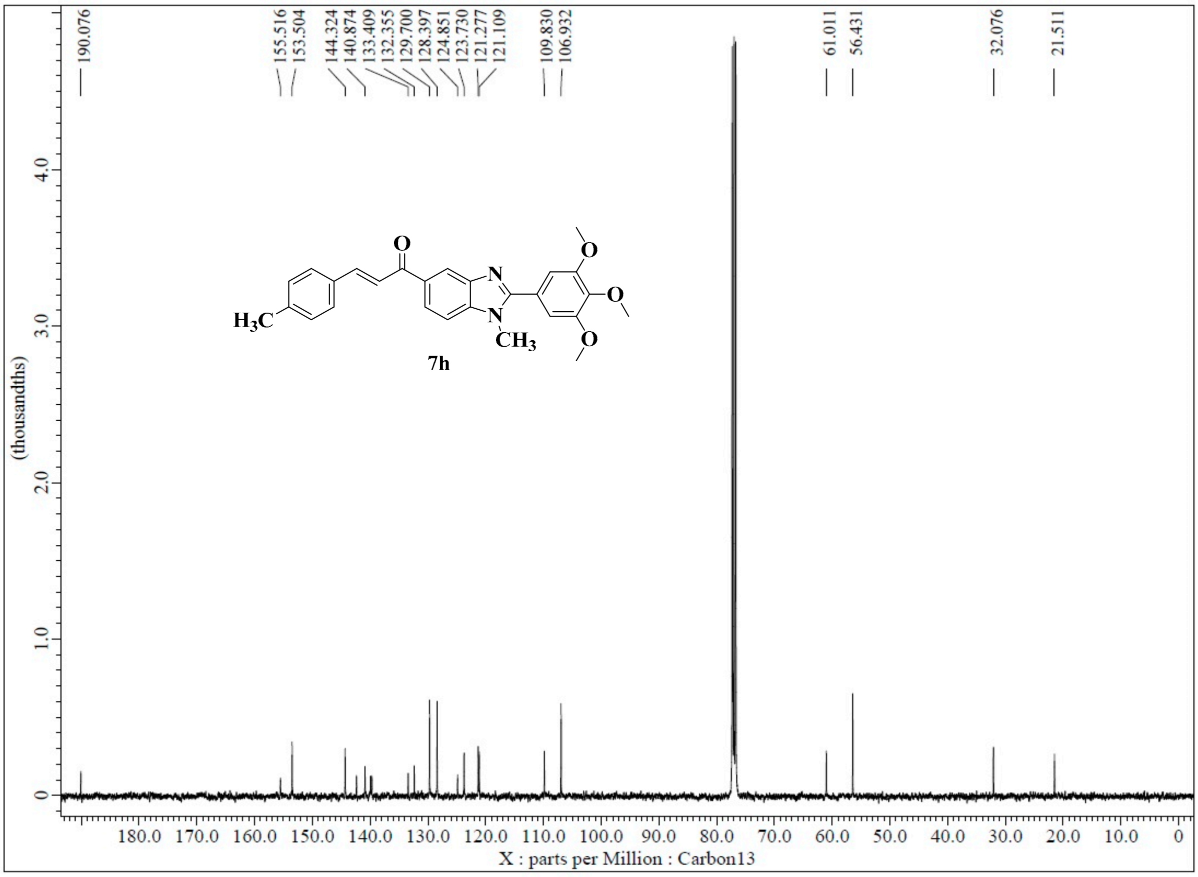

Figure S16. ^{13}C NMR spectrum of *(E)*-1-(1-methyl-2-(3,4,5-trimethoxyphenyl)-*1H*-benzo[*d*]imidazol-5-*yl*)-3-(p-tolyl)prop-2-en-1-one **(7h)**

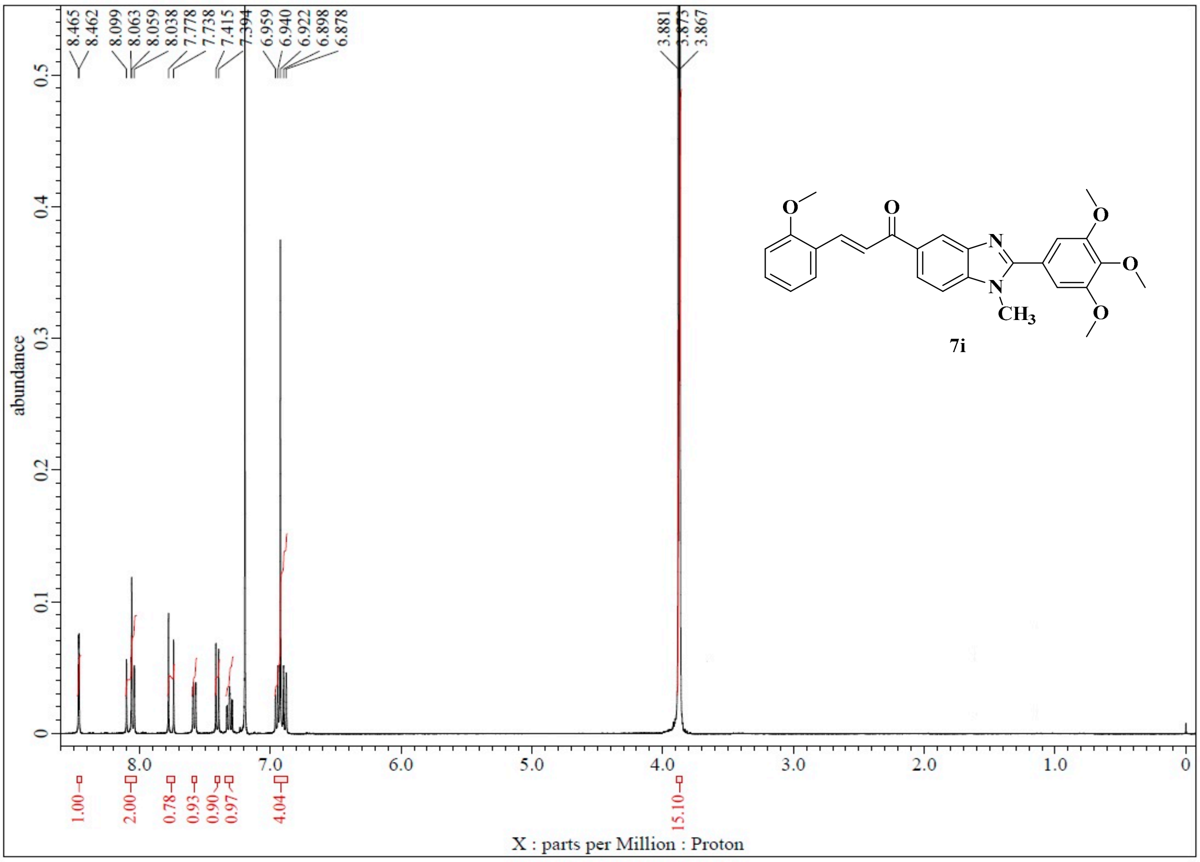

Figure S17. ^{1}H NMR spectrum of *(E)*-3-(2-methoxyphenyl)-1-(1-methyl-2-(3,4,5-trimethoxyphenyl)-*1H*-benzo[*d*]imidazol-5-*yl*)prop-2-en-1-one **(7i)**

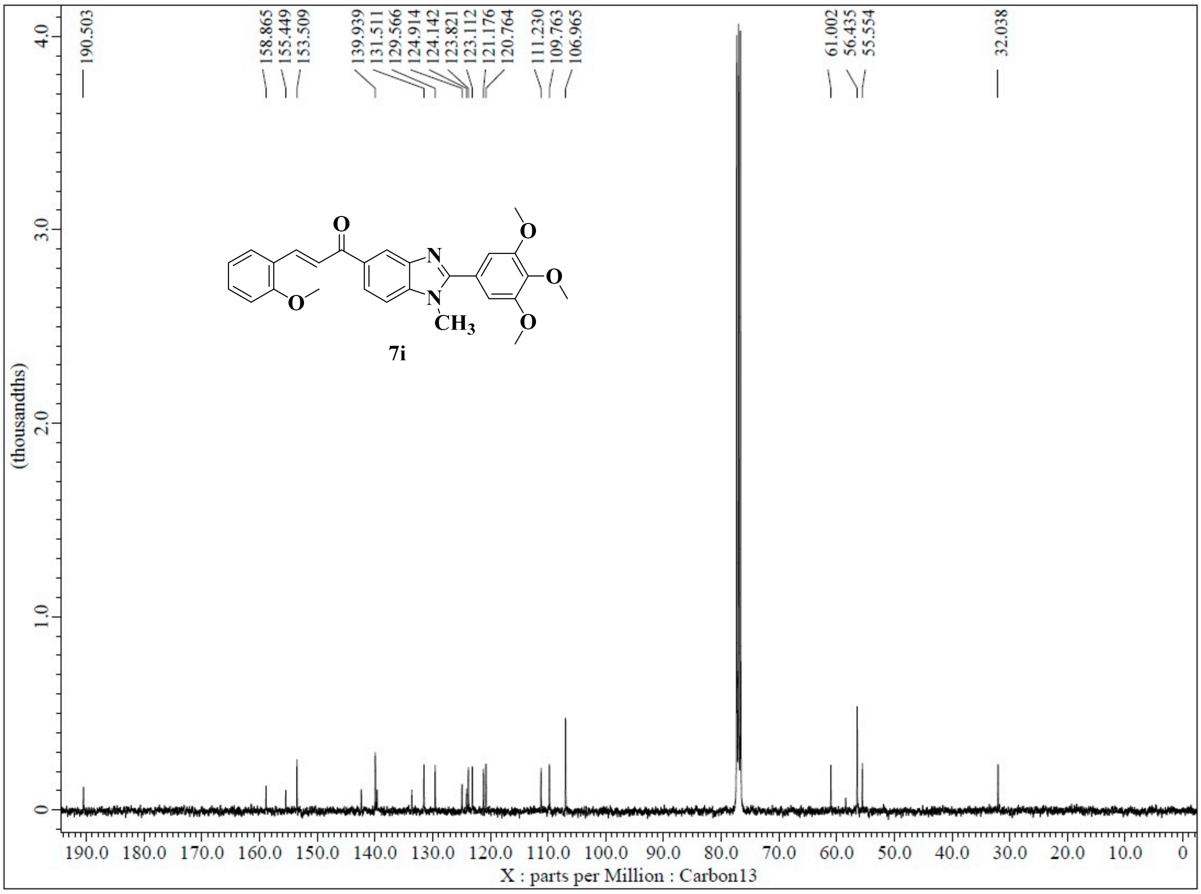

Figure S18. ^{13}C NMR spectrum of *(E)*-3-(2-methoxyphenyl)-1-(1-methyl-2-(3,4,5-trimethoxyphenyl)-*1H*-benzo[*d*]imidazol-5-*yl*)prop-2-en-1-one **(7i)**

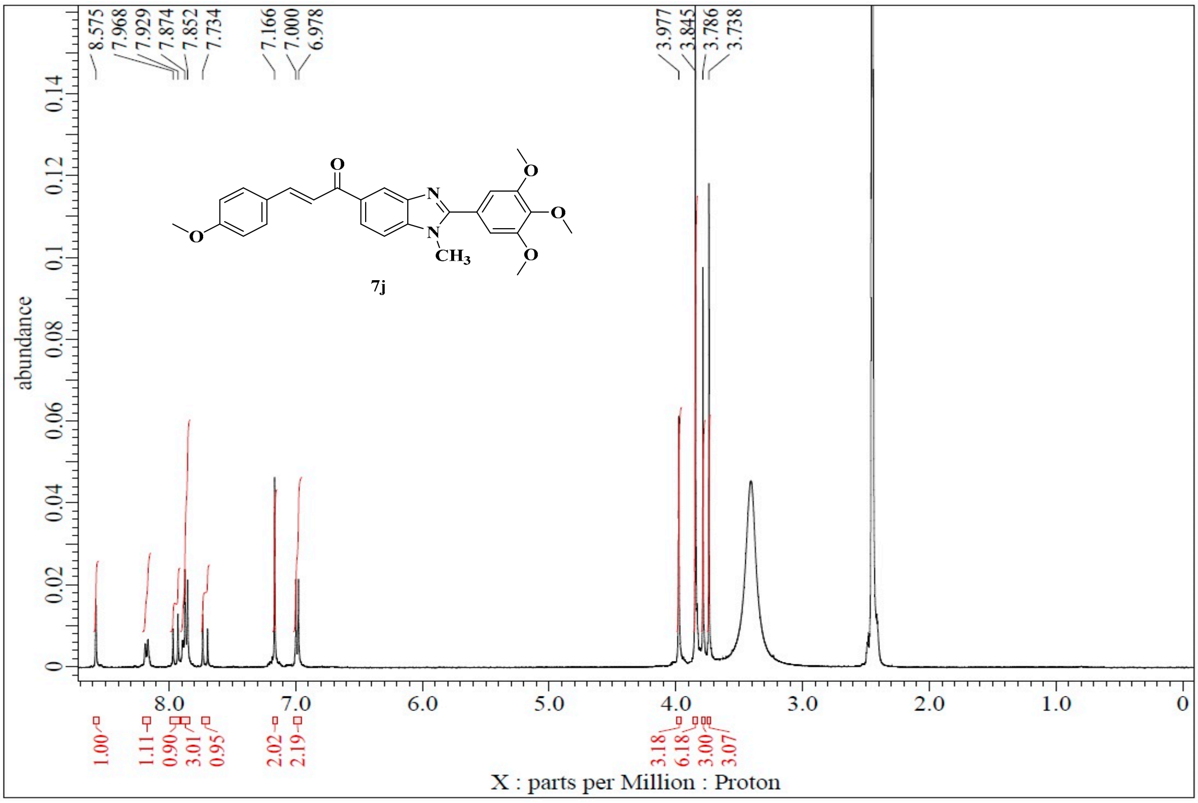

Figure S19. ^{1}H NMR spectrum of (E)-3-(4-methoxyphenyl)-1-(1-methyl-2-(3,4,5-trimethoxyphenyl)-1H-benzo[d]imidazol-5-yl)prop-2-en-1-one **(7j)**

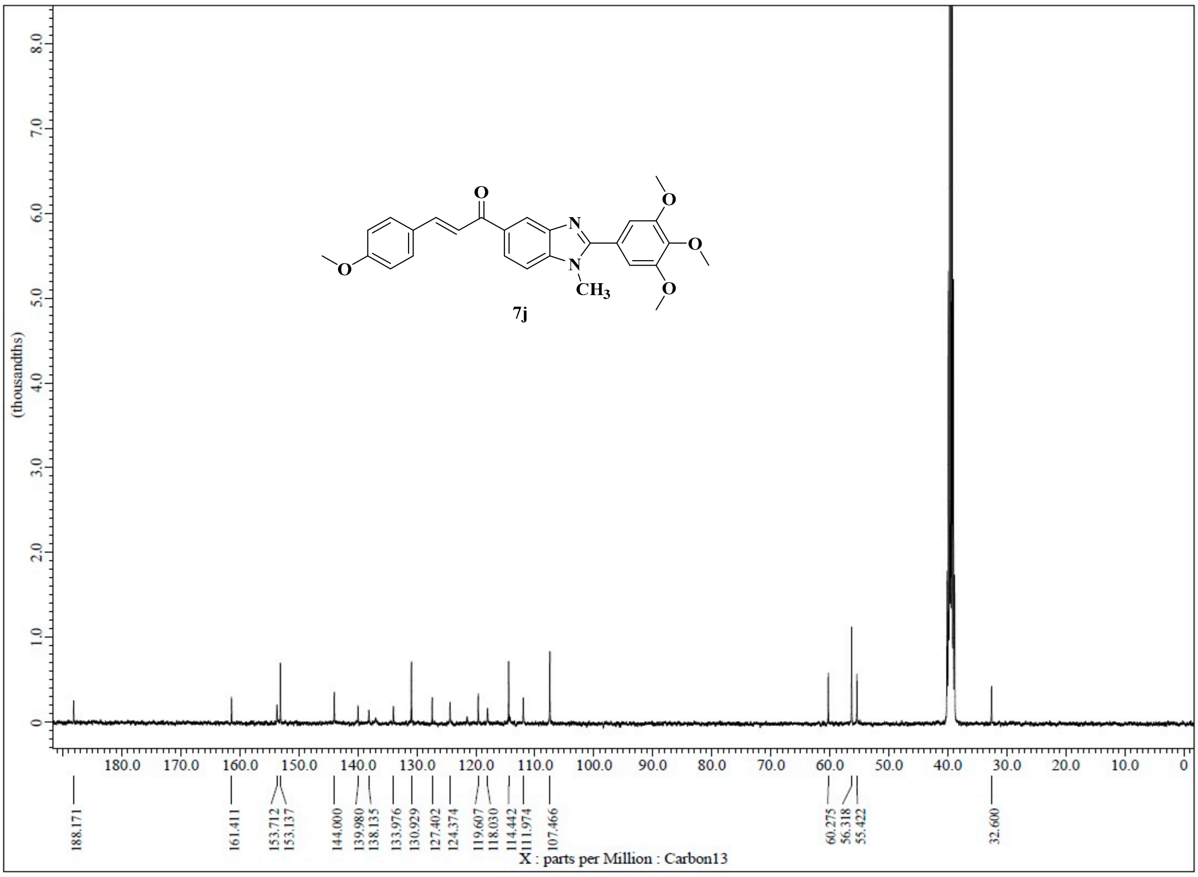

Figure S20. ^{13}C NMR spectrum of (E)-3-(4-methoxyphenyl)-1-(1-methyl-2-(3,4,5-trimethoxyphenyl)-1H-benzo[d]imidazol-5-yl)prop-2-en-1-one **(7j)**

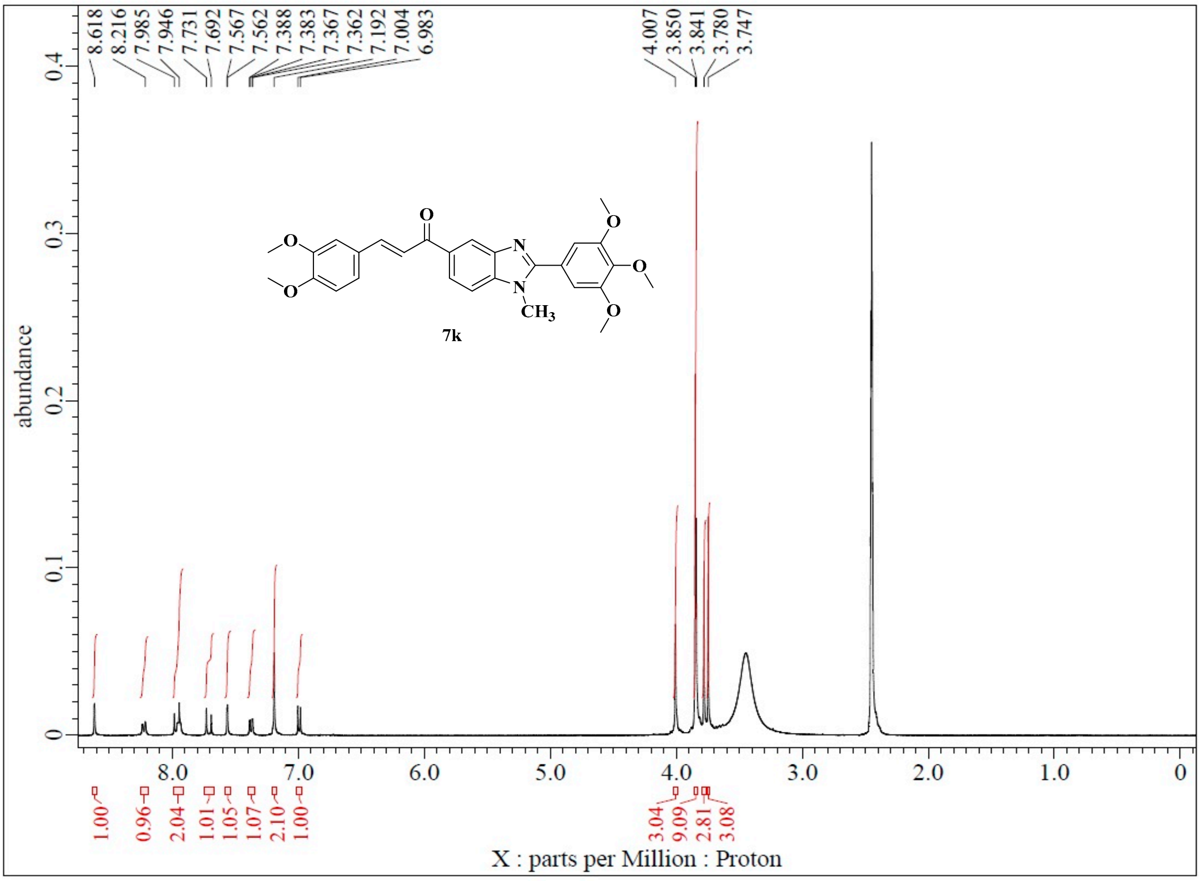

Figure S21. ^{1}H NMR spectrum of (E)-3-(3,4-dimethoxyphenyl)-1-(1-methyl-2-(3,4,5-trimethoxyphenyl)-1H-benzo[d]imidazol-5-yl)prop-2-en-1-one (**7k)**

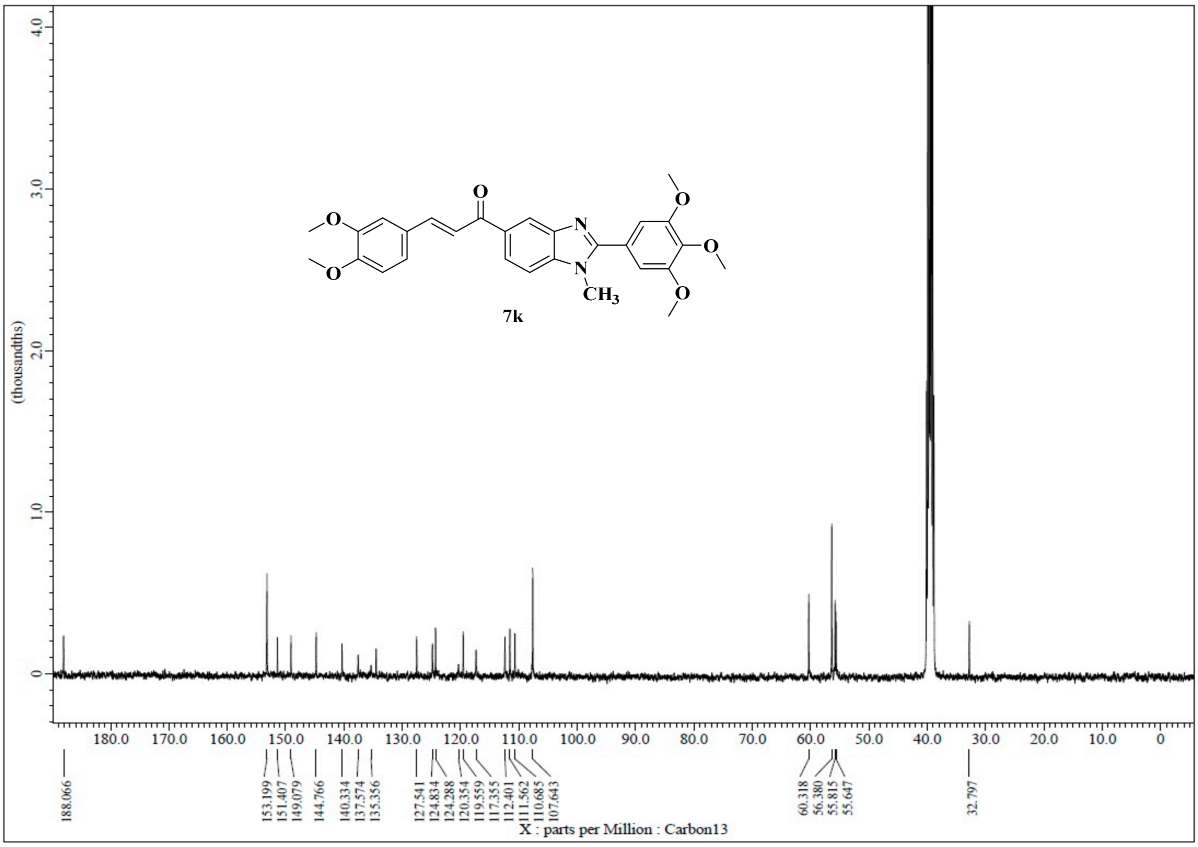

Figure S22. ^{13}C NMR spectrum of (E)-3-(3,4-dimethoxyphenyl)-1-(1-methyl-2-(3,4,5-trimethoxyphenyl)-1H-benzo[d]imidazol-5-yl)prop-2-en-1-one **(7k)**

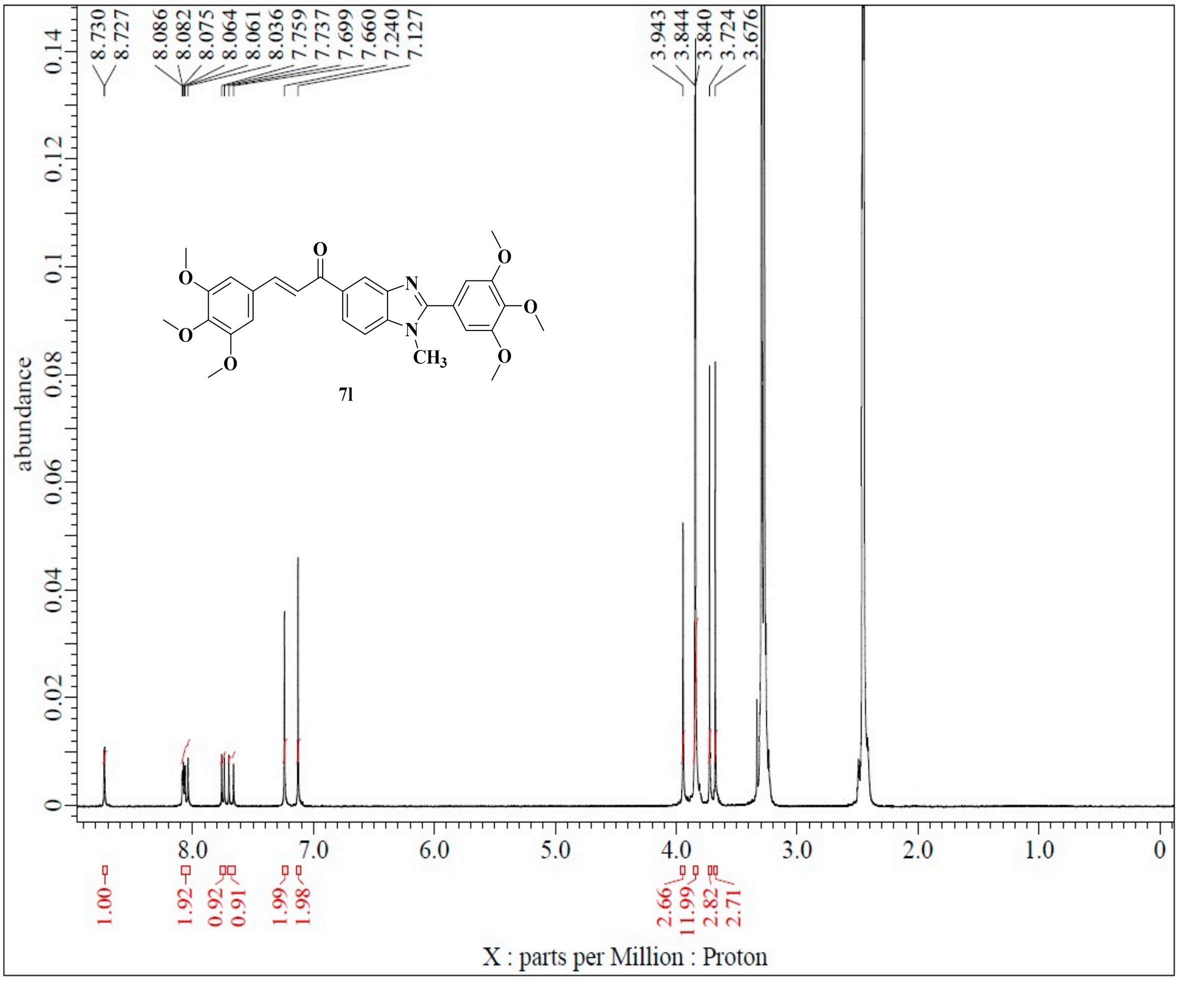

Figure S23. ^{1}H NMR spectrum of (E)-1-(1-methyl-2-(3,4,5-trimethoxyphenyl)-1H-benzo[d]imidazol-5-yl)-3-(3,4,5-trimethoxyphenyl)prop-2-en-1-one **(7l)**

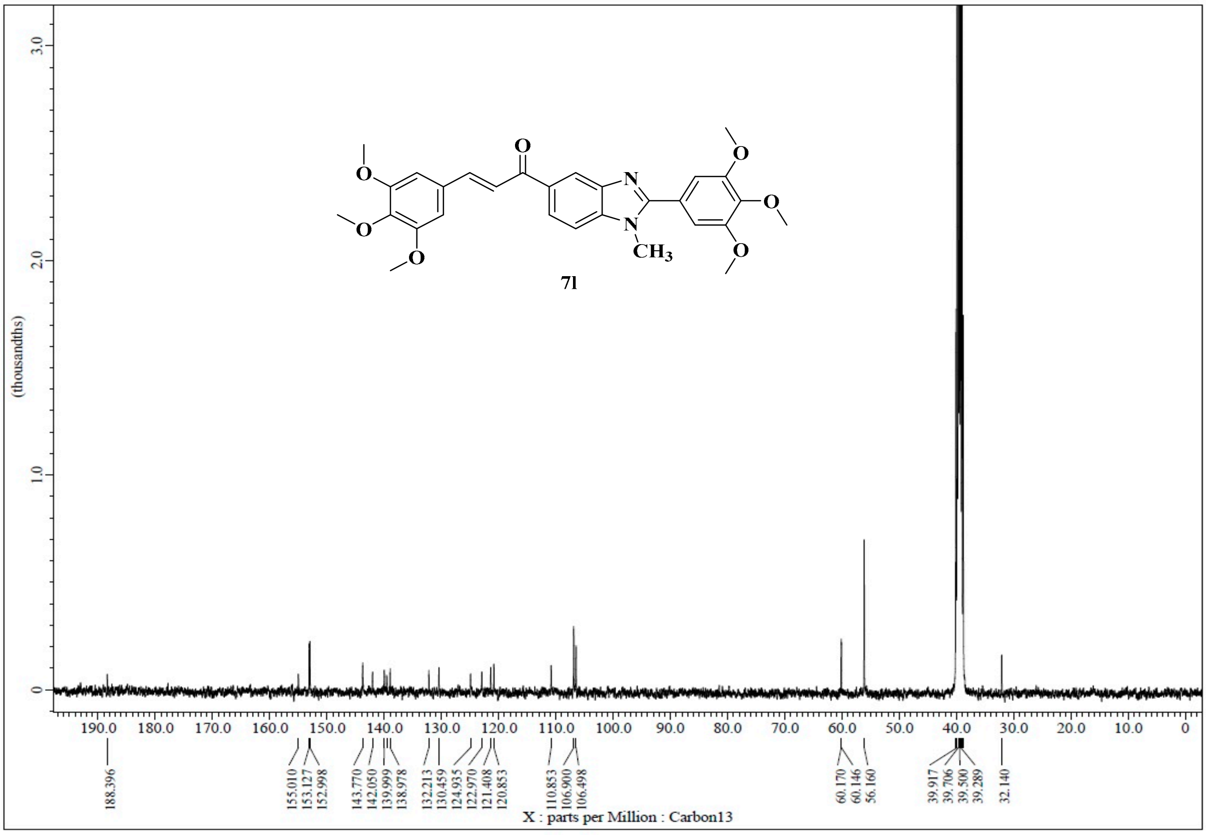

Figure S24. ^{13}C NMR spectrum of (E)-1-(1-methyl-2-(3,4,5-trimethoxyphenyl)-1H-benzo[d]imidazol-5-yl)-3-(3,4,5-trimethoxyphenyl)prop-2-en-1-one **(7l)**

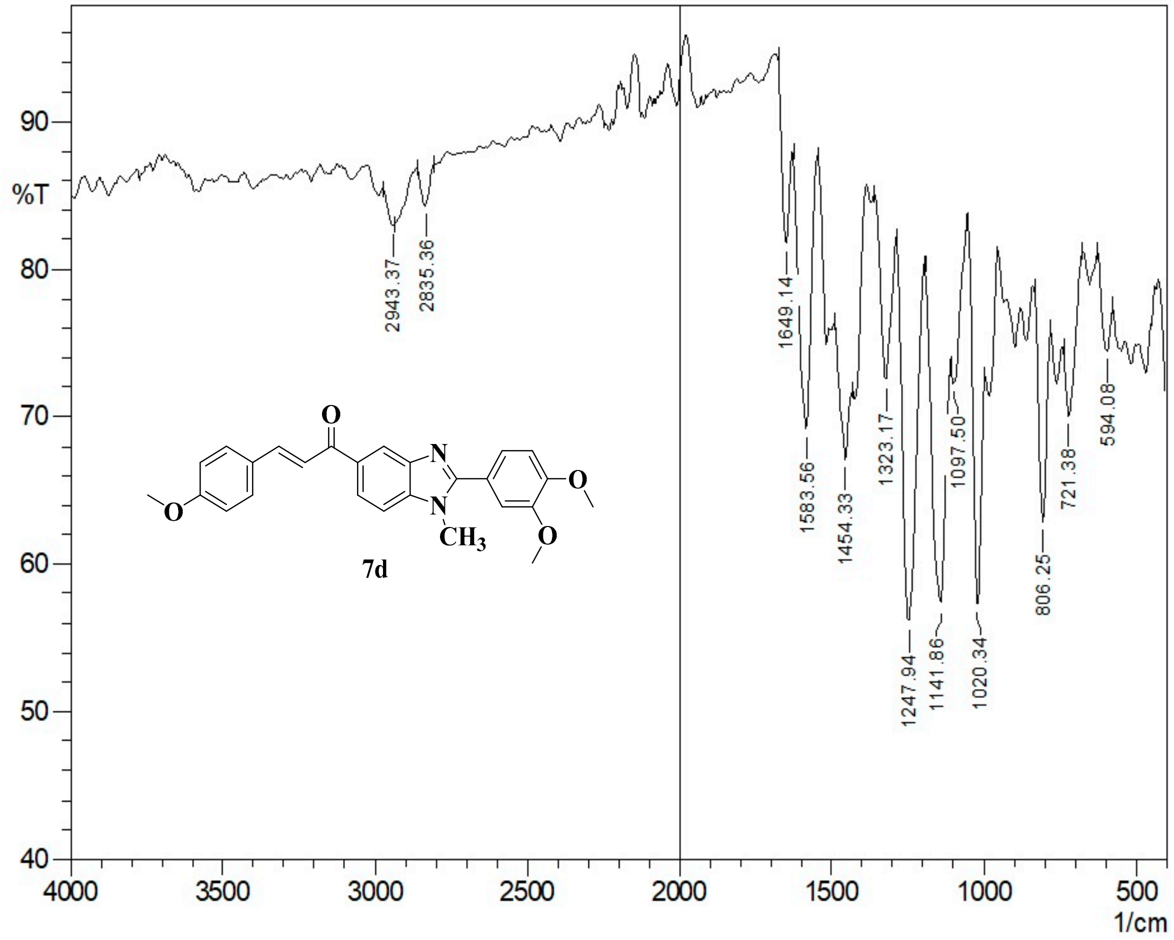

Figure S25. FT IR spectrum of *(E)*-1-(2-(3,4-dimethoxyphenyl)-1-methyl-*1H*-benzo[*d*]imidazol-5-*yl*)-3-(4-methoxyphenyl)prop-2-en-1-one **(7d)**

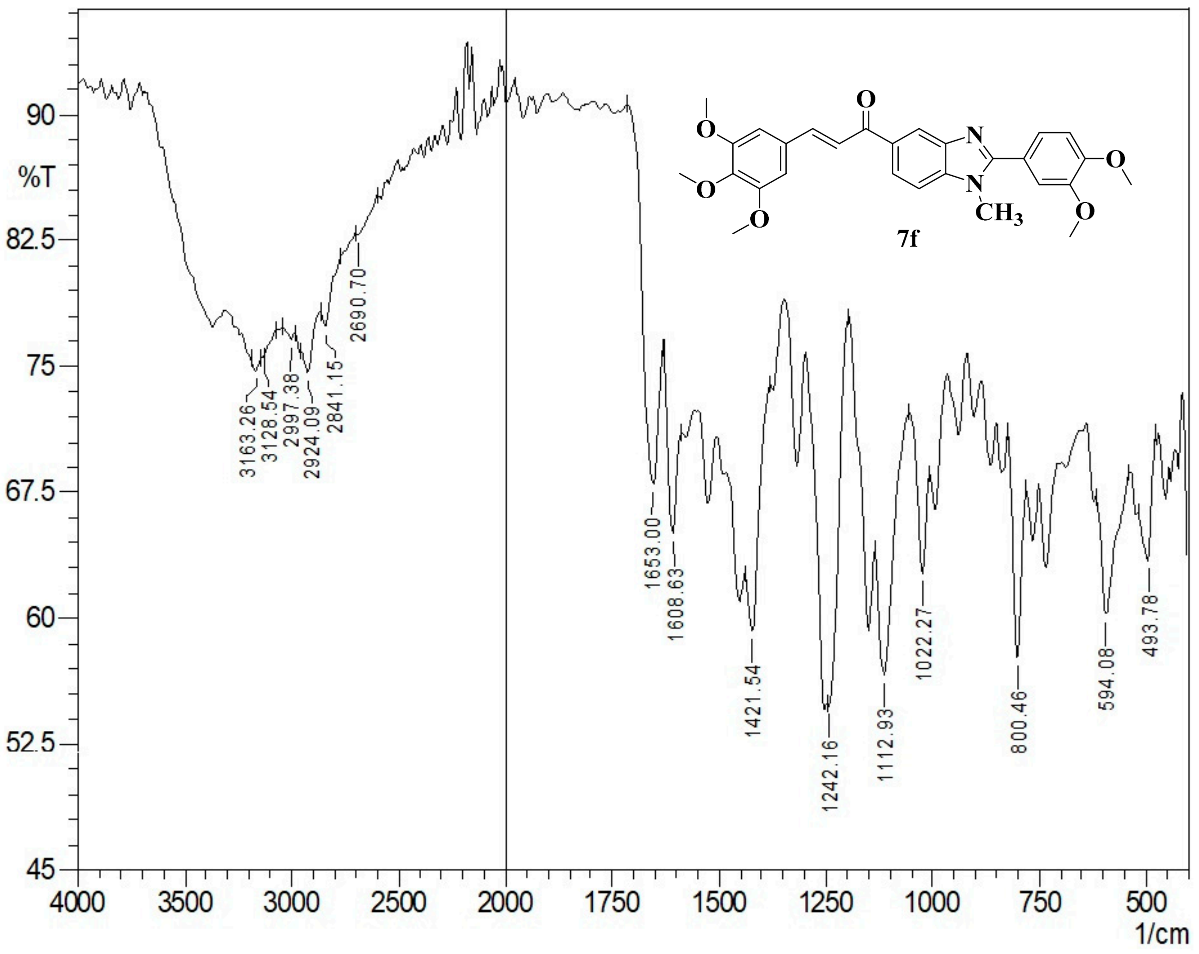

Figure S26. FT IR spectrum of *(E)*-1-(2-(3,4-dimethoxyphenyl)-1-methyl-*1H*-benzo[*d*]imidazol-5-*yl*)-3-(3,4,5-trimethoxyphenyl)prop-2-en-1-one **(7f)**

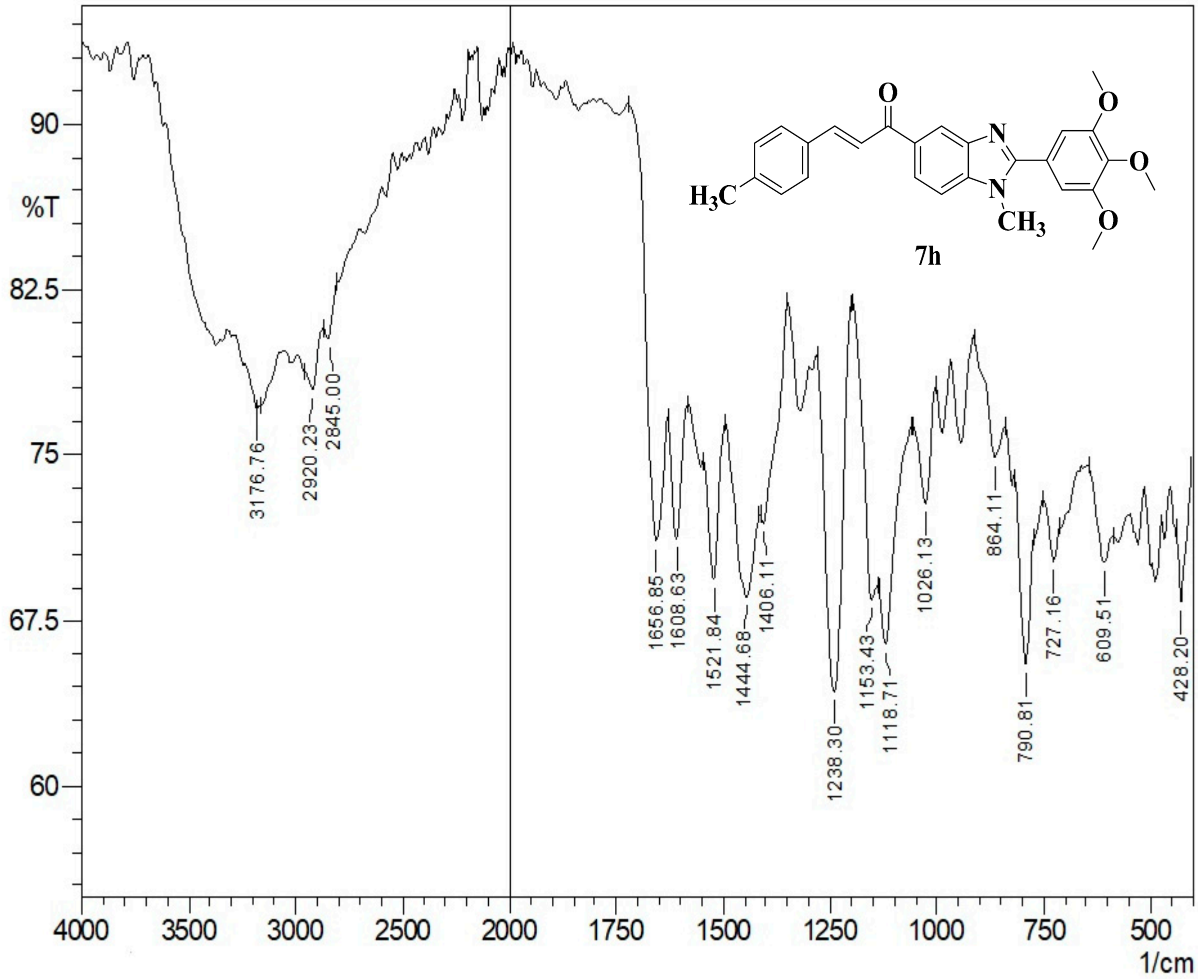

Figure S27. FT IR spectrum of *(E)*-1-(1-methyl-2-(3,4,5-trimethoxyphenyl)-*1H*-benzo[*d*]imidazol-5-*yl*)-3-(p-tolyl)prop-2-en-1-one **(7h)**

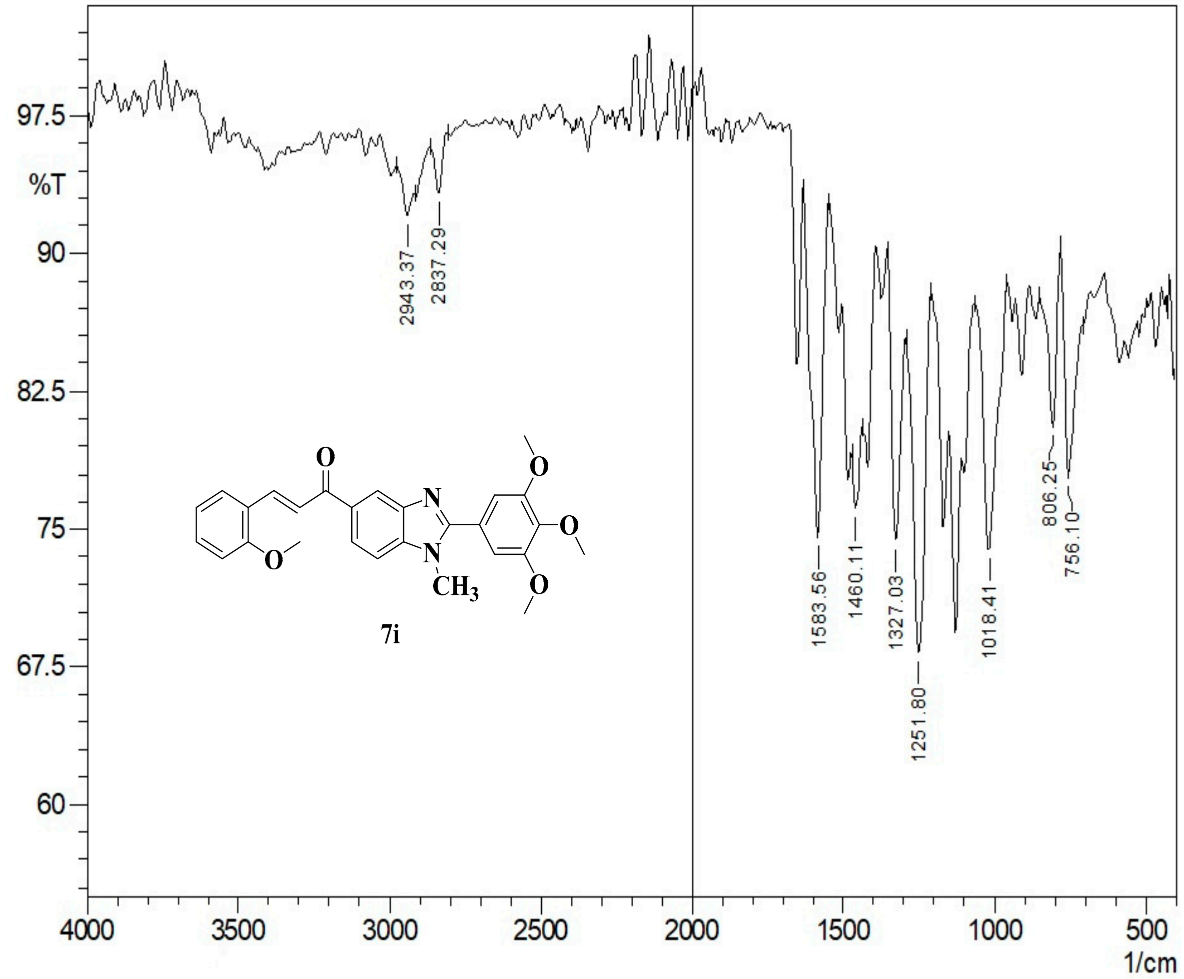

Figure S28. FT IR spectrum of *(E)*-3-(2-methoxyphenyl)-1-(1-methyl-2-(3,4,5-trimethoxyphenyl)-*1H*-benzo[*d*]imidazol-5-*yl*)prop-2-en-1-one **(7i)**

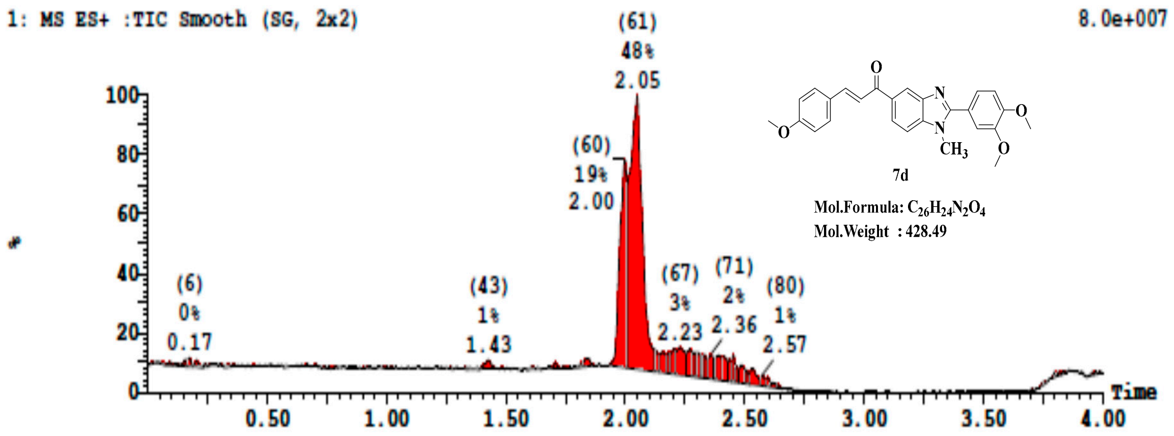

Peak ID	Compound	Time	Mass Found
61		2.05	Not Found

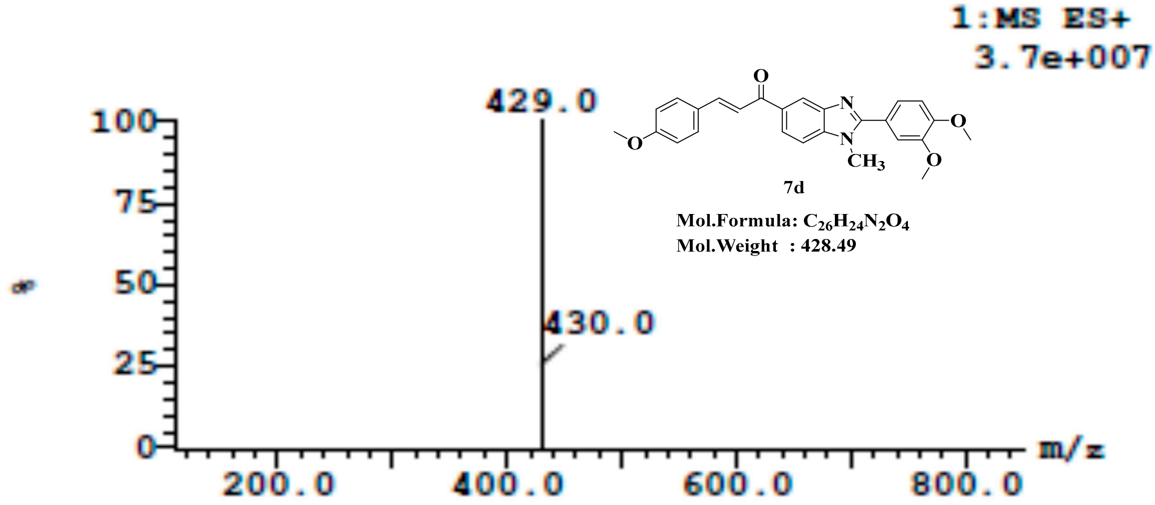

Figure S29. Mass spectrum of *(E)*-1-(2-(3,4-dimethoxyphenyl)-1-methyl-*1H*-benzo[*d*]imidazol-5-*yl*)-3-(4-methoxyphenyl)prop-2-en-1-one **(7d)**

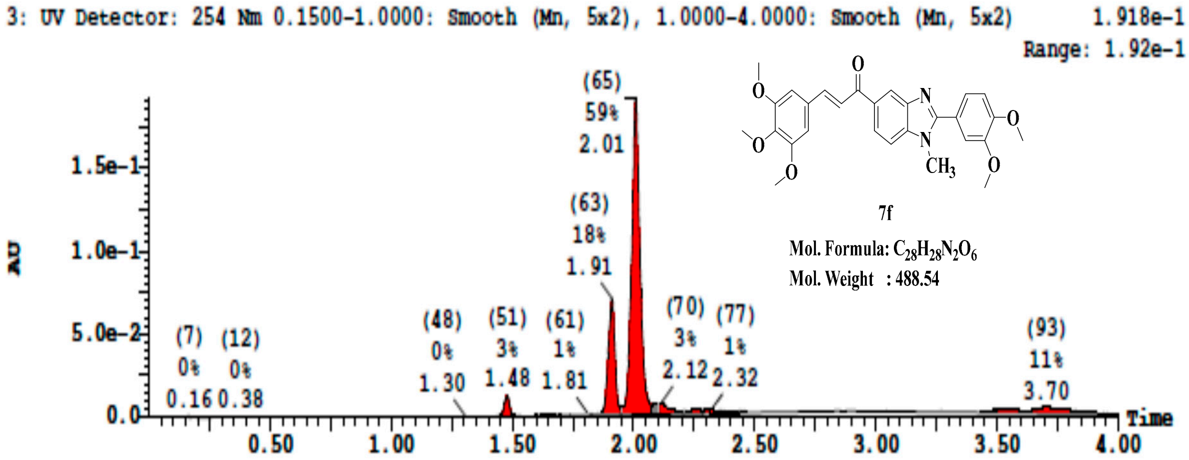

Peak ID	Compound	Time	Mass Found
65		2.01	Not Found

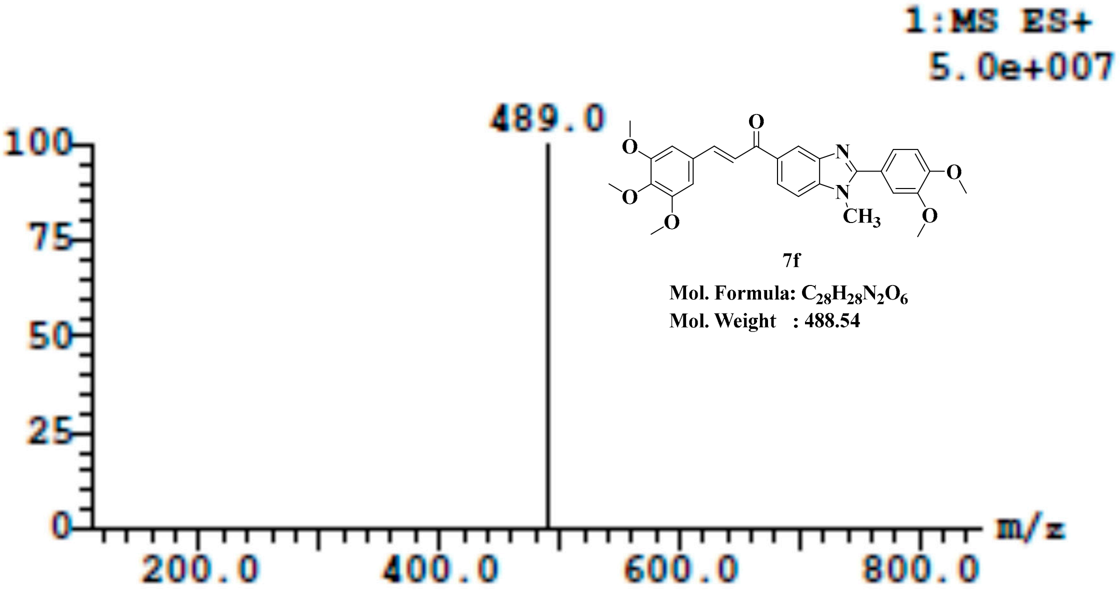

Figure S30. Mass spectrum of *(E)*-1-(2-(3,4-dimethoxyphenyl)-1-methyl-*1H*-benzo[*d*]imidazol-5-*yl*)-3-(3,4,5-trimethoxyphenyl)prop-2-en-1-one **(7f)**

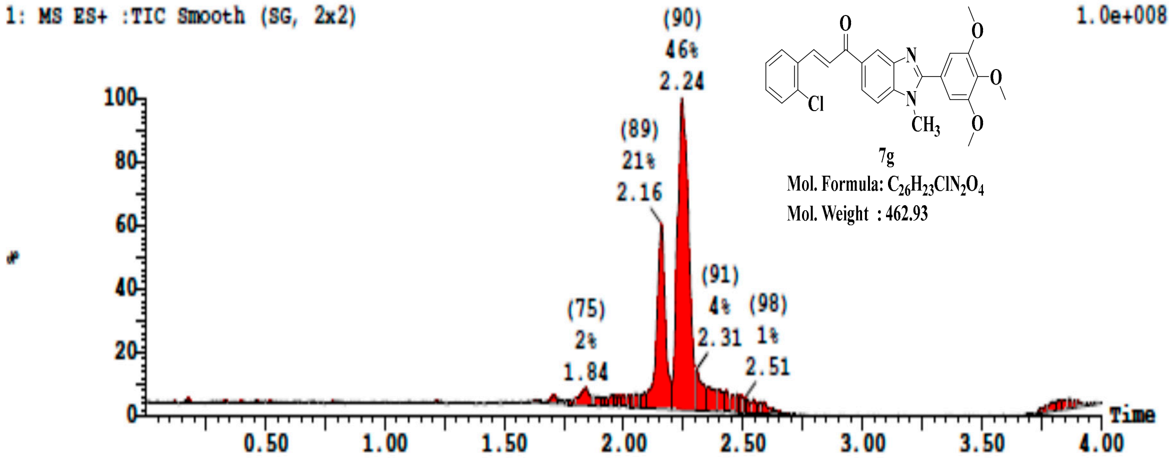

Peak ID	Compound	Time	Mass Found
90		2.24	Not Found

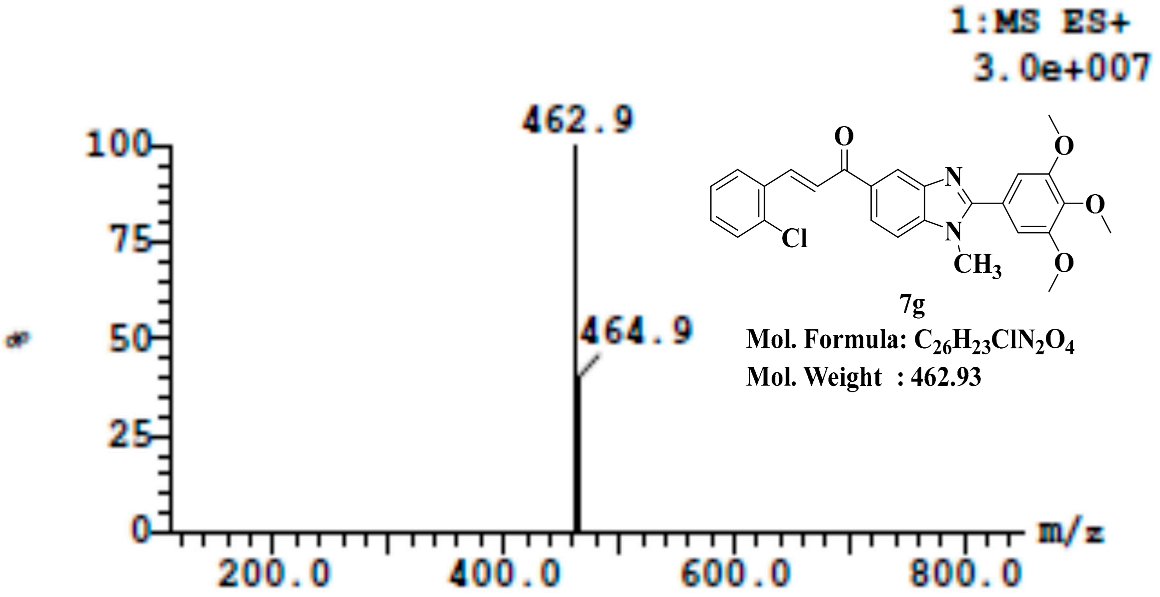

Figure S31. Mass spectrum of *(E)*-3-(2-chlorophenyl)-1-(1-methyl-2-(3,4,5-trimethoxyphenyl)-*1H*-benzo[*d*]imidazol-5-*yl*)prop-2-en-1-one **(7g)**

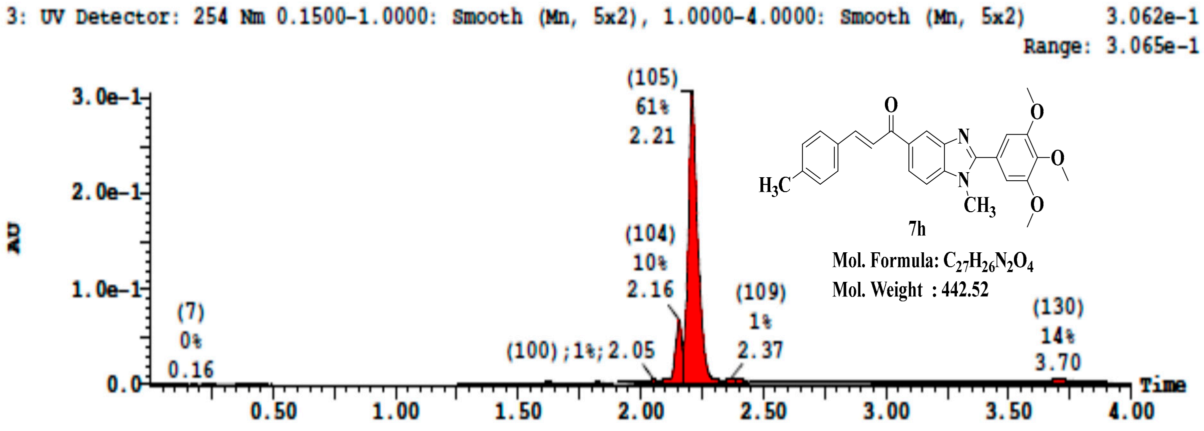

Peak ID	Compound	Time	Mass Found
60		2.10	Not Found

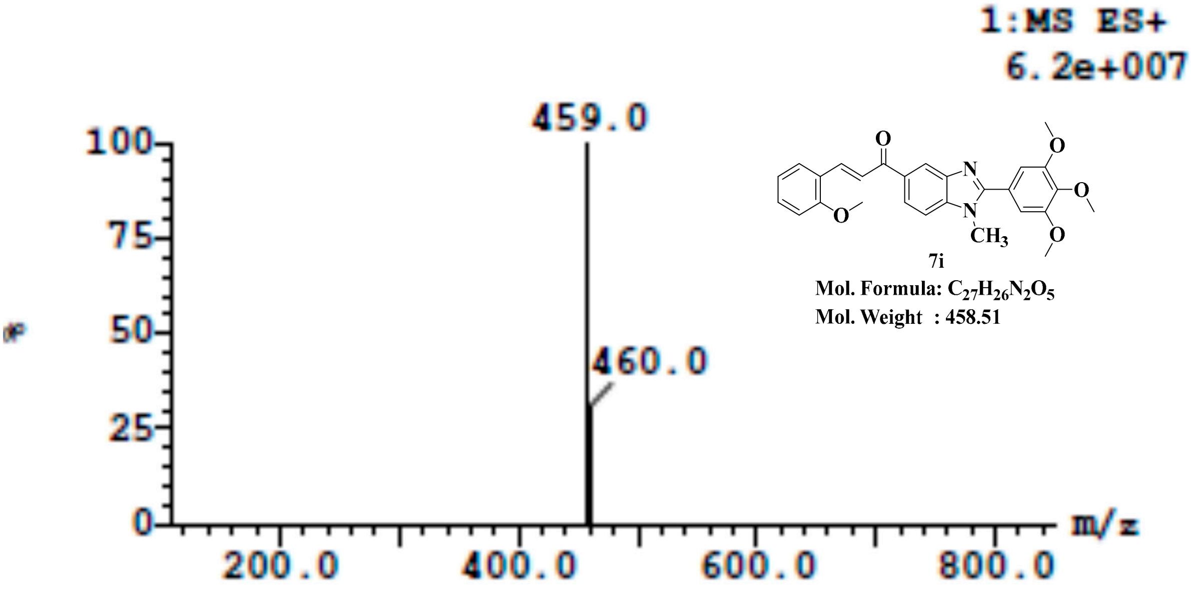

Figure S32. Mass spectrum of *(E)*-1-(1-methyl-2-(3,4,5-trimethoxyphenyl)-*1H*-benzo[*d*]imidazol-5-*y*l)-3-(p-tolyl)prop-2-en-1-one **(7h)**

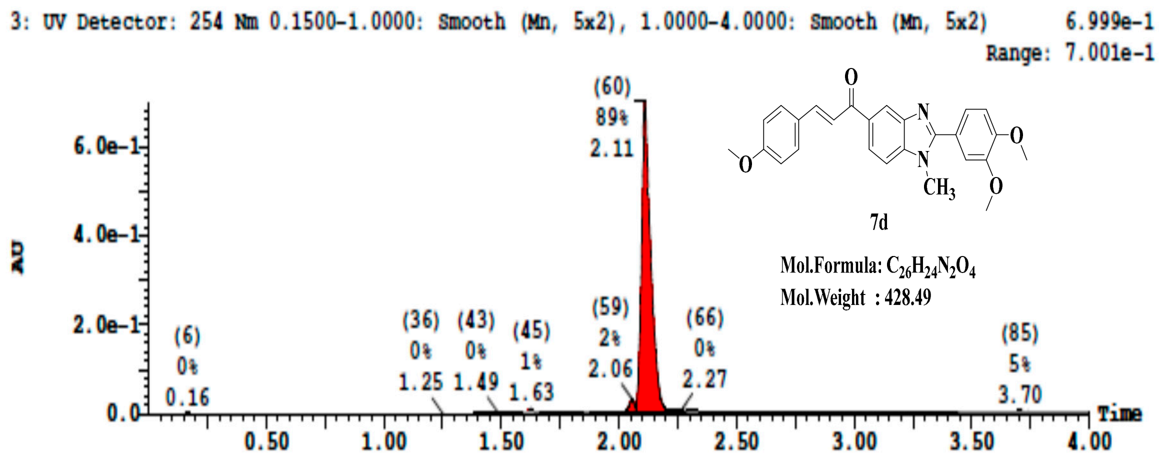

Peak ID	Compound	Time	Mass Found
105		2.21	Not Found

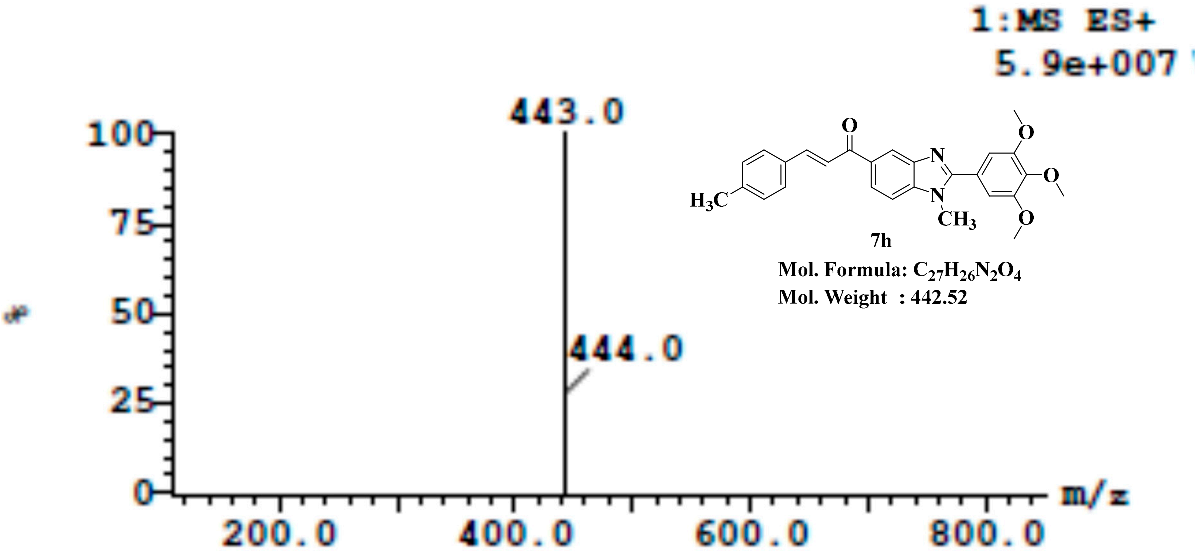

Figure S33. Mass spectrum of *(E)*-3-(2-methoxyphenyl)-1-(1-methyl-2-(3,4,5-trimethoxyphenyl)-*1H*-benzo[*d*]imidazol-5-*yl*)prop-2-en-1-one **(7i)**
